# A straightforward method for automated Fmoc-based synthesis of bio-inspired peptide crypto-thioesters†
†Electronic supplementary information (ESI) available: Detailed synthetic procedures, characterization and kinetics studies. See DOI: 10.1039/c5sc02630j


**DOI:** 10.1039/c5sc02630j

**Published:** 2015-09-23

**Authors:** Victor P. Terrier, Hélène Adihou, Mathieu Arnould, Agnès F. Delmas, Vincent Aucagne

**Affiliations:** a Centre de Biophysique Moléculaire , CNRS UPR 4301 , Rue Charles Sadron , 45071 Orléans Cedex 2 , France . Email: aucagne@cnrs-orleans.fr

## Abstract

Despite recent advances, the direct Fmoc-based solid phase synthesis of peptide α-thioesters for the convergent synthesis of proteins *via* native chemical ligation (NCL) remains a challenge in the field. We herein report a simple and general methodology, enabling access to peptide thioester surrogates. A novel C-terminal *N*-(2-hydroxybenzyl)cysteine thioesterification device based on an amide-to-thioester rearrangement was developed, and the resulting peptide crypto-thioesters can be directly used in NCL reactions with fast *N* → *S* shift kinetics at neutral pH. These fast kinetics arise from our bio-inspired design, *via* intein-like intramolecular catalysis. Due to a well-positioned phenol moiety, an impressive >50 fold increase in the kinetic rate is observed compared to an *O*-methylated derivative. Importantly, the synthesis of this new device can be fully automated using inexpensive commercially available materials and does not require any post-synthetic steps prior to NCL. We successfully applied this new method to the synthesis of two long naturally-occurring cysteine-rich peptide sequences.

## Abstract 2

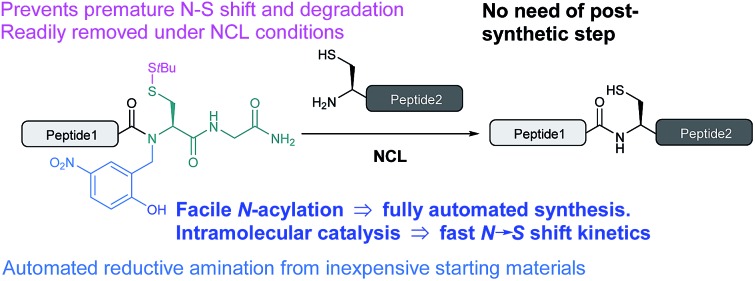
A bio-inspired method for the synthesis of peptide thioester surrogates for native chemical ligation was developed. The process can be fully automated and does not require postsynthetic steps.

## Introduction

Peptide α-thioesters are key intermediates for the convergent synthesis of proteins through native chemical ligation (NCL), a reaction that has revolutionized the field.^1^ While peptide thioesters can be directly synthesized through Boc-based solid-phase peptide synthesis (SPPS), their access *via* the more widely used Fmoc-based strategy is not straightforward due to the instability of the thioester moiety to repeated piperidine treatments used for Fmoc deprotection. Most Fmoc-based methodologies developed to date rely on a post-SPPS conversion of a piperidine-stable precursor into a thioester, but despite considerable efforts, no universal strategy has emerged.^2^ Thanks to their simple implementation, methods such as Dawson's *N*-acylurea (Nbz)^3^ and Liu's hydrazides^4^ for the generation of thioester precursors have recently become popular, but are still associated with several limitations.^5^ Thus, a reliable and straightforward route to peptide α-thioesters *via* Fmoc-SPPS is highly desirable.

**Fig. 1:**
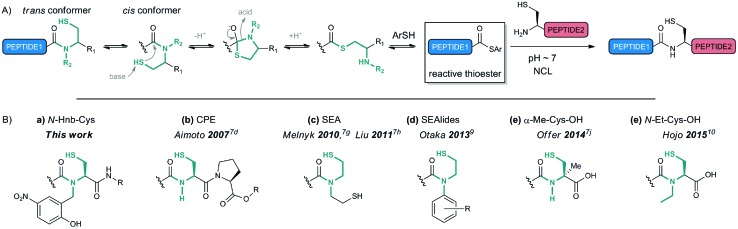
*N* → *S* shift-based *in situ* synthesis of peptide thioesters for NCL. (A) Mechanism. (B) Crypto-thioesters bearing thioesterification devices operating under NCL conditions.

A number of strategies based on β- or γ-mercapto amide thioesterification devices have recently appeared that exploit an amide-to-thioester rearrangement^6^,^7^ (Fig. 1A), which is reminiscent of the first step of intein-promoted *in vivo* protein splicing.^8^ In most cases, the *N* → *S* acyl shift is reversible and requires acidic conditions to proceed efficiently, meaning that the peptides have to be first converted into thioesters prior to NCL, which is optimal at pH ∼ 7. A few systems, referred to as crypto-thioesters, are able to operate under NCL conditions, thus enabling one-pot reactions (thioester formation and NCL). Cysteine-prolyl esters (CPEs) developed by Aimoto7*d* (Fig. 1B-b) rely on an elegant intramolecular *O* → *N* shift to displace the amide–thioester equilibrium under NCL conditions, but with slow ligation kinetics. Although simple to implement, the α-methyl cysteine crypto-thioesters recently introduced by Offer7*j* (Fig. 1B-e) are impaired by similarly slow kinetics. In both cases, the limiting factor is likely the disfavored *trans*-to-*cis* isomerization of the amide bond to generate the geometrically feasible intramolecular attack of the thiolate. Use of *N*,*N*-disubstituted β-mercapto amides can overpass this limitation by disfavoring the unproductive *trans* conformer. The symmetrical bis-sulfanylethylamides (SEAs, Fig. 1B-c) introduced by Melnyk7*g* and Liu7*h* can be used in NCL at pH 6–7 with relatively fast kinetics, but work better at lower pH. Otaka's *N*-sulfanylethylanilides7*e* (Sealides, Fig. 1B-d) exploit the electronic effect of the anilide to enhance the electrophilicity of the amide bond. They are efficient NCL substrates at neutral pH only when using a phosphate-based buffer, probably arising from pseudo-intramolecular acid–base catalysis, but the exact mechanism remains elusive.^9^ Interestingly, while we were preparing this paper, a method appeared based on an *N*-ethyl cysteine device featuring a free carboxylic acid; peptides equipped with this device can act as crypto-thioesters. The α-acid group was shown to accelerate the reaction compared to an α-amide-containing analogue, with optimum ligation kinetics at pH 5.5, but with very slow kinetics at pH 7.^10^

We herein report the design and optimization of a simple and general methodology based on an *N*-(2-hydroxybenzyl)cysteine device, which can be automatically assembled in the solid-phase using inexpensive commercially available materials. Peptides bearing this device show fast *N* → *S* shift kinetics under NCL conditions around neutral pH, likely arising from a biomimetic intein-like intramolecular catalysis mechanism. The synthesis of these novel crypto-thioesters is straightforward and does not require an additional step prior to NCL. We believe that this accessible and robust Fmoc-based thioesterification device provides a significant advance to chemical protein synthesis.

## Results and discussion

**Fig. 2:**
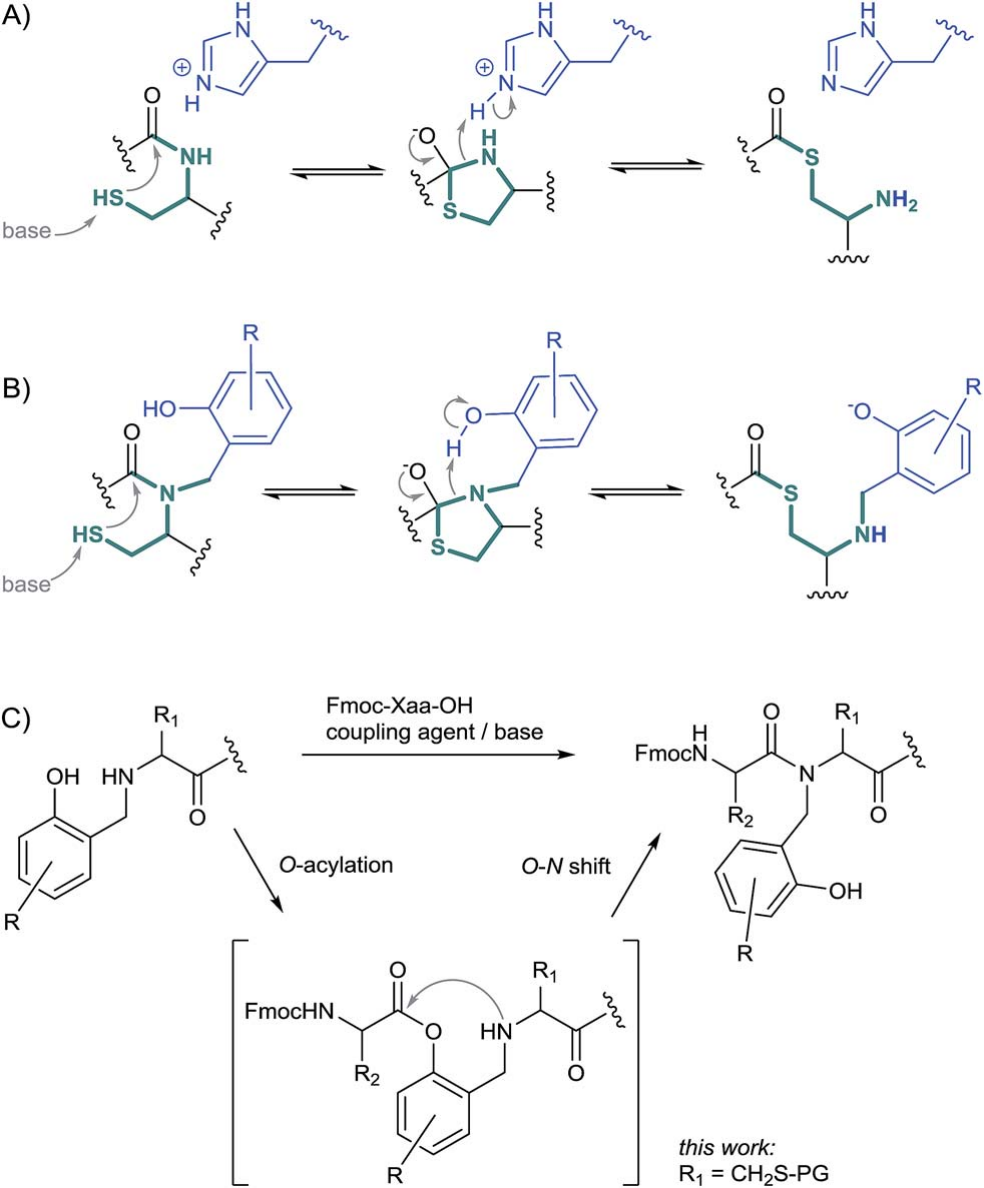
Rational design of *N*-(2-hydroxybenzyl)cysteine thioesterification devices. (A) Mechanism of the intein self-catalyzed *N* → *S* shift in water. (B) Bio-inspired putative mechanism for the self-catalysis of the *N* → *S* shift in *N*-acyl-*N*-(2-hydroxybenzyl)cysteine peptides in water. (C) Mechanism of the intramolecular *O* → *N* shift in organic solvents shared by *N*-Hmb peptides and the new *N*-(2-hydroxybenzyl)cysteine thioesterification devices. PG: protecting group.

We based our design on *N*-alkyl cysteine7*c* as a scaffold for the design of our thioesterification device, as it can be readily assembled in the solid-phase from inexpensive and commercially available materials.^11^ However, its *N* → *S* shift is usually very slow at neutral pH,^12^ preventing its direct use in NCL. We focused our efforts on addressing this challenge by taking inspiration from the mechanism of *in vivo* protein splicing promoted by class 1 inteins. The first step of this process consists of a self-catalyzed *N* → *S* acyl shift of an amine into a thioester,^8^ where a highly conserved histidine residue plays a pivotal role. This *N*-protonated His is believed to catalyze C–N bond scission through polarization and then protonation of the nitrogen leaving group (Fig. 2A).^13^ We reasoned that the *N*-alkyl group of cysteine could be functionalized by an appropriate intramolecular proton donor able to mimic the catalytic histidine side chain, resulting in a much faster *N* → *S* shift at neutral pH. Accordingly, we designed an *N*-(2-hydroxybenzyl)cysteine device (Fig. 2B) leading to a hypothetical thermodynamically favored six-membered transition state for intramolecular *N*-protonation. The key to this design is a phenol group with a p*K*_a_ close to neutral, as this would result in a low energy cost for the intramolecular proton transfer at pH 7.

We expected an additional advantage from the 2-hydroxybenzyl group: acceleration of *N*-acylation *via O*-acylation followed by the intramolecular *O* → *N* shift (Fig. 2C), as in the *N*-acylation of 2-hydroxy-5-methoxybenzyl (Hmb) protected amines.^14^ This could overcome a common drawback encountered with most *N* → *S* acyl shift-based thioesterification devices developed so far, the difficult *N*-acylation of a secondary amine,7g,h,^15^ to introduce the first residue of the peptide sequence.

### Optimization of the hydroxybenzyl group

Three different *N*-2-hydroxybenzyl groups were introduced on a solid supported cysteine derivative through an automated reductive amination protocol (compounds **2a–c**) and were compared with a reference *N*-ethyl cysteine device (**2d**). For this preliminary study, a reporter peptide (LYRAG-NH_2_) was introduced C-terminal to cysteine to facilitate the analysis using LC-MS after TFA-mediated deprotection and cleavage from the resin. Excellent results were obtained with the 2-hydroxy-5-nitrobenzyl (Hnb) group,^16^ giving quantitative *N*-acylation yields for a Gly residue under mild amide coupling conditions (HBTU/HOBt) (Table 1, entry 5), while the ethyl-substituted compound (Table 1, entry 7) was unreactive. Hmb or unsubstituted 2-hydroxybenzyl groups (Table 1, entries 1 and 3) showed only moderate assistance. These results are in line with the literature:^16^ Alewood and collaborators already observed the much superior acyl transfer efficiencies of 2,6- and 2,5-Hnb compared to Hmb. Moreover, the p*K*_a_ of the hydroxyl group of Hnb is close to 7 (∼6.5, see ESI, Fig. S11†), in line with our requirements discussed above for efficient biomimetic catalysis. We accordingly selected *N*-Hnb-Cys as our preferred scaffold.

**Table 1:** Screening of different *N*-(2-hydroxybenzyl) groups for their ability to enhance the *N*-acylation yield of a solid-supported *S*-trityl-protected *N*-substituted cysteine. PS: polystyrene

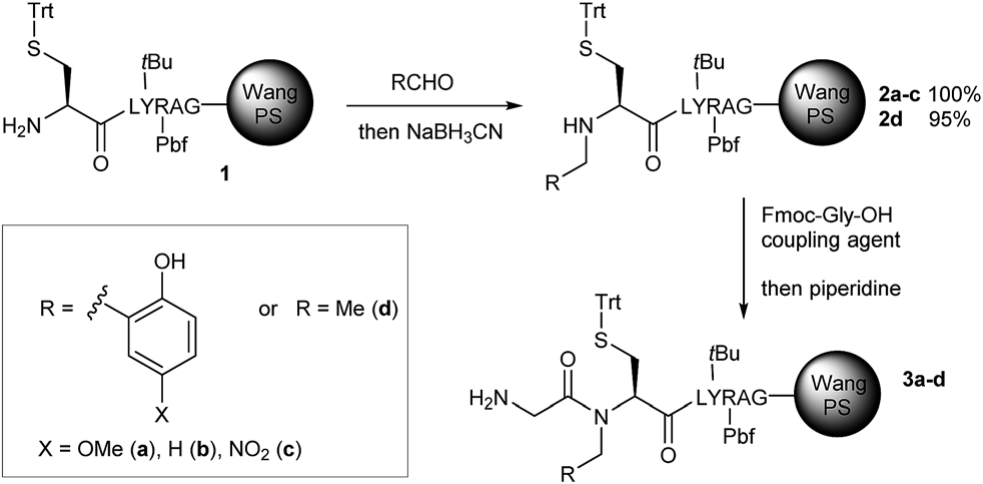				
Entry	Resin	Coupling agent^*a*^	Product	Yield^*b*^ [%]
1	**2a**	HBTU/HOBt	**3a**	<5^*c*^
2	**2a**	HATU	**3a**	45^*c*^
3	**2b**	HBTU/HOBt	**3b**	11
4	**2b**	HATU	**3b**	12
5	**2c**	HBTU/HOBt	**3c**	>99
6	**2c**	HATU	**3c**	>99
7	**2d**	HBTU/HOBt	**3d**	<1
8	**2d**	HATU	**3d**	12
9	**2d**	PyBrop	**3d**	1
10	**2d**	HATU/DMAP^*d*^	**3d**	13

^*a*^All reactions were conducted for 2 h, in DMF, using 10 equiv. Fmoc-Xaa-OH, 9.5 equiv. coupling agent and 20 equiv. iPr_2_NEt.

^*b*^Yields determined after TFA-mediated cleavage of the peptide resin using HPLC analysis of the amine-to-amide conversion.

^*c*^Estimated yields due to closely eluting HPLC peaks.

^*d*^0.1 equiv. DMAP.

### Optimization of the thiol protecting group of cysteine and linkage to the resin

When exploring the scope of peptides bearing our *N*-Hnb-Cys thioesterification device, we observed an instability upon purification and storage due to a premature spontaneous *N* → *S* shift. Fortunately, the protection of the cysteine thiol as an S-S*t*Bu disulfide, stable to Fmoc-SPPS and readily removed under NCL conditions,^17^ resolved this problem (see ESI, pS24–S29†).^18^ As the use of a reporter peptide was no longer needed at this point, we looked at introducing Cys(S*t*Bu) as either the α-acid or the amide, using a Wang-type or a Rink's amide linker, respectively. Disappointingly, in the former case we were confronted with Cys epimerization^19^ and β-elimination, followed by the addition of piperidine.^20^ In the latter case, we observed a TFA-catalyzed hydrolysis^21^ reaction, leading to a mixture of amide-(**7**) and acid-terminated (**8**) compounds (see ESI pS27–S29† for details). Simple incorporation of a Gly spacer between the Rink's linker and the device solved these problems. Preliminary evaluation of NCL reactions with the model cysteinyl peptide **6** showed that this additional residue (compound **9**) did not affect the NCL kinetics compared to cysteine α-amide **7**, the reaction being even slightly faster than for the α-acid **8** (ESI, Fig. S57†). As expected, cysteine deprotection was very fast, showing the complete removal of the S*t*Bu group within minutes.

### Study of the *N*-acylation of the device

*N*-Acylation of the optimized device was then examined for the twenty proteogenic amino acids, using a standard SPPS protocol (Table 2). In most cases the coupling yields were good to excellent. No significant epimerization was observed, except for Fmoc-Cys(Trt) (7%), which is well known for its propensity for racemization.^22^ Six residues showed modest *N*-acylation yields (<50%). In these difficult cases, we looked at further optimizing the coupling, avoiding the use of sensitive or costly reagents, and keeping in mind an easy-to-automate procedure. Three successive couplings of Fmoc-Ser(*t*Bu) resulted in an excellent yield (85%, entry 16). The five-fold coupling of the more demanding Fmoc-Val led to a good 65% yield (entry 21). Alternatively, single coupling under microwave heating at 70 °C led to an excellent 92% yield for Fmoc-Val (entry 22) and 78% for Fmoc-Ile (entry 24). Importantly, the unreacted secondary amine could be quantitatively capped by acetylation, giving a byproduct that can be readily removed during the peptide precipitation step following TFA-based cleavage from the resin.^23^

**Table 2:** Acylation of the resin **4** with the 20 different protected proteogenic amino acids under automated coupling conditions. TG: Tentagel

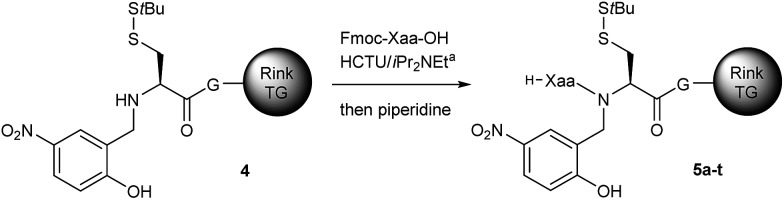				
Entry	Xaa	Product	Nb of couplings^*b*^	Yield^*c*^ [%]
1	Gly	**5a**	1	97
2	Asp(O*t*Bu)	**5b**	1	94
3	Ala	**5c**	1	90
4	Glu(O*t*Bu)	**5d**	1	89^*d*^
5	Cys(Trt)	**5e**	1	86^*e*^
6	Met	**5f**	1	85
7	Asn(Trt)	**5g**	1	80^*f*^
8	His(Trt)	**5h**	1	77
9	Phe	**5i**	1	77
10	Gln(Trt)	**5j**	1	72
11	Leu	**5k**	1	71
12	Trp(Boc)	**5l**	1	69
13	Arg(Pbf)	**5m**	1	67
14	Tyr(*t*Bu)	**5n**	1	58
15	Ser(*t*Bu)	**5o**	1	49
16	Ser(*t*Bu)	**5o**	3	85
17	Lys(Boc)	**5p**	1	45
18	Thr(*t*Bu)	**5q**	1	19
19	Pro	**5r**	1	19
20	Val	**5s**	1	16
21	Val	**5s**	5	65
22	Val	**5s**	1	92^*g*^
23	Ile	**5t**	1	12
24	Ile	**5t**	1	78^*g*^

^*a*^Reactions were conducted at a 0.025 mmol scale using a Prelude synthesizer at RT for 30 min in NMP/DMF 5 : 1, using 10 equiv. Fmoc-Xaa-OH, 9.5 equiv. HCTU and 20 equiv. iPr_2_NEt.

^*b*^When indicated, the coupling step was repeated under the same conditions.

^*c*^Yields determined after TFA-mediated cleavage of the peptidyl resin and subsequent HPLC analysis of the amine-to-amide conversion.

^*d*^Concomitant formation of pyroglutamate during TFA cleavage.

^*e*^93 : 7 mixture of l-Cys/d-Cys.

^*f*^Incomplete deprotection of the Trt group.

^*g*^Coupling at 70 °C.

### Mechanistic insights

**Fig. 3:**
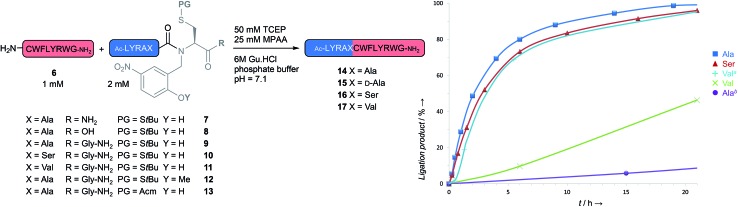
NCL of the model crypto-thioester peptides **7–11** bearing an *N*-Hnb-cysteine device, and an analog (**12**) with the hydroxyl group masked as a methyl ether. ^*a*^ 300 mM MPAA, pH 6.6, 50 °C. ^*b*^ Hnb methyl ether (**12**).

Next, we wanted to assess the validity of our intein-inspired design hypothesis that should result in the self-catalysis of the *N* → *S* shift at neutral pH through intramolecular protonation. Here, we synthesized a model peptide equipped with a 2-methoxy-5-nitrobenzylcysteine device (**12**), designed to inhibit the acid–base properties of the phenol group while not being expected to lead to major changes in terms of steric hindrance compared to Hnb (peptide **9**). We were delighted to see that the hydroxyl group very efficiently catalyzes the *N* → *S* acyl shift: >50-fold rate enhancement of NCL was measured for **9** compared to **12** under the NCL conditions (Fig. 3), supporting our hypothesis.^24^

Moreover, the ligation of **9** at different pH values showed the *N* → *S* acyl shift/NCL process to be fastest at pH ∼ 6.5, and slightly slower at lower pH (ESI, Fig. S67†). This fits with the p*K*_a_ of the Hnb group (6.5), suggesting the need for a subtle balance between the phenol group protonation and the thiol group deprotonation (cysteine p*K*_a_ ∼ 8.5) in influencing the *N* → *S* shift. To eliminate a possible *N* → *O* shift mechanism, the thiol was protected with a stable acetamidomethyl (Acm) group (**13**). This NCL-unreactive compound confirms the crucial role of the SH group and provides further data to suggest an intramolecular *N* → *S* shift mechanism. Critically, this latter finding also highlights the potential of the *N*-Hnb-Cys thioester precursors for successive ligations in the N-terminus-to-C-terminus (N-to-C) direction. We are currently evaluating latent crypto-thioesters that could be activated by deprotection of a NCL-stable cysteine protecting group, as has been exploited for related *N*-methyl Cys,^11^,^25^ CPE,^26^ SEA^17^ and Sealide^27^ thioester precursors.

### Study of the NCL with model peptides

Next, we turned our attention to the effectiveness of our new thioesterification device in NCL reactions for a range of thioesters bearing different amino acids C-terminal to the *N*-Hnb-Cys device (Fig. 3). Note that we used relatively dilute conditions for the peptide reactants (∼1 mM) and low concentrations of the 4-mercaptophenylacetic acid catalyst (MPAA, 25 mM).^28^ Relatively fast NCL kinetics were observed using Ala (peptide **9**) and Ser (**10**) crypto-thioesters, as the reactions were complete after 24 h at 37 °C. For comparison with the NCL kinetics data available in the literature, we determined the apparent second order kinetic constants, with results in the 0.03–0.06 M^–1^ s^–1^ range, in line with the reported values for standard NCL with preformed thioesters.^29^ These data validate the rapid *N* → *S* shift promoted by our device. Ligation at valine (crypto-thioester **11**) was 10 times slower, as we had anticipated from the steric hindrance of its side chain.^30^ However, increasing the MPAA concentration to 300 mM, lowering the pH close to the optimum (6.6) and heating to 50 °C led to kinetics comparable to Ala and Ser crypto-thioesters. The latter result suggests that the method could be applied in routine NCL even for peptide sequences known to be kinetically demanding.

Potential epimerization during the *N* → *S*-shift/NCL process was rigorously examined by synthesizing a d-Ala-containing product (**15**) as an HPLC standard. Only trace epimerization (<0.4%) was detected in the crude NCL mixture starting from the l-Ala crypto-thioester **9**. We also examined the hydrolysis of the crypto-thioesters into the corresponding acids. All reactions were clean and did not show significant amounts of hydrolysis (<4% for **9** after 24 h and using excess crypto-thioester).

### Application of the methodology to two cysteine-rich peptides

Importantly, our new method was applied to the synthesis of two long naturally-occurring cysteine-rich peptide sequences, MT7 and Cg-BigDef1. Muscarinic toxin 7 (MT7), 65 residues, was isolated from green mamba venom.^31^ MT7 naturally forms a three-finger-fold stabilized by four disulfide bonds. Big defensin 1 (Cg-BigDef1) is a 93-residue host-defense peptide isolated from Japanese oysters and is composed of a hydrophobic N-terminal domain and a β-defensin-like cationic C-terminal domain containing six cysteine residues involved in three disulfide bridges.^32^

**Fig. 4:**
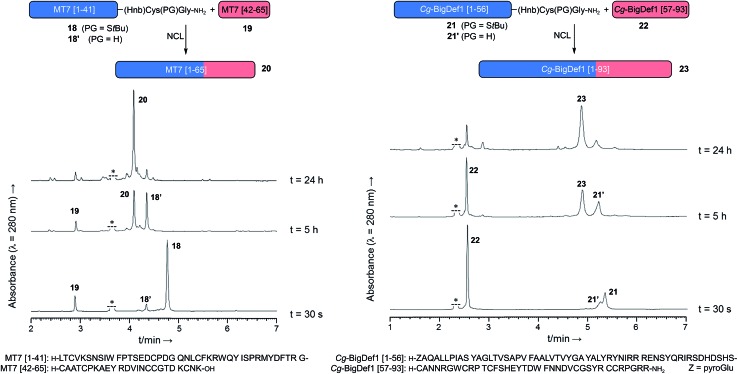
Application of the *N*-(2-hydroxy-5-nitrobenzyl)cysteine thioesterification device to the NCL-based syntheses of two long and demanding peptide sequences.

These two peptide sequences were chosen as challenging examples to demonstrate the applicability of our methodology, as their syntheses *via* the NCL of two segments require long thioesters, 41 and 56 amino acids, respectively. In addition, the N-terminal Cg-BigDef1 domain is a “difficult sequence”,^33^*i.e.* hard to synthesize using Fmoc-SPPS.^34^ Both of the crypto-thioesters (**18** and **21**) were synthesized and purified in good yields (13% and 7%, see ESI, pS86 and S93†). The two ligations were complete overnight at 1 and 2 mM concentrations, respectively, and showed clean HPLC profiles (Fig. 4). Finally, pure reduced forms of MT7 (**20**) and Cg-BigDef1 (**23**) were isolated after HPLC purification in 14% and 18% yields, respectively^35^ (ESI, Fig. S85 and S93†), demonstrating the utility of the *N*-Hnb-Cys method.

## Conclusions

In conclusion, we introduced a novel C-terminal thioesterification device, *N*-Hnb-Cys(S*t*Bu), that can rapidly rearrange into a thioester at neutral pH, allowing its use in NCL even under demanding conditions. Fast kinetics likely result from an internal catalysis mechanism provided by a well-positioned phenol moiety arising from a bio-inspired intein-like design. Synthesis from inexpensive commercially available materials and further peptide elongation are straightforward, and can be fully automated on a peptide synthesizer without requiring a post-synthetic step, thus constituting a major advance in the field. Applicability of the strategy was demonstrated by the synthesis of two long naturally occurring cysteine-rich peptide sequences. Further work is ongoing in our laboratory for its application to other synthetically demanding targets.

## Supplementary Material

Supplementary information
