# Supplementary material for: A straightforward method for automated Fmoc-based synthesis of bio-inspired peptide crypto-thioesters
Source: Chem Sci. 2015 Sep 23;7(1):339–45. doi: 10.1039/c5sc02630j (PMC5952550; doi:10.1039/c5sc02630j)
Supplement: Supplementary file 1 [file SC-007-C5SC02630J-s001.pdf]

Electronic Supplementary Material (ESI) for Chemical Science.
This journal is © The Royal Society of Chemistry 2015

A straightforward method for automated Fmoc-based synthesis of bio-inspired peptide crypto-thioesters

Victor P. Terrier, Hélène Adihou, Mathieu Arnould, Agnès F. Delmas, Vincent Aucagne

Centre de Biophysique Moléculaire, CNRS UPR 4301, Rue Charles Sadron 45071 Orléans cedex 2, France.

Supporting Information

Table of contents:

1) General information S2
2) General procedures for solid phase peptide synthesis S3
3) Optimization of the benzyl group to maximize the *N*-acylation yield S4
  a- Synthesis of cysteinyl peptide resin **1** S4
  b- Synthesis of peptide resins **2a-d** S5
  c- Study on the solid-supported *N*-acylation of **2a-d** S9
4) Evaluation of the phenol pKa of an *N*-acyl-*N*-(Hnb)Cys compound **S3** S12
5) Characterization of a model *N*-acyl-*N*-(Hnb)Cys compound **S5** S14
  a- NMR of **S5** S14
  b- Molar extinction coefficient of the Hnb group of **S5** S24
6) Optimization of the cysteine thiol protecting group S24
  a- Synthesis and stability of (*S*-Trt) model peptide **S7** S24
  b- Synthesis and stability of (*S*-S*t*Bu) model peptide **7** S28
7) Optimization of the spacer between the cysteine and the resin S30
  a- Direct attachment to Rink linker – amide hydrolysis co-product S30
  b- Direct attachment to PHB Tentagel resin (Wang type linker) S31
  c- Introduction of a Gly residue as a spacer S32
8) *N*-Acylation with the 20 different proteogenic amino acids S34
9) Kinetics studies of the NCL with model Ac-LYRAA(Hnb)C peptides S57
  a- Synthesis of model cysteinyl peptide **6** S57
  b- Influence of the nature of the cysteine C-terminus S59
  c- Study of the hydrolysis of the thioester precursor **9** and epimerization S62
  d- Influence of the buffer S64
  e- Influence of masking phenol or thiol groups S65
  f- Influence of the pH S71
10) Influence of the C-terminal aa: NCL with model Ac-LYRAX(Hnb)C peptides S73
  a- Synthesis of peptide Ac-LYRAS-(Hnb)C(S*t*Bu)G-NH_2 **10** S73
  b- NCL with peptide Ac-LYRAS-(Hnb)C(S*t*Bu)G-NH_2 **10** S75
  c- Synthesis of peptide Ac-LYRAV-(Hnb)C(S*t*Bu)G-NH_2 **11** S76
  d- NCL with peptide Ac-LYRAV-(Hnb)C(S*t*Bu)G-NH_2 **11** S78
  e- NCL with peptide **11** under optimized conditions S78
  f- Determination of apparent second order kinetic constants S79
11) Application to MT7 S83

a- Synthesis of cysteinyl peptide **19** S83
b- Synthesis of crypto-thioester peptide **18** S86
c- NCL S89
12) Application to *Cg*-Bigdef1 S91
a- Synthesis of cysteinyl peptide **22** S91
b- Synthesis of crypto-thioester peptide **21** S93
c- NCL S96

1- General information

All reagents and solvents were used without further purification. Protected amino acids, Fmoc-Gly-Wang resin, Fmoc-Rink polystyrene resin, Rink linker, HBTU and HCTU were purchased from Merck Biosciences (Nottingham, UK). Aminomethyl TentaGel R resin and pre-loaded Fmoc-Cys(StBu)-PHB (Wang type) Tentagel resin were purchased from Rapp polymers (Tuebingen, Germany). Fmoc-K(Boc)-Mppa-OH was purchased from Polypeptide laboratories (Strasbour, France). Peptide synthesis grade DMF and HATU were obtained from Applied Biosystems (Courtaboeuf, France). Ultrapure water was obtained using a Milli-Q water system from Millipore (Molsheim, France). All other chemicals were from Sigma Aldrich (St-Quentin-Fallavier, France) and solvents from SDS-Carlo Erba (Val de Reuil, France).

^{1}H and ^{13}C NMR spectra were recorded on a Bruker AVANCE III 600 instrument, at a constant temperature of 25°C. Chemical shifts are reported in parts per million from low to high field and referenced to tetramethylsilane (TMS). Coupling constants (*J*) are reported in hertz (Hz). Standard abbreviations indicating multiplicity were used as follows: s = singlet, d = doublet, dd = doublet of doublets, t = triplet, m = multiplet, b= broad signal.

High resolution ESI-MS analyses were performed on a maXis ultra-high-resolution Q-TOF mass spectrometer (Bruker Daltonics, Bremen, Germany), using the positive mode. MALDI-TOF analyses were performed on an Ultraflex instrument (Bruker Daltonics, Bremen, Germany) equipped with a 337-nm nitrogen laser and a gridless delayed extraction ion source. The sample was co-crystallized with a solution of α-cyano-4-hydroxy-cinnamic acid (HCCA) as a matrix. The reported *m/z* values correspond to the monoisotopic ions if not specified otherwise. Peptides incorporating an *N*-(2-hydroxy-5-nitrobenzyl) group displayed a typical MALDI fragmentation pattern, consisting in -16 Da and -34 Da minor peaks in addition to the $[MH]^+$ peak.

HPLC analyses and semi-preparative purifications were carried out on a LaChrom Elite system equipped with a Hitachi L-2130 pump, a Hitachi L-2455 diode array detector and a Hitachi L-2200 autosampler. Nucleosil C18 (300 Å, 5 µm, 250 × 4.6 mm, 1 mL/min flow rate) or Chromolith HighResolution RP-18e (150 Å, 10 × 4.6 mm, 3 mL/min flow rate) columns were used for analysis and Nucleosil C18 (300 Å, 5 µm, 250 × 10 mm, 3 mL/min flow rate) for purification. Solvents A and B are 0.1 % TFA in

H_2O and 0.1 % TFA in MeCN, respectively. Each gradient was followed by a washing step (95% B/A over 0.5 min for the HR Chromolith; over 1 min for the Nucleosil C18 column) to identify eventual co-products not eluted during the gradient.

LC-MS analyses were carried out on a Ultimate® 3000 RSLC HPLC system (Dionex, Germering, Germany), coupled with the maXis mass spectrometer and fitted with a Zorbax 300 SB-C18 RRHD (300 Å, 1.8 µm, 100 × 2.1 mm, 0.3 mL/min flow rate, 40°C) column. Solvents A and B were 0.1 % formic acid in H_2O and 0.08 % formic acid in MeCN, respectively. Gradient: 3 % B/A for 1 min, then 3 to 50 % B/A over 19 min.

2- General procedures for solid phase peptide synthesis

Fmoc-based solid phase peptide syntheses (SPPS) were carried out on either a Prelude synthesizer from Protein Technologies or a 433A synthesizer from Applied Biosystems. Microwave-assisted Fmoc-SPPS syntheses were carried out on an Initiator+ SP Wave synthesizer from Biotage.

The side-chain protecting groups used were Arg(Pbf), Asn(Trt), Asp(O*t*Bu), Cys(Acm), Cys(Trt), Cys(S*t*Bu), Glu(O*t*Bu), Gln(Trt), His(Trt), Lys(Boc), Ser(*t*Bu), Thr(*t*Bu), Trp(Boc) and Tyr(*t*Bu).

Syntheses starting from aminomethyl TentaGel R resin were performed on the Prelude synthesizer, on a 0.025 mmol per reactor scale. Protected amino acids (0.25 mmol, 10 equiv.) were coupled using HCTU (98 mg, 0.238 mmol, 9.5 equiv.) and *i*Pr_2NEt (87 µL, 0.5 mmol, 20 equiv.) in NMP (3 mL) for 30 min. Capping of eventual unreacted amine groups was achieved by treatment with acetic anhydride (143 µL, 1.51 mmol, 60 equiv.), *i*Pr_2NEt (68 µL, 0.39 mmol, 15.5 equiv.) and HOBt (6 mg, 0.044 mmol, 1.8 equiv.) in NMP (3 mL) for 7 min. Fmoc group was deprotected by three successive treatments with 20 % piperidine in NMP (3 mL) for 3 min. In the cases where the coupling yield of an amino acid on the Hnb device is low, a washing step of the peptide-resin using a solution of hydroxylamine hydrochloride (0.3 M) and imidazole (0.225 M) in a NMP / CH_2Cl_2 mixture[1] (5:1, 20 min, 3 mL, ×3) is performed before the capping step.

Syntheses starting from Fmoc-Gly-Wang or Rink polystyrene resins were performed on the 433A synthesizer, using the 0.1 mmol scale Fastmoc program purchased from the manufacturer, with a single coupling with HCTU followed by capping.

The crude peptide was deprotected and cleaved from the resin through a treatment with TFA/H_2O/*i*Pr_3SiH/phenol, 88/5/2/5 for 2 h, and the peptide was precipitated by dilution into an ice-cold 1:1 diethyl ether/petroleum ether mixture, recovered by centrifugation and washed twice with diethyl ether.

[1] Diaz-Mochon J. J., Bialy L., Bradley M., *Org. Lett.,* 2004, **6**, 7, 1127-1129

3- Optimization of the benzyl group to maximize the *N*-acylation yield

3a- Synthesis of cysteinyl peptide resin **1**

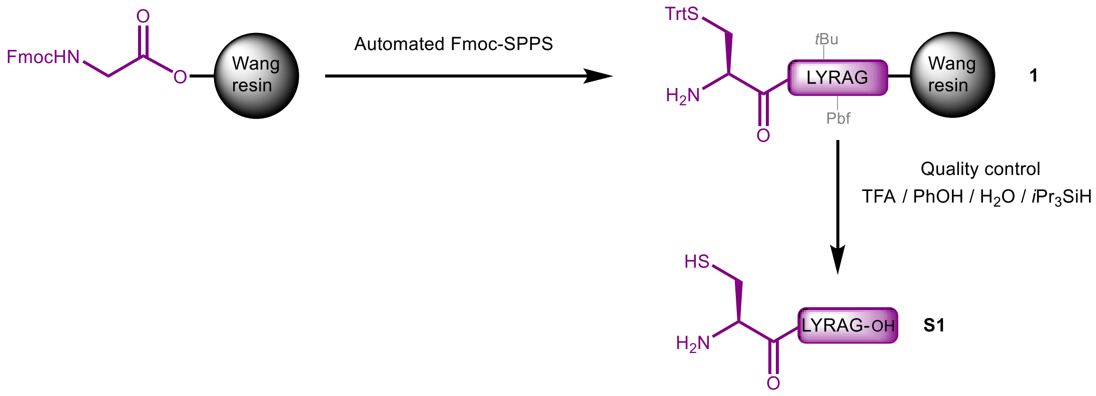

Supplementary scheme S1: Synthesis of peptide resin **1**.

Peptide resin **1** was obtained through automated SPPS (protocol p S3) starting from Fmoc-Gly-Wang resin (130 mg, 0.79 mmol/g, 0.1 mmol). An aliquot of the resin was cleaved (protocol p S3) in order to characterize the corresponding peptide **S1**.

S1:

MALDI-TOF MS (*m*/*z*): $[M+H]^+$ calcd for $C_{29}H_{47}N_9O_8S$: 682.3, found: 682.3.
HPLC: retention time: 14.41 min (Nucleosil, gradient: 5-50 % B/A over 30 min).

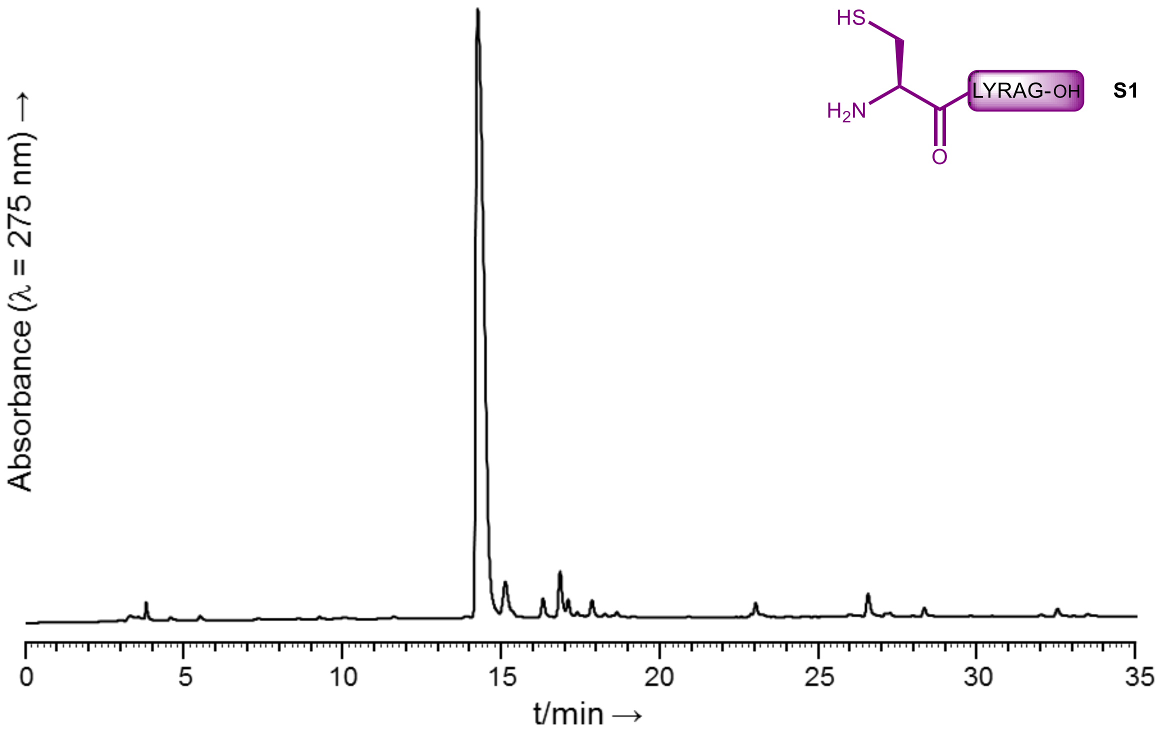

Supplementary figure S1: HPLC trace of crude **S1**.

3b- Synthesis of peptide resins **2a-d** by solid-supported *N*-alkylation

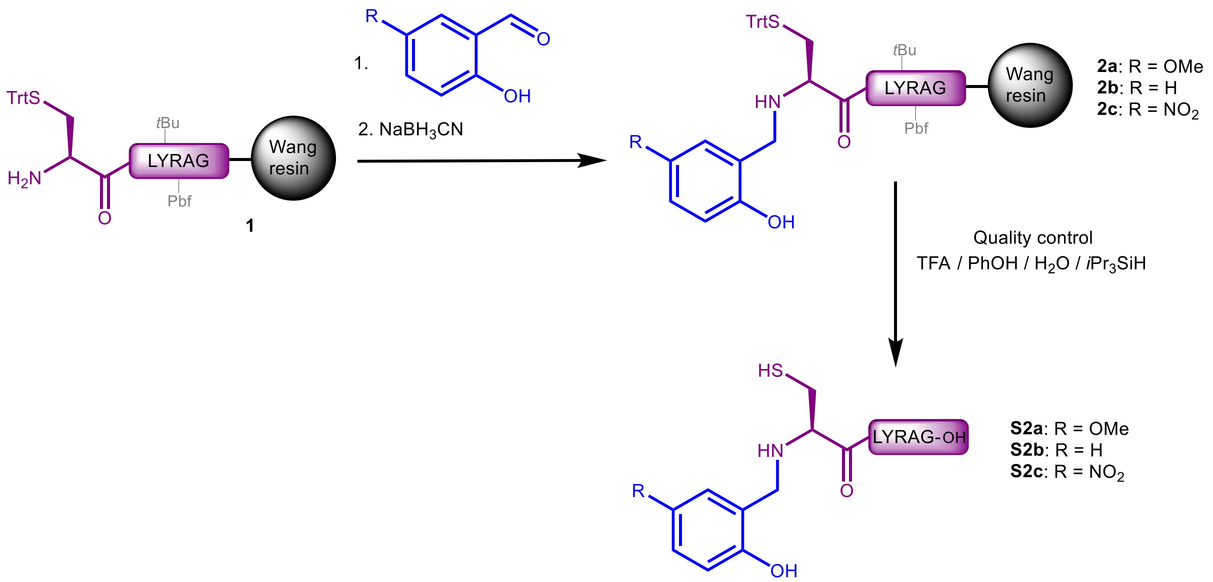

Supplementary scheme S2: Synthesis of *N*-benzyl peptide resins **2a-c** through solid-supported reductive amination.

Peptide resin **1** (10 µmol) was introduced into a polypropylene syringe fitted with a polypropylene frit and a PTFE stopcock, then swollen in a DMF/MeOH/AcOH (9:9:2) mixture for 5 min. The syringe was drained and the resin was washed with a DMF/MeOH (1:1) mixture. The appropriate aldehyde (100 µmol, 10 equiv.) dissolved in DMF/MeOH (0.4 mL) was added then the syringe was left for 45 min under gentle stirring. The syringe was drained off and the resin was washed with DMF/MeOH. Without delay, sodium cyanoborohydride (13 mg, 200 µmol, 20 equiv.) dissolved in DMF/MeOH/AcOH (0.4 mL) was added and the syringe was left for 30 min under gentle stirring. The syringe was drained and the resin was thoroughly washed with DMF/MeOH/AcOH, NMP, 5% *i*Pr_2NEt in NMP then NMP. An aliquot of each peptide resin **2a-c** was cleaved (protocol p S3) in order to characterize the corresponding peptides **S2a-c**.

S2a:

MALDI-TOF (*m/z*): $[MH]^+$ calcd for $C_{37}H_{55}N_9O_{10}S$: 818.4, found: 818.4.

HPLC: retention time: 19.59 min (Nucleosil, gradient: 5-50 % B/A over 30 min).

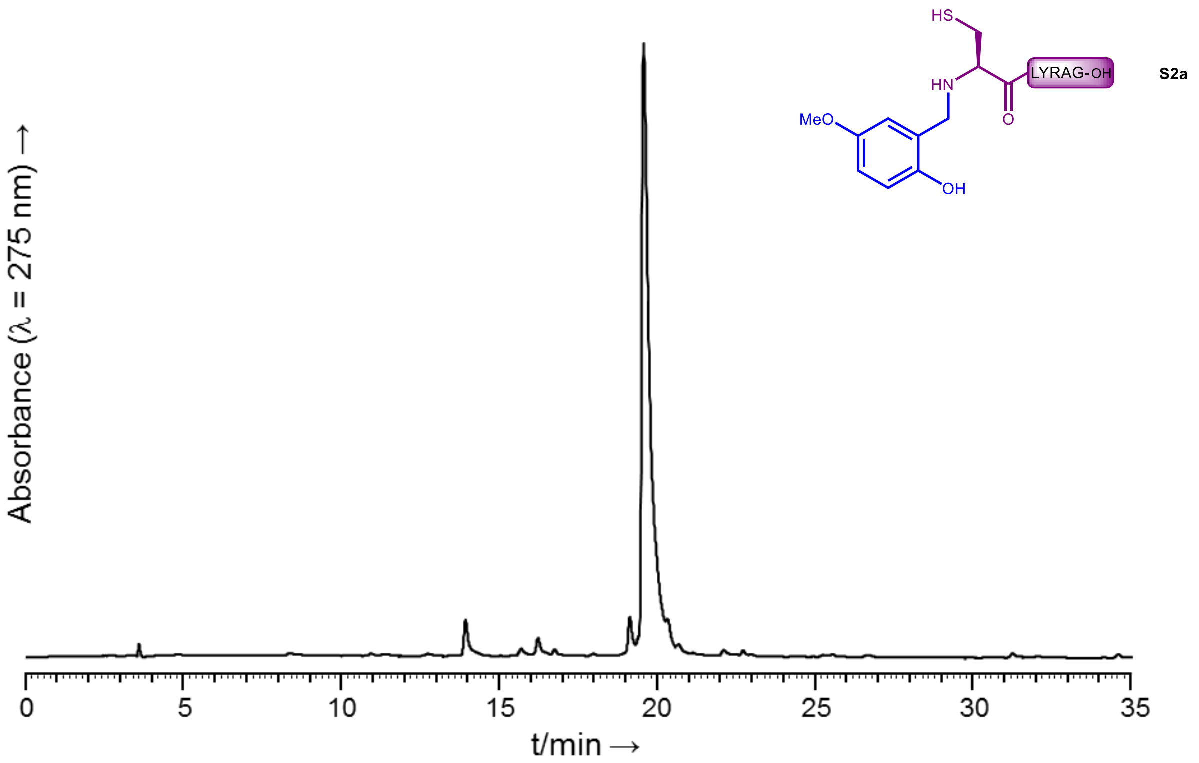

Supplementary figure S2: HPLC trace of crude **S2a**.

S2b:

MALDI-TOF (*m/z*): [MH]$^+$ calcd for $C_{36}H_{53}N_9O_9S$: 788.4, found: 788.3.
HPLC: retention time: 19.17 min (Nucleosil, gradient: 5-50 % B/A over 30 min).

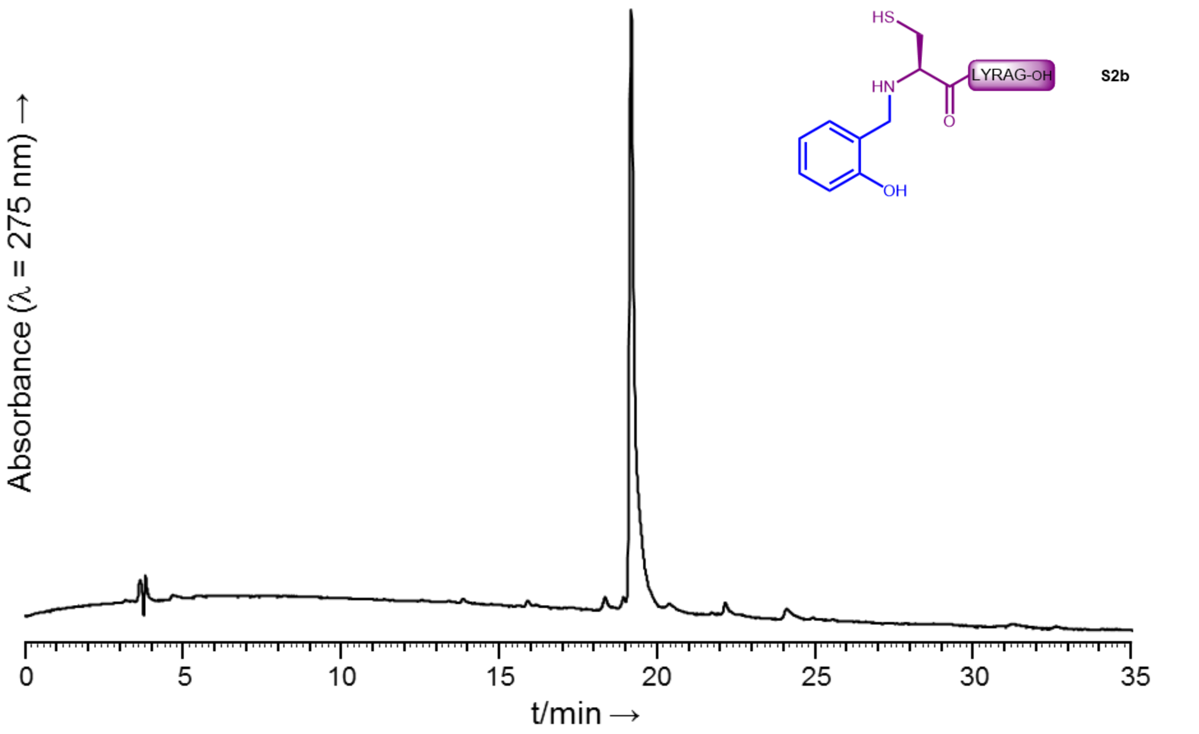

Supplementary figure S3: HPLC trace of crude **S2b**.

S2c:

MALDI-TOF (*m/z*): $[MH]^+$ calcd for $C_{36}H_{52}N_{10}O_{11}S$: 833.4, found: 833.3.

HPLC: retention time: 20.44 min (Nucleosil, gradient: 5-50 % B/A over 30 min).

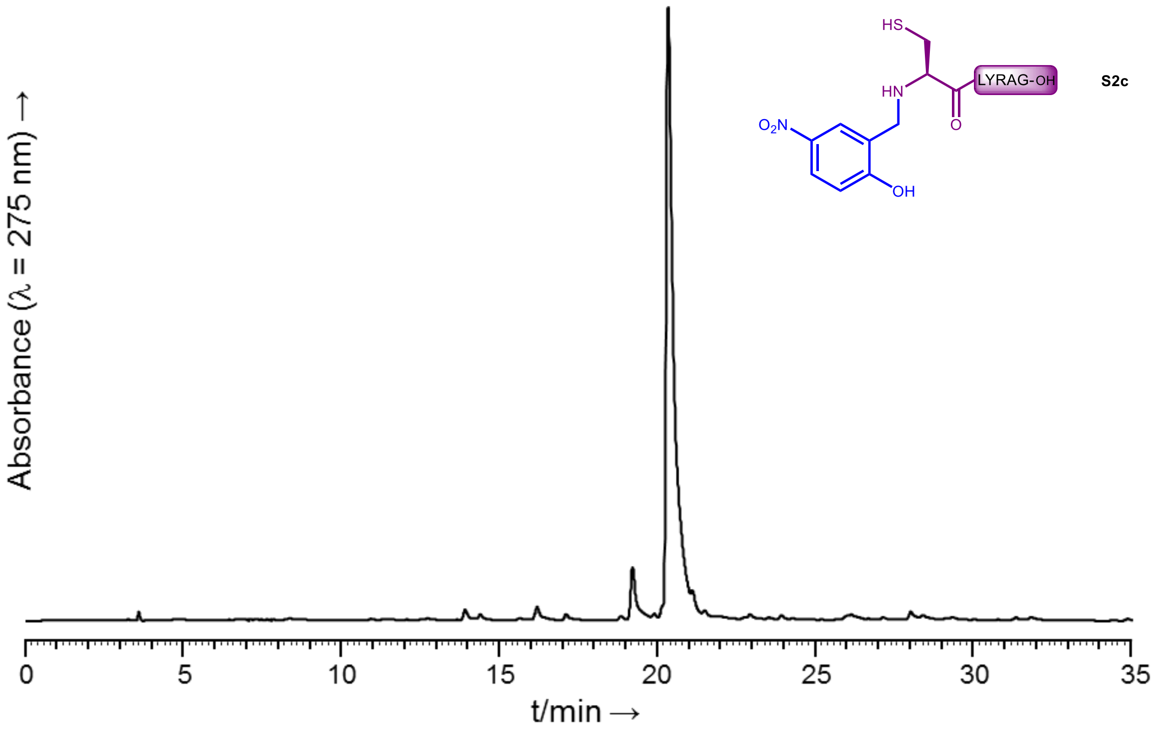

Supplementary figure S4: HPLC trace of crude **S2c**.

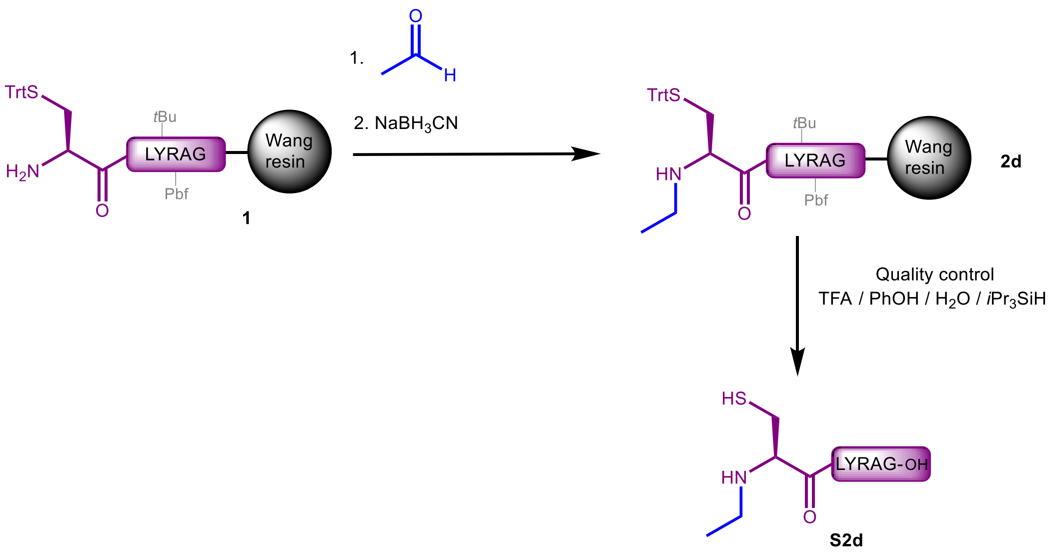

Supplementary scheme S3: Synthesis of *N*-ethyl peptide resin **2d** through solid-supported reductive amination.

Peptide resin **1** (10 µmol, 1 equiv.) was introduced into a polypropylene syringe fitted with a polypropylene frit and a PTFE stopcock and swollen in a mixture of DMF/MeOH/AcOH (9:9:2) for 5 min. The syringe was drained and the resin was washed with a mixture of DMF/MeOH (1:1). Acetaldehyde (100 µmol, 10 equiv.) dissolved in DMF/MeOH (0.4 mL) was added then the syringe was left for 5 min under gentle stirring. The syringe was drained and the resin was washed with DMF/MeOH. Without delay, sodium cyanoborohydride (13 mg, 200 µmol, 20 equiv.) dissolved in DMF/MeOH/AcOH (0.4 mL) was added and the syringe was left for 30 min under gentle stirring. The syringe was drained and the resin was thoroughly washed with DMF/MeOH/AcOH, NMP, 5% *i*Pr_2NEt in NMP then NMP. An aliquot of the resulting peptide resin was cleaved (protocol p S3) in order to characterize *N*-ethyl peptide **S2d**.

S2d:

MALDI-TOF (*m/z*): $[MH]^+$ calcd for $C_{31}H_{51}N_9O_8S$: 710.4, found 710.4.

HPLC: retention time: 15.36 min (Nucleosil, gradient: 5-50 % B/A over 30 min).

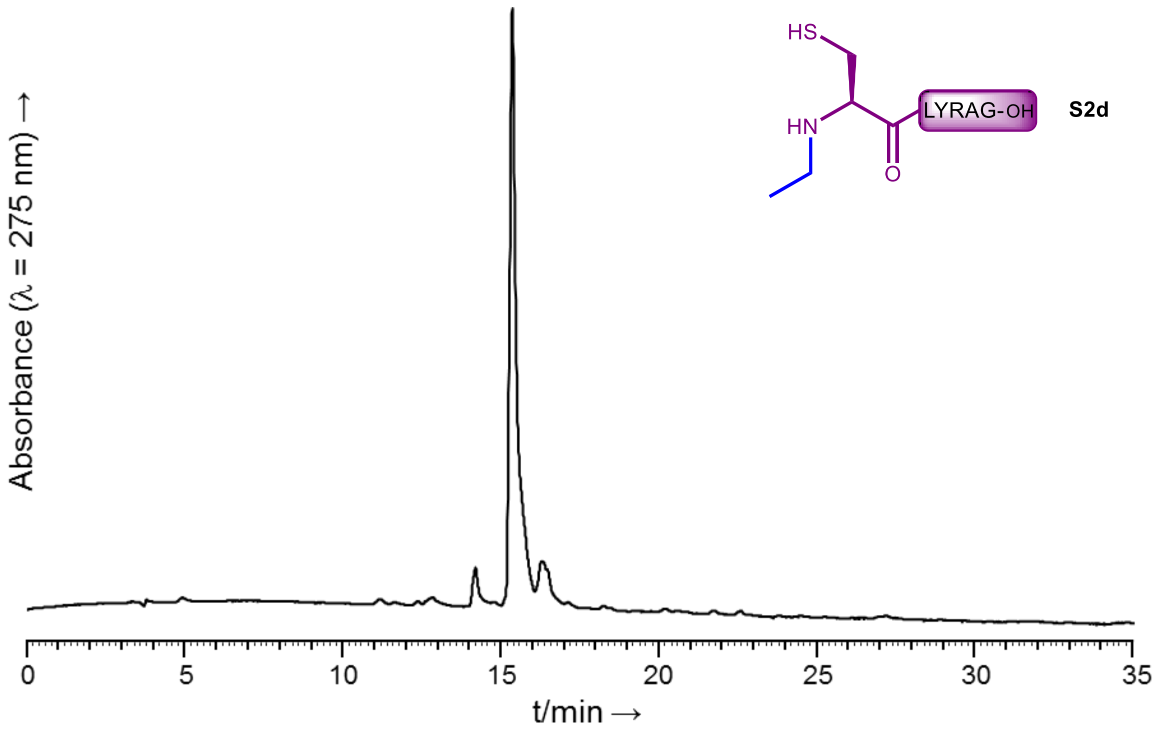

Supplementary figure S5: HPLC trace of crude **S2d**.

3c- Study on the solid-supported *N*-acylation of secondary amines **2a-d**

General procedure for solid-supported N-acylation

Peptide resin (**2a-d**) was introduced into a polypropylene syringe fitted with a polypropylene frit and a PTFE stopcock and swollen with NMP. Then, Fmoc-Gly-OH (10 equiv.), HBTU (9.5 equiv.) and HOBt (9.5 equiv.) were dissolved in NMP (0.1 M final concentration in amino acid) prior to addition of *i*Pr_2NEt (20 equiv.). The resulting solution was immediately added to the resin. The syringe was left for 2 h under gentle stirring, then the syringe was drained and the resin was washed with NMP. An aliquot resin was cleaved using the standard procedure (p S3) to determine the coupling yields and characterize the corresponding *N*-acylated peptides.

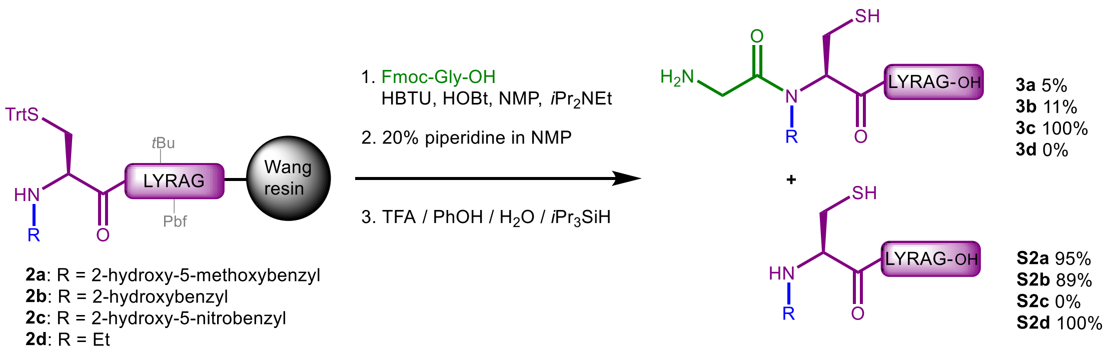

Supplementary scheme S4: Study on the influence of the *N*-alkyl group (R) of peptide resins **2a-d** on the *N*-acylation yield.

3a:

MALDI-TOF (*m/z*): $[MH]^+$ calcd for $C_{39}H_{58}N_{10}O_{11}S$: 875.4, found: 875.4.
HPLC: retention time: 17.24 min (Nucleosil, isocratic 17% B/A over 30 min at 50°C).

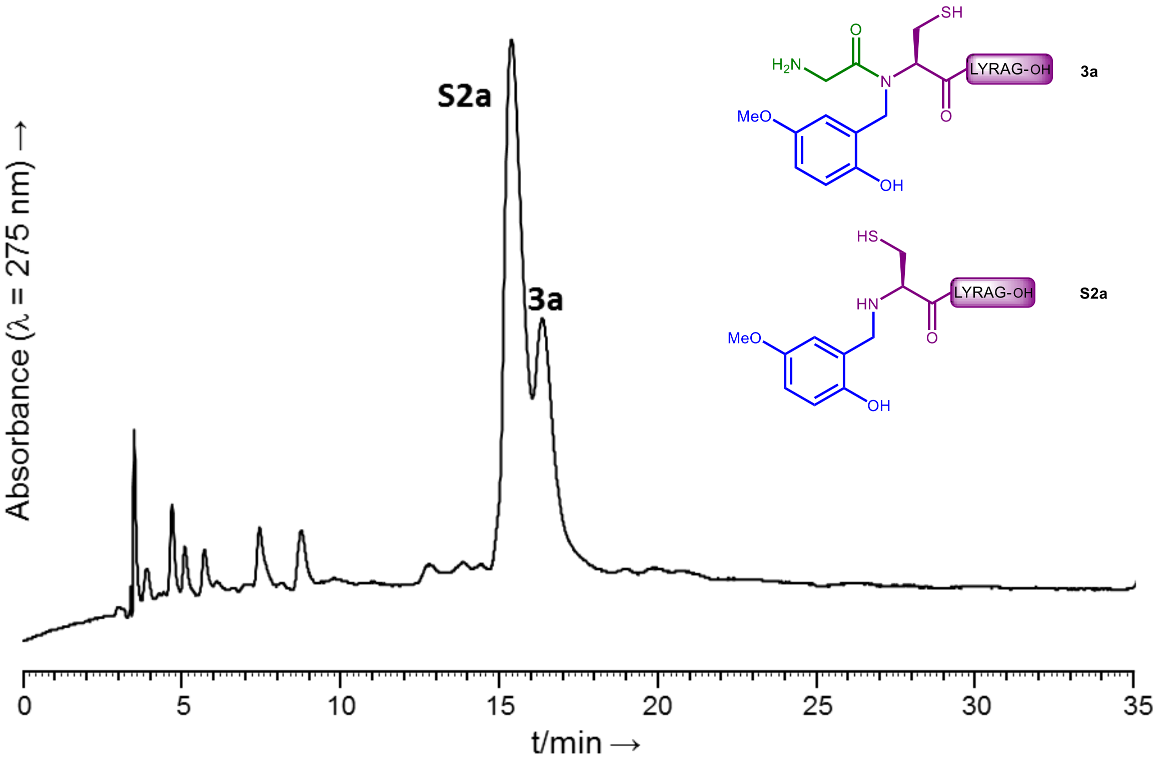

Supplementary figure S6: HPLC trace of crude **3a**.

3b:

MALDI-TOF (*m/z*): $[MH]^+$ calcd for $C_{38}H_{56}N_{10}O_{10}S$: 845.4, found 845.4.
HPLC: retention time: 19 min (Nucleosil, gradient: 5-50 % B/A over 30 min).

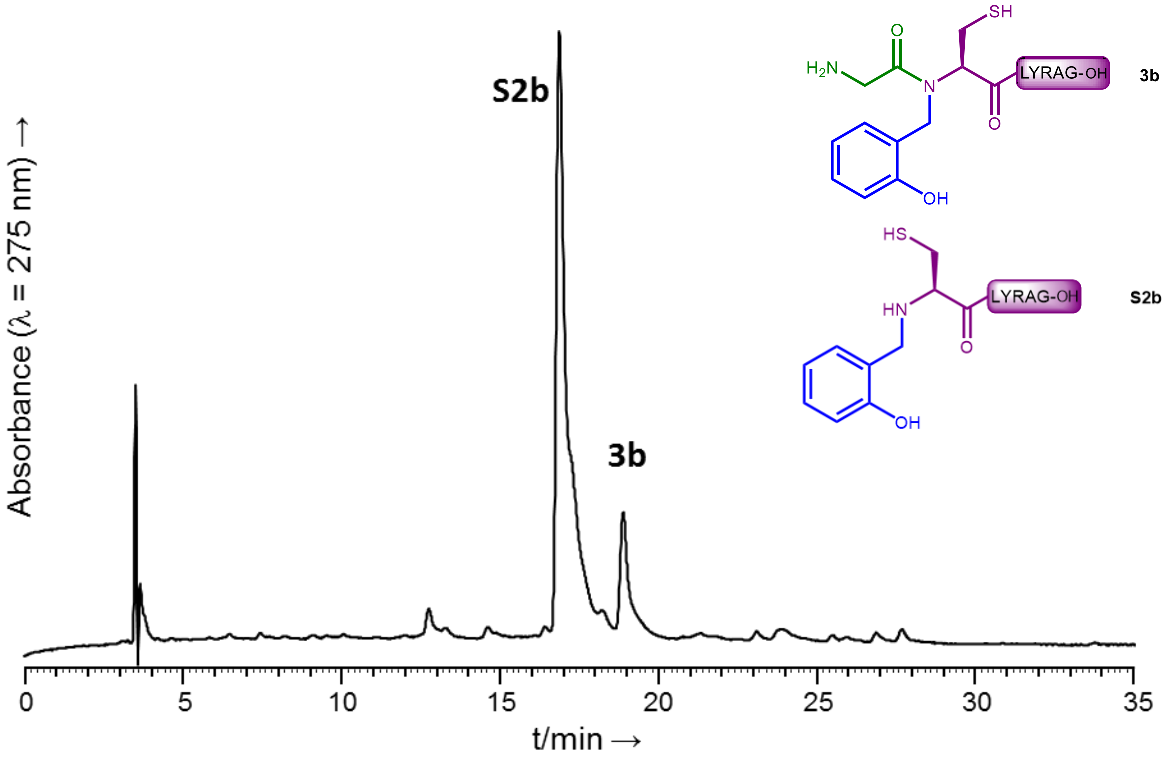

Supplementary figure S7: HPLC trace of crude **3b**.

3c:

MALDI-TOF (*m/z*): $[MH]^+$ calcd for $C_{38}H_{55}N_{11}O_{12}S$: 890.4, found: 890.4.

HPLC: retention time: 20.53 min (Nucleosil, gradient: 5-50 % B/A over 30 min).

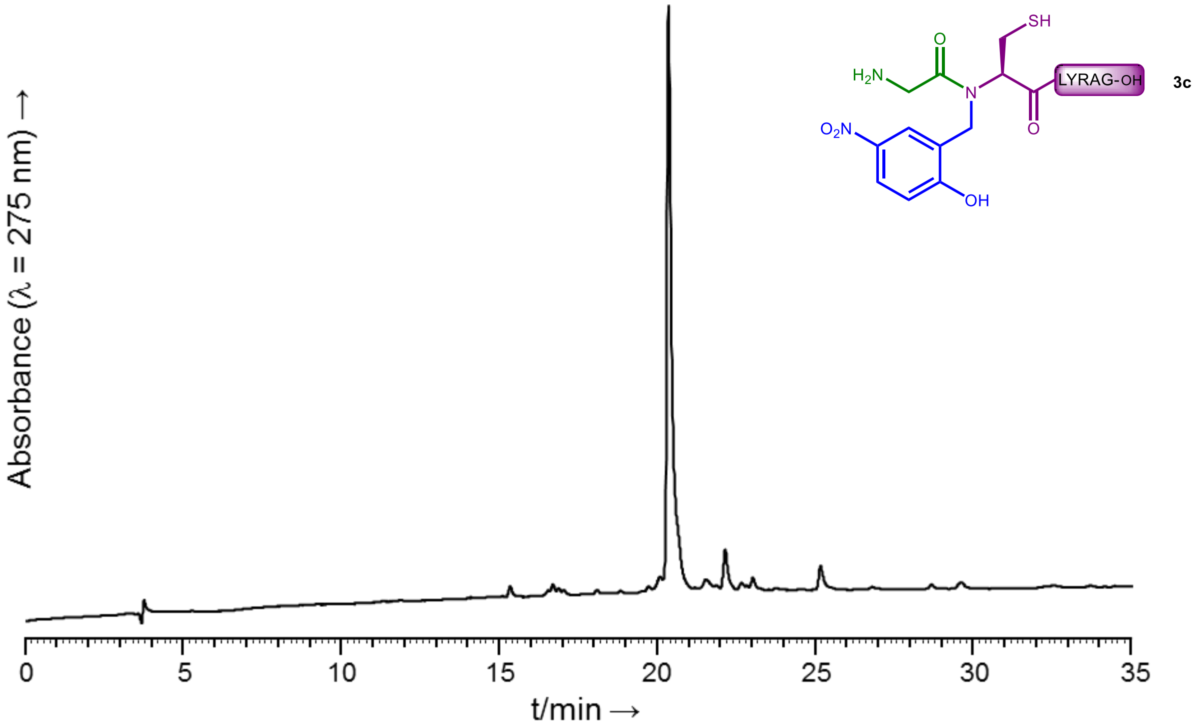

Supplementary figure S8: HPLC trace of crude **3c**.

N-acylation of peptide resin **2d** using HBTU/HOBt followed by deprotection of the Fmoc group then cleavage of the resin did not show any detectable amount of the expected *N*-acylated peptide **3d**. For characterization purpose, the *N*-acylation was performed using HATU as the coupling reagent, yielding to a small amount of **3d** together with the non-acylated peptide **S2d**.

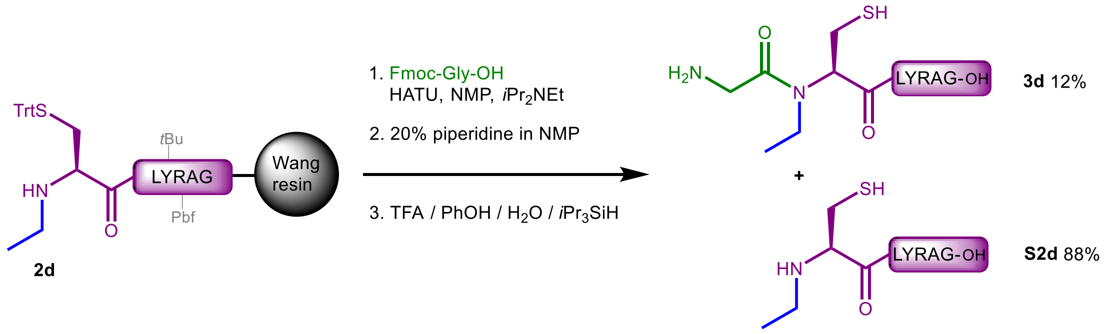

Supplementary scheme S5: *N*-acylation of peptide resin **2d** using HATU as the coupling reagent.

3d:

MALDI-TOF (*m/z*): $[MH]^+$ calcd for $C_{33}H_{54}N_{10}O_9S$: 767.4, found 767.4.
HPLC: retention time: 17.02 min (Nucleosil, gradient: 5-50 % B/A over 30 min).

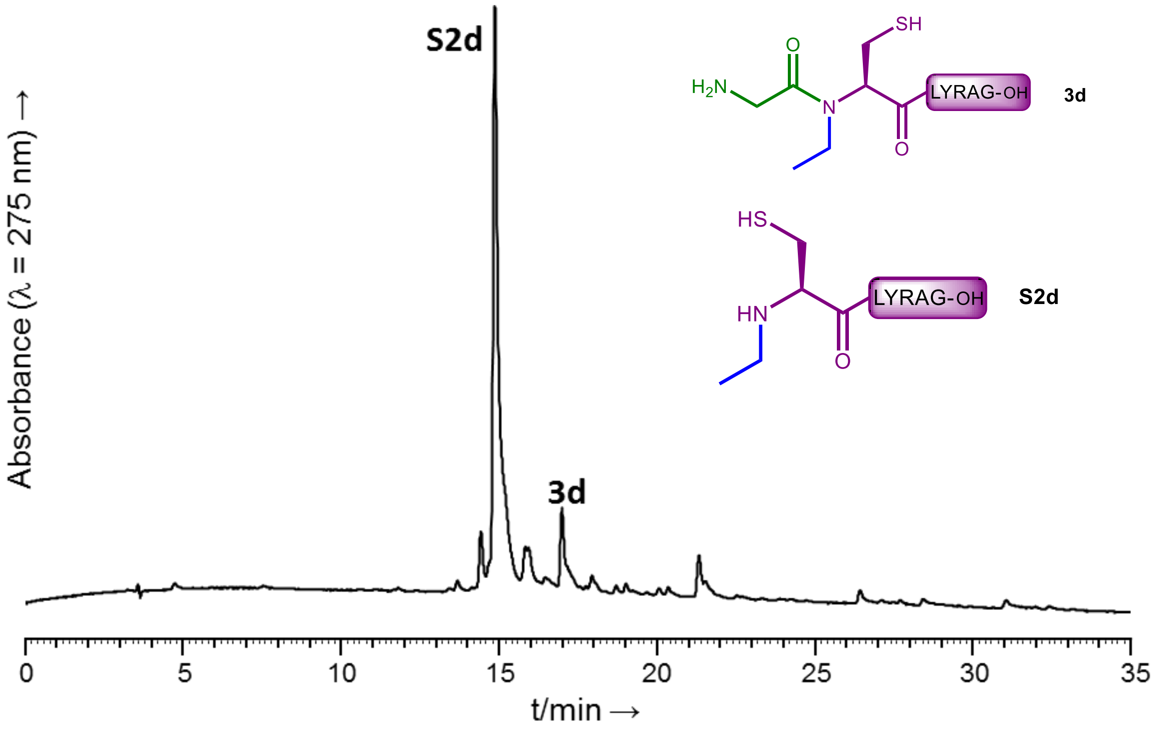

Supplementary figure S9: HPLC trace of crude **3d**.

4- Evaluation of the phenol pKa of an *N*-acyl-*N*-(Hnb)Cys compound

*Synthesis of S-alkylated compound **S3***

In order to prevent any *N*-*S* shift that would make uncertain the determination of the pKa of the phenol group, we synthesized a model dipeptide **S3** *S*-alkylated with an acetamidomethyl (Acm) protective group.

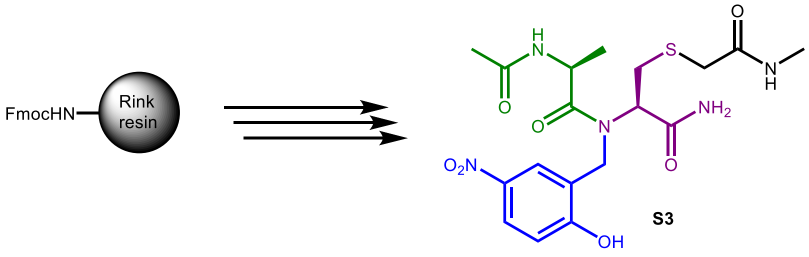

Supplementary scheme S6: Synthesis of the model dipeptide **S3**.

Fmoc-Rink polystyrene resin (182 mg, 0.55 mmol/g, 0.1 mmol) was introduced into a polypropylene syringe fitted with a polypropylene frit and a PTFE stopcock and swollen with NMP. The resin was treated with 20% piperidine in NMP (10 mL, 3 × 3 min) and washed with NMP. Then, Fmoc-Cys(Acm)-OH (414 mg, 1 mmol, 10 equiv.), HBTU (360 mg, 0.95 mmol, 9.5 equiv.) and HOBt (128 mg, 0.95 mmol, 9.5 equiv.)

were dissolved in NMP (10 mL) prior to addition of *i*Pr_2NEt (348 µL, 2 mmol, 20 equiv.). The resulting solution was immediately added to the resin. The syringe was left for 1 h under gentle stirring, then the syringe was drained and the resin was washed with NMP. Fmoc group was deprotected by three successive treatments with 20% piperidine in NMP (10 mL) for 3 min. The 2-hydroxy-5-nitrobenzyl linker was then introduced following the general procedure (p S5). Fmoc-Ala-OH (311 mg, 1 mmol, 10 equiv.) was coupled following the same coupling procedure as described above. Coupling was repeated once. The resin was then treated with 20% piperidine in NMP (10 mL, 3 × 3 min), washed with NMP, then treated with acetic anhydride (571 µL, 6 mmol, 60 equiv.), *i*Pr_2NEt (272 µL, 0.39 mmol, 15.5 equiv.) and HOBt (24 mg, 0.044 mmol, 1.8 equiv.) in NMP (12 mL) for 2 × 1 h. Peptide-resin was then treated with 20% piperidine in NMP (10 mL, 3 × 3 min), washed with NMP then CH_2Cl_2, and finally cleaved using a solution of TFA / H_2O (9:1) for 2 h. The solvents were removed under reduced pressure to give compound **S3**.

S3:

ESI-HRMS (*m*/*z*): $[MH]^+$ calcd for $C_{18}H_{26}N_5O_7S$: 456.1547, found: 456.1544.
HPLC: retention time: 12.35 min (Nucleosil, gradient: 5-95 % B/A over 30 min).

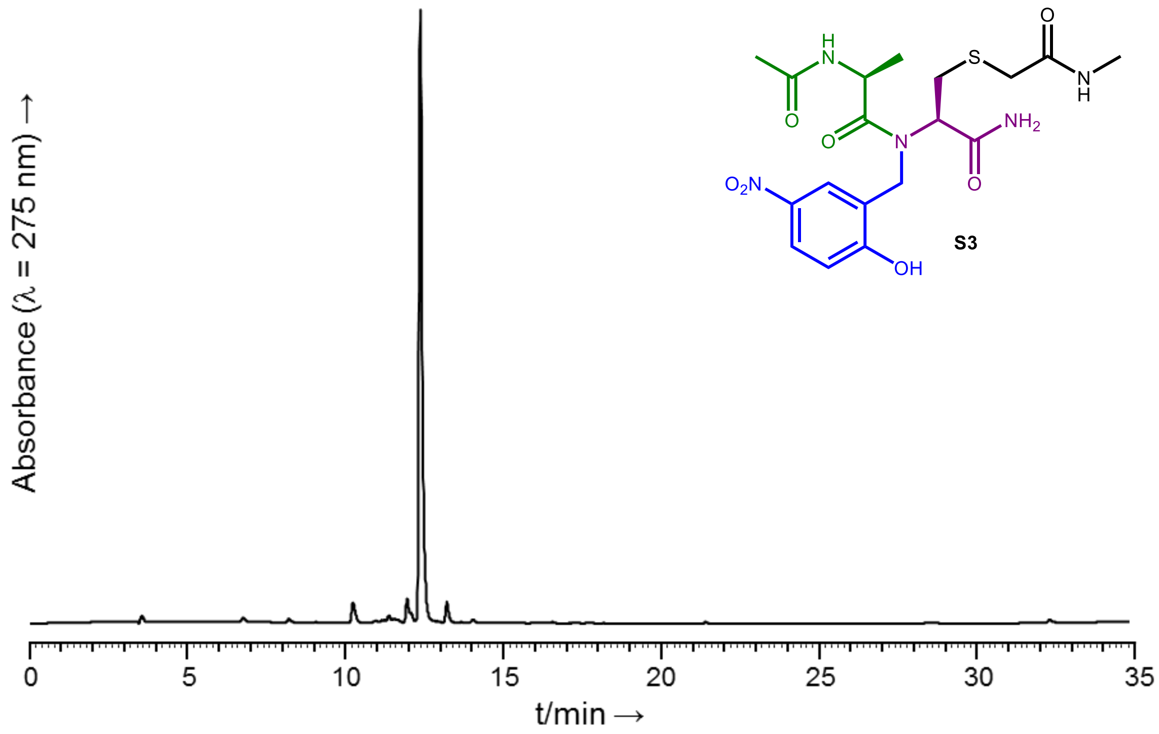

<u>Supplementary figure S10</u>: HPLC trace of crude **S3**.

*<u>Determination of the phenol pKa of S-alkylated compound **S3**</u>*

The pka of the phenol group was determined by acid-base titration of a dilute (10 µM) solution of compound **S3** in Milli-Q water. The pH was adjusted with 1M HCl and NaOH solutions. The titration was followed by UV spectrophotometry on an Uvikon 923 double beam UV/VIS spectrophotometer. The results were plotted at the λ_{max} of

the phenol and phenolate species (determined to be 320 and 407 nm, respectively) against pH.

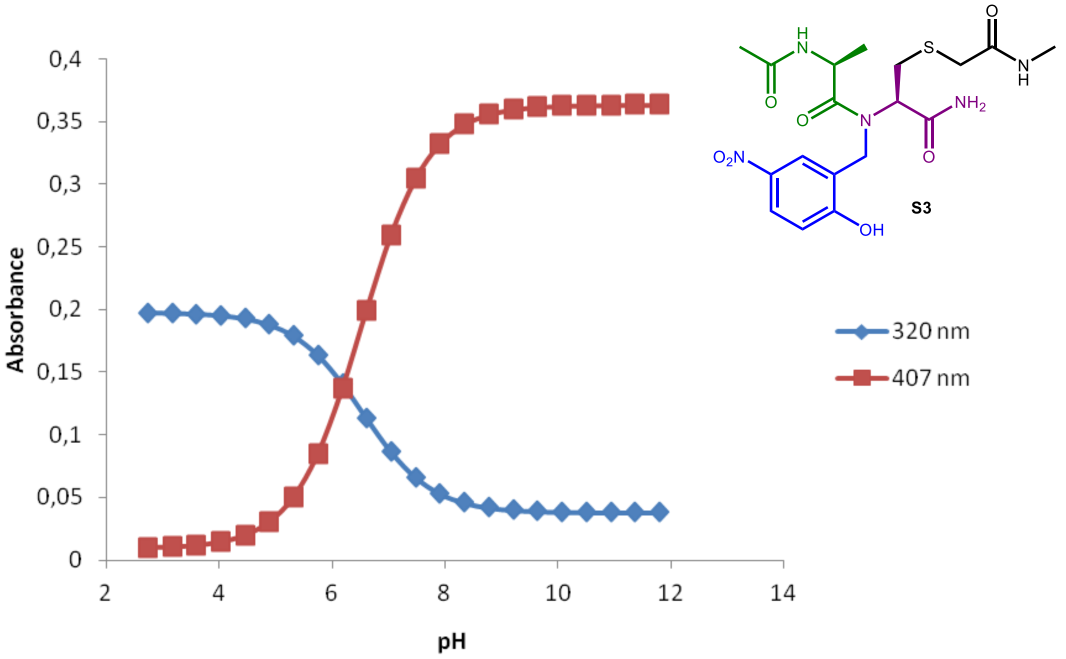

Supplementary figure S11: UV absorbance of compound **S3** as a function of pH.

The pKa value (6.5 ± 0.1) was determined fitting with a sigmoidal function and measuring the inflection point of the curve.

407 nm: y = 0.36324 + (0.00891-0.36324)/(1 + exp((x-6.51822)/ 0.59329)) R^2= 0.996
320 nm: y = 0.0378+ (0.19762- 0.0378)/(1 + exp((x- 6.53917)/ 0.60827)) R^2= 0.993

5- Characterization of a model *N*-acyl-*N*-(Hnb)Cys compound

5a- ^{1}H and ^{13}C NMR of **S5** and comparison with ^{1}H NMR of *O*-methylated **S6**

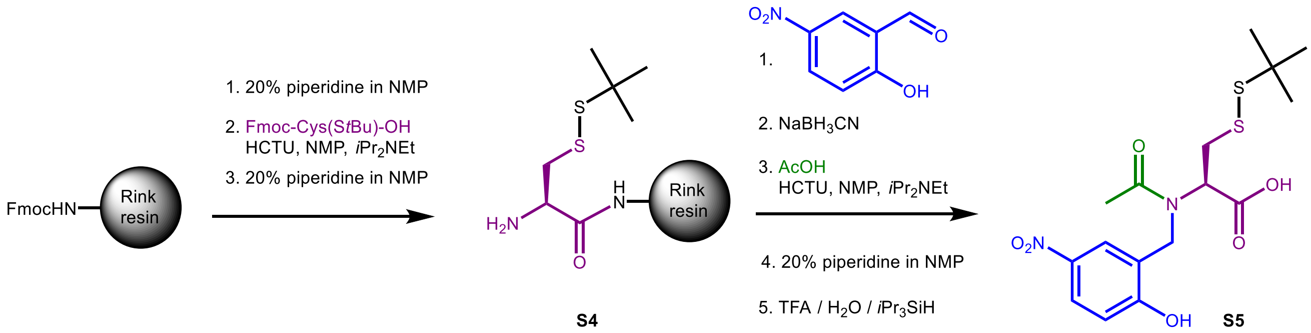

Supplementary scheme S7: Synthesis of the compound **S5**.

Fmoc-protected Rink polystyrene resin (63.3 mg, 0.79 mmol/g, 0.05 mmol) was introduced into a polypropylene syringe fitted with a polypropylene frit and a PTFE stopcock and swollen with NMP. Fmoc group was deprotected by three successive treatments with 20% piperidine in NMP (5 mL) for 3 min. Then, Fmoc-Cys(S*t*Bu)-OH (216 mg, 0.5 mmol, 10 equiv.) and HCTU (186 mg, 0.45 mmol, 9 equiv.) were

dissolved in NMP (1 mL) prior to addition of *i*Pr_2NEt (175 µL, 1 mmol, 20 equiv.). The resulting solution was immediately added to the resin. The syringe was left for 1 h under gentle stirring, then the syringe was drained and the resin was washed with NMP. Fmoc group was removed by three successive treatments with 20% piperidine in NMP (5 mL) for 3 min to give resin **S4**. The 2-hydroxy-5-nitrobenzyl linker was then introduced following the general procedure (p S5 for 0.05 mmol). Then, acetic acid (30 µL, 0.5 mmol, 10 equiv.) and HCTU (186 mg, 0.45 mmol, 9 equiv.) were dissolved in NMP (1 mL) prior to addition of *i*Pr_2NEt (175 µL, 1 mmol, 20 equiv.). The resulting solution was immediately added to the resin. The syringe was left for 1 h under gentle stirring, then the syringe was drained and the resin was washed with NMP. Amino acid-resin was then treated with 20% piperidine in NMP (5 mL, 3 × 3 min), washed with NMP then CH_2Cl_2. Finally, the compound **S5** was obtained after treatment with TFA/H_2O/*i*Pr_3SiH, 93/5/2 (4 mL) for 2 h and characterized. Note that the hydrolysis of the C-terminal amide into the acid carboxylic was quantitative under the cleavage conditions. The product was purified by flash reverse phase chromatography (45 mg crude in 1 mL MeCN, column: RP18 25-40 µm - 20 g, Götec-Labortechnik GmbH, gradient: 30-50% B/A over 20 min, 35 mL/min).

S5:

ESI-HRMS (*m*/*z*): $[MH]^+$ calcd for $C_{16}H_{23}N_2O_6S_2$: 403.0998, found: 403.0993.
HPLC: retention time: 28.36 min (Nucleosil, gradient: 20-50% B/A over 30 min).

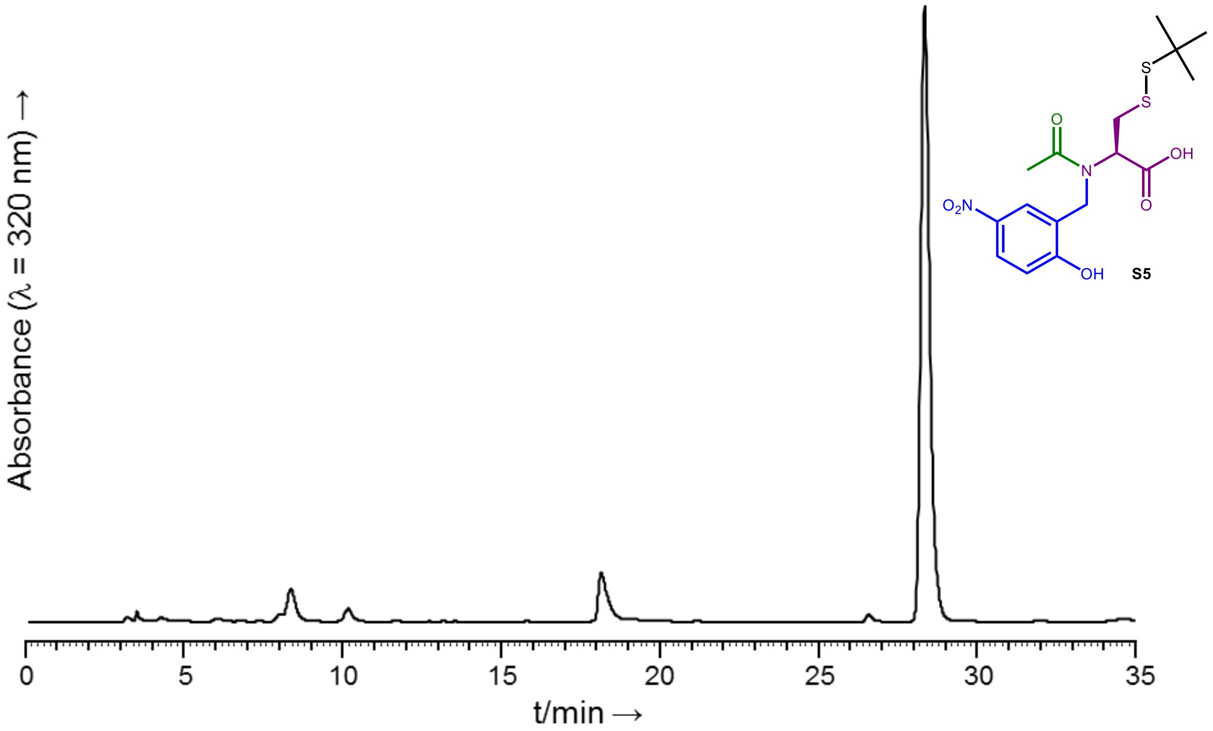

Supplementary figure S12: HPLC trace of crude **S5**.

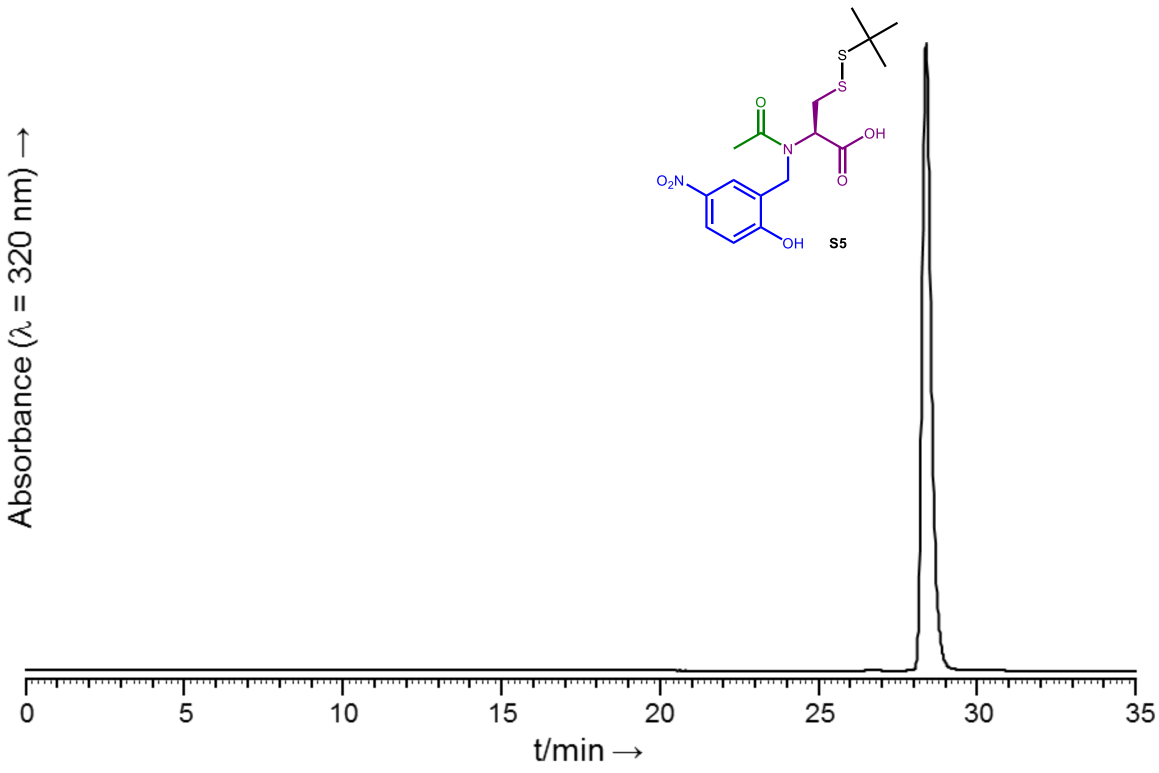

Supplementary figure S13: HPLC trace of pure **S5**.

Pure **S5** has been analyzed by 1H NMR in DMSO-d_6. Two conformers are observed: *trans* and *cis* isomers (evaluated ratio 73:27). On the supplementary figure S14, arrows indicate observed NOE on a ROESY 2D spectrum (mixing time = 200 ms) used to characterize the two conformers.

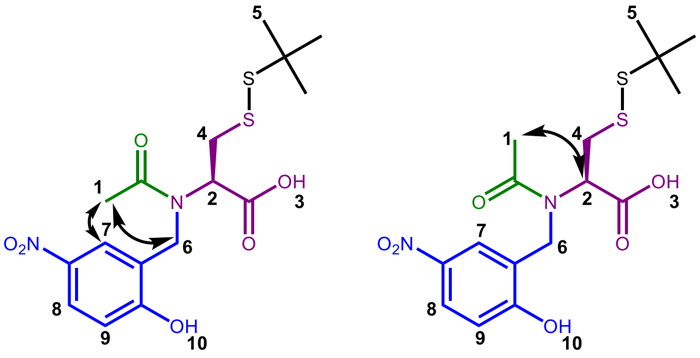

S5
***trans* conformer**

S5
***cis* conformer**

Supplementary figure S14: *Cis* and *trans* isomers of **S5** and observed ROESY connectivities.

***Trans* isomer of S5**

1H NMR (600 MHz, DMSO-*d*$_6$): 13.40 (1H, H_3, s), 11.48 (1H, H_{10}, s), 8.42 (1H, H_7, d, J = 2.8 Hz), 8.10 (1H, H_8, dd, J = 8.9,2.9 Hz), 7.01 (1H, H_9, d, J = 8.9 Hz), 4.64-5.52 (2H, H_{6a} and H_{6b}, m), 4.26 (1H, H_2, t, J = 6.7 Hz), 3.40-3.36 (1H, H_{4b}, m), 2.87 (1H, H_{4a}, dd, J = 13.5,7.5 Hz), 2.06 (3H, H_1, s), 1.21 (9H, H_5, s).

***Cis* isomer of S5**
1H NMR (600 MHz, DMSO-*d_6*): 12.74 (1H, H_3, br), 11.32 (1H, H_{10}, s), 8.04-8.00 (1H, H_8, m), 8.02 (1H, H_7, br), 6.95 (1H, H_9, d, J = 8.7 Hz), 4.85 (1H, H_2, dd, J = 9.6,4.8 Hz), 4.55-4.52 (1H, H_{6b}, m), 4.18 (1H, H_{6a}, d, J = 16.6 Hz), 3.26 (1H, H_{4b}, dd, J = 14,4.8 Hz), 3.01 (1H, H_{4a}, dd, J = 13.9,9.6 Hz), 2.22 (3H, H_1, s), 1.24 (9H, H_5, s).

***Trans* and *cis* isomers of S5 - 27 13C peaks observed** (over 28, the remaining signal probably being hidden by another peak at the same chemical shift and not discernable at the 600 MHz resolution)
13C NMR (100 MHz, DMSO-*d_6*): 171.72, 170.85, 170.80, 161.75, 160.80, 139.63, 139.39, 125.27, 125.16, 125.12, 124.65, 124.54, 124.21, 115.15, 114.98, 99.51, 60.38, 59.42, 48.37, 48.18, 47.60, 45.76, 40.16, 40.06, 29.47, 29.42, 22.04, 21.59.

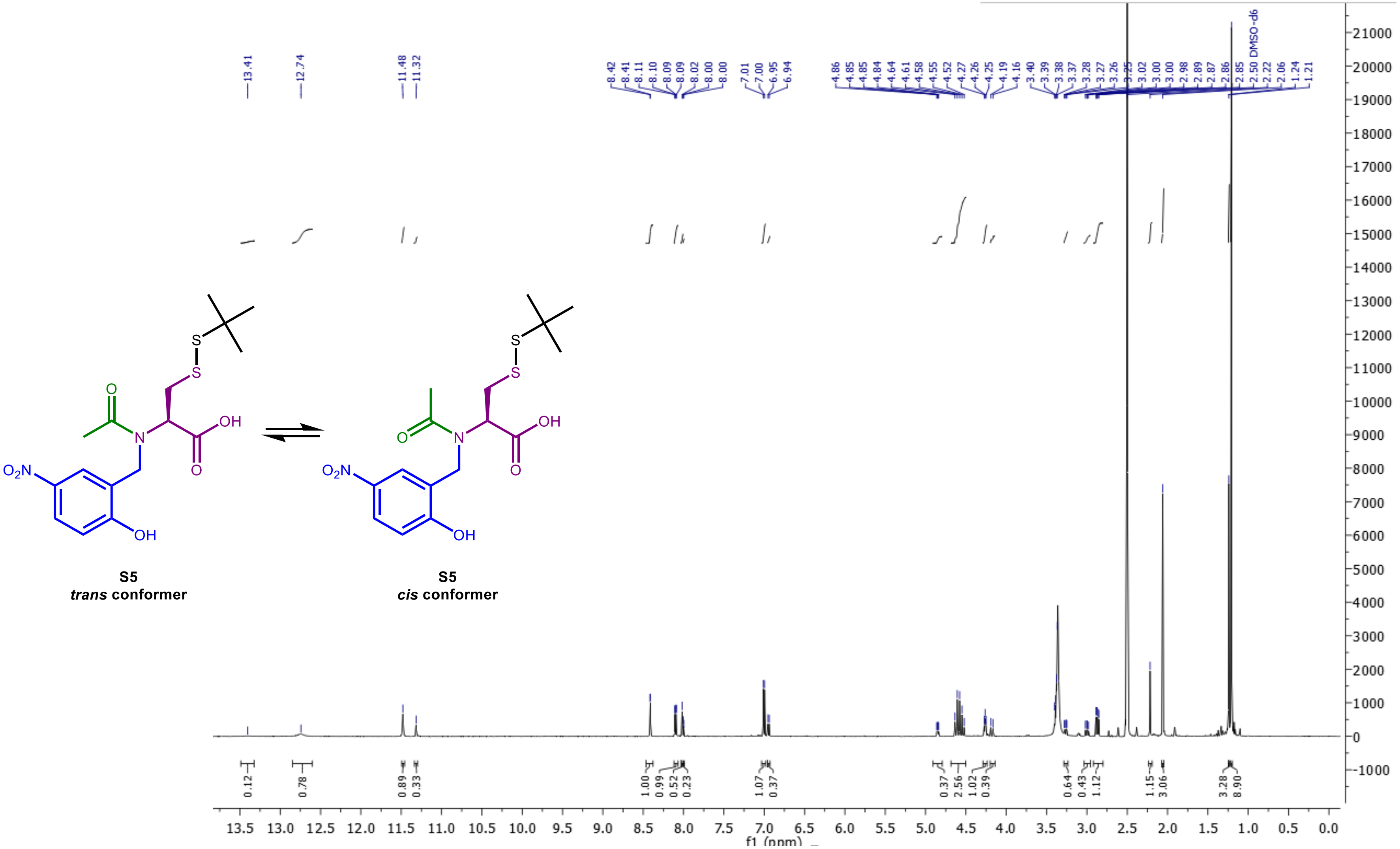

S5
***trans* conformer**

S5
***cis* conformer**

Supplementary figure S15: Copy of the ^{1}H NMR spectrum of **S5**.

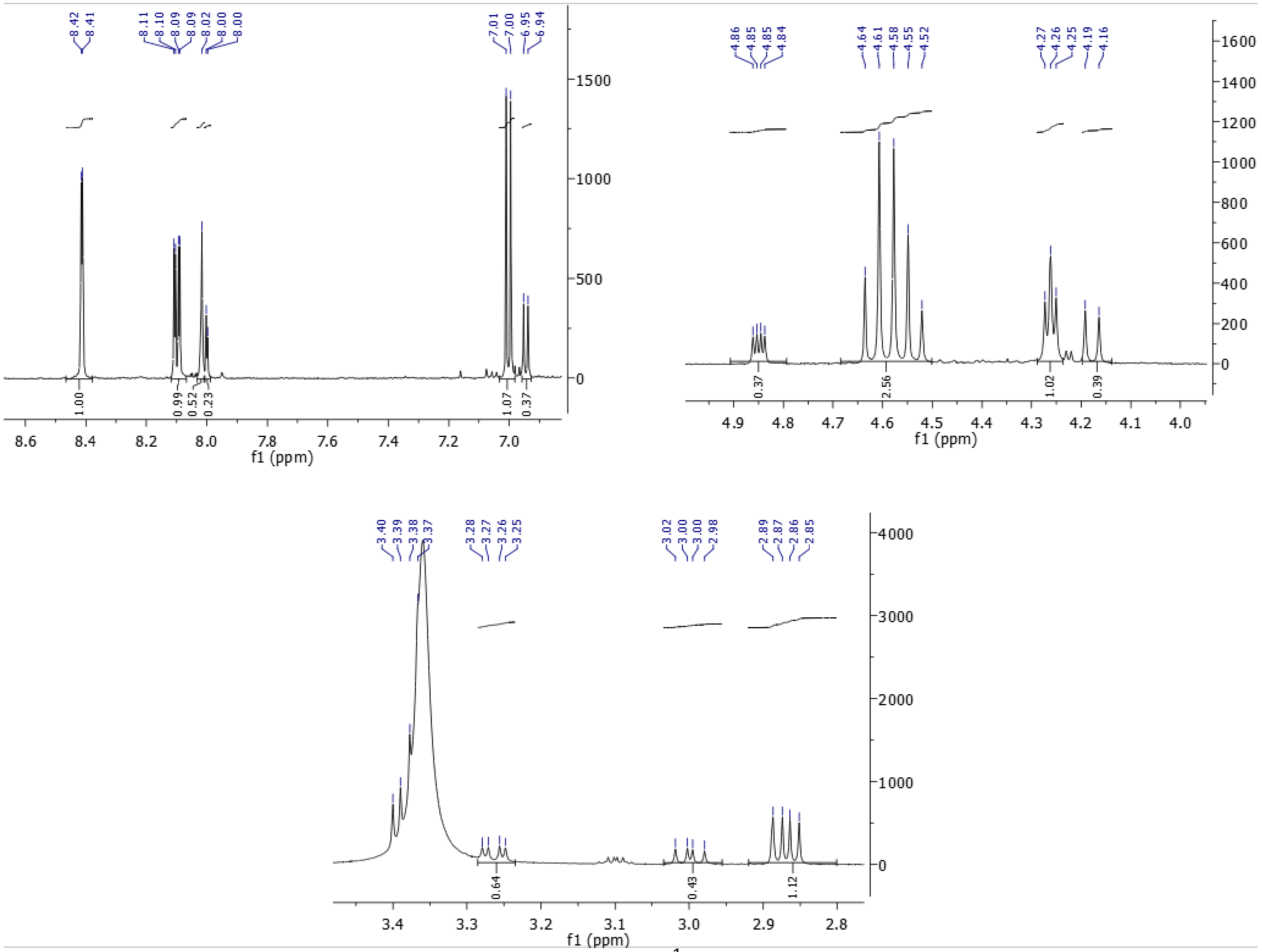

Supplementary figure S16: Copy of the ^{1}H NMR spectrum (zoom) of **S5**.

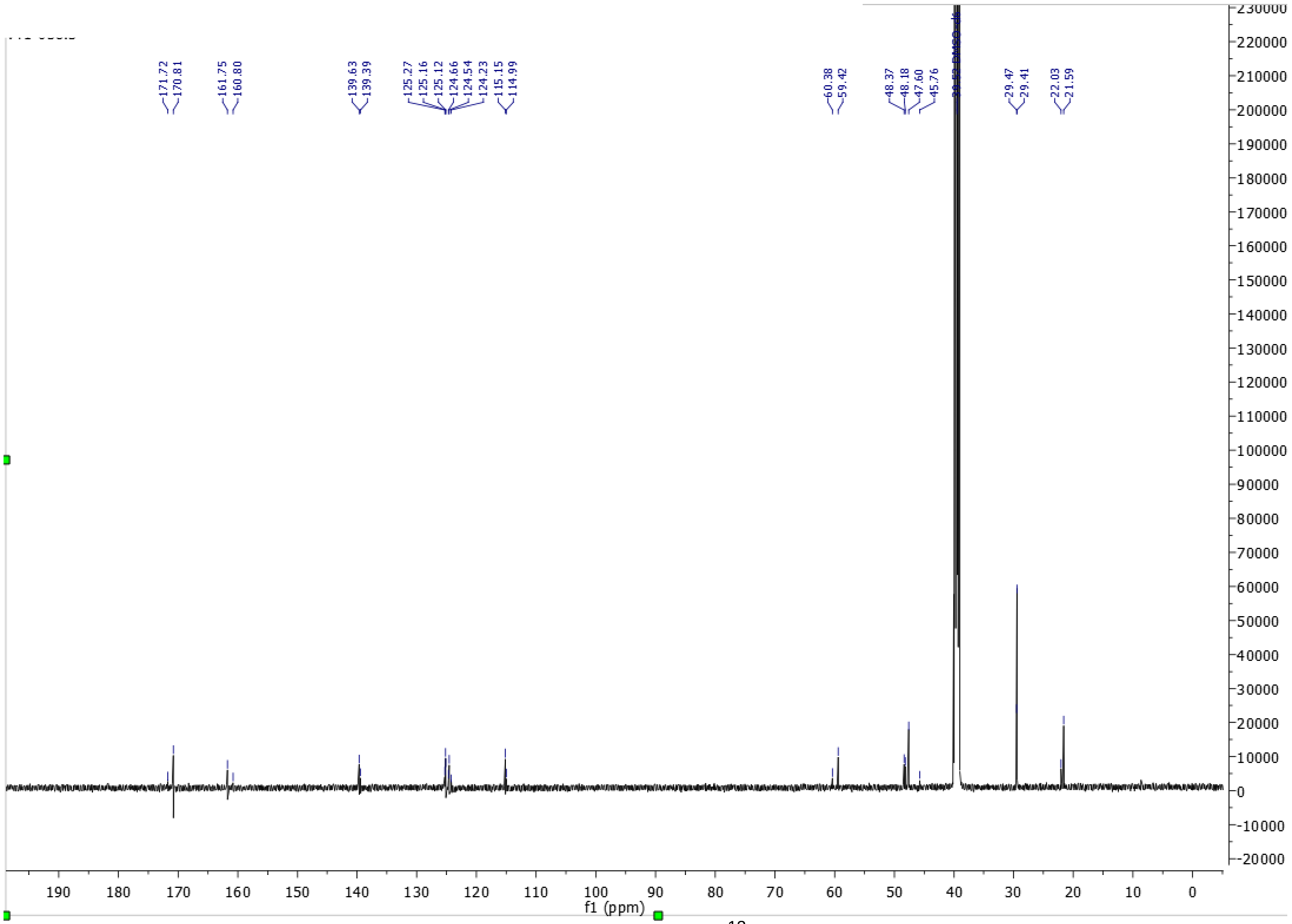

Supplementary figure S17: Copy of the ^{13}C NMR spectrum of **S5**.

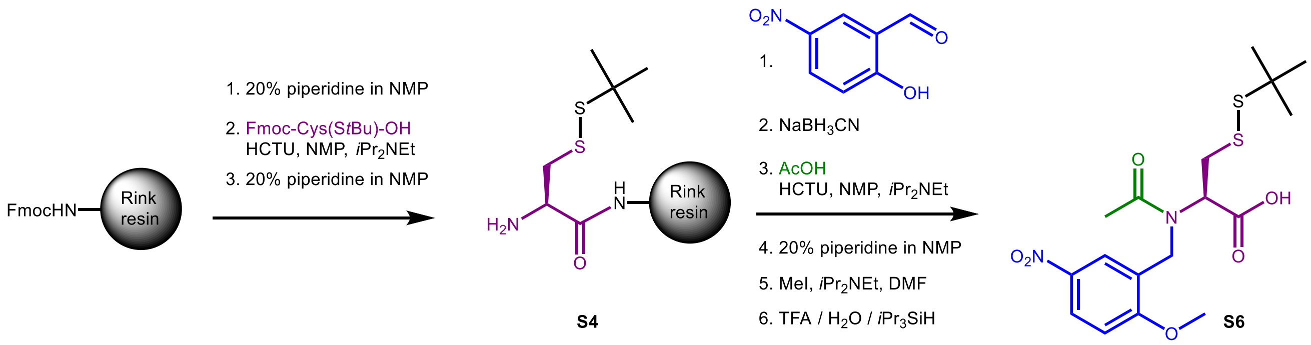

Supplementary scheme S8: Synthesis of the compound **S6**.

Fmoc-protected Rink polystyrene resin (63.3 mg, 0.79 mmol/g, 0.05 mmol) was introduced into a polypropylene syringe fitted with a polypropylene frit and a PTFE stopcock and swollen with NMP. Fmoc group was deprotected by three successive treatments with 20% piperidine in NMP (5 mL) for 3 min. Then, Fmoc-Cys(S*t*Bu)-OH (216 mg, 0.5 mmol, 10 equiv.) and HCTU (186 mg, 0.45 mmol, 9 equiv.) were dissolved in NMP (1 mL) prior to addition of *i*Pr_2NEt (175 µL, 1 mmol, 20 equiv.). The resulting solution was immediately added to the resin. The syringe was left for 1 h under gentle stirring, then the syringe was drained and the resin was washed with NMP. Fmoc group was removed by three successive treatments with 20% piperidine in NMP (5 mL) for 3 min to give resin **S4**. The 2-hydroxy-5-nitrobenzyl linker was then introduced following the general procedure (p S5 for 0.05 mmol). Then, acetic acid (30 µL, 0.5 mmol, 10 equiv.) and HCTU (186 mg, 0.45 mmol, 9 equiv.) were dissolved in NMP (1 mL) prior to addition of *i*Pr_2NEt (175 µL, 1 mmol, 20 equiv.). The resulting solution was immediately added to the resin. The syringe was left for 1 h under gentle stirring, then the syringe was drained and the resin was washed with NMP. Amino acid-resin was then treated with 20% piperidine in NMP (5 mL, 3 × 3 min), washed with NMP then CH_2Cl_2. Methylation of the phenol group was performed using a solution of MeI (50 equiv.), *i*Pr_2NEt (50 equiv.) in DMF for 4 h and the resin was washed with NMP and CH_2Cl_2. Finally, the compound **S6** was obtained after treatment with TFA/H_2O/*i*Pr_3SiH, 93/5/2 (4 mL) for 2 h and characterized. Note that the hydrolysis of the C-terminal amide into the acid carboxylic was quantitative under the cleavage conditions. The product was purified by semi-preparative HPLC (Nucleosil, 40-55 % B/A over 15 min).

S6:

ESI (*m*/*z*): $[MH]^+$ calcd for $C_{17}H_{25}N_2O_6S_2$: 417.1, found: 417.1.
HPLC: retention time: 31.28 min (Nucleosil, gradient: 20-50% B/A over 30 min).

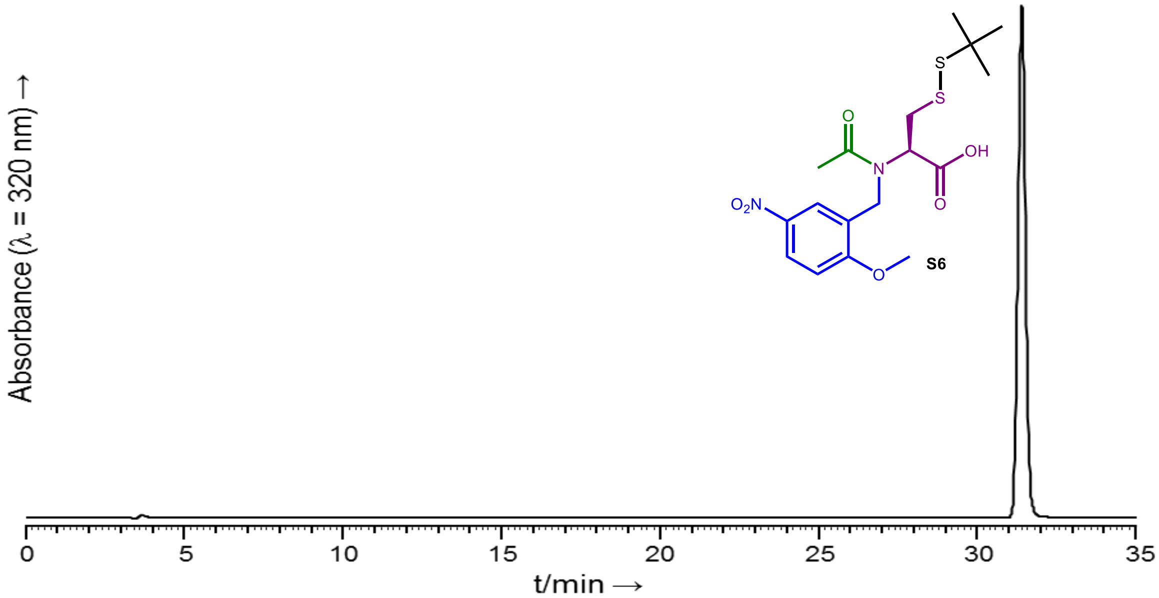

Supplementary figure S18: HPLC trace of pure **S6**.

Pure **S6** has been analyzed by ^{1}H NMR in DMSO-d_6 for comparison of the *cis*-*trans* ratio with **S5**. Two conformers are observed: *trans* and *cis* isomers (evaluated ratio 72:28, nearly identical with **S5** ratio). On the supplementary figure S19, arrows indicate observed NOE on a ROESY 2D spectrum (mixing time = 200 ms) used to characterize the two conformers.

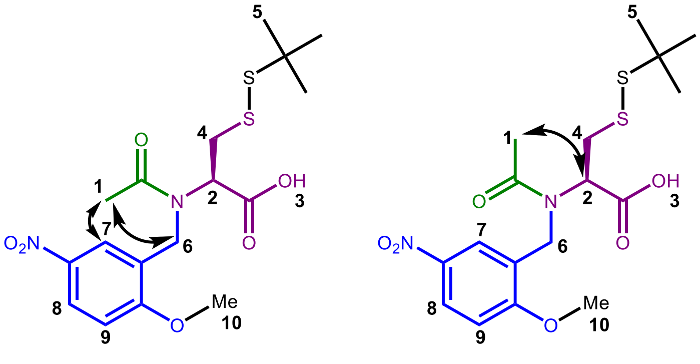

S6
***trans* conformer**

S6
***cis* conformer**

Supplementary figure S19: *Cis* and *trans* isomers of **S6** and observed ROESY connectivities.

***Trans* isomer (major) of S6**

1H NMR (600 MHz, DMSO-*d*$_6$): 12.77 (1H, H_3, bs), 8.46 (1H, H_7, d, *J* = 2.7 Hz), 8.26 (1H, H_8, dd, *J* = 9.1,2.8 Hz), 7.26 (1H, H_9, d, *J* = 9.1 Hz), 4.6 (2H, H_{6a} and H_{6b}, dd, *J* = 45.3,17.3 Hz), 4.25 (1H, H_2, t, *J* = 6.5 Hz), 3.96 (3H, H_{10}, s), 3.36 (1H, H_{4b}, dd, *J* = 13.5,5.8 Hz), 2.91 (1H, H_{4a}, dd, *J* = 13.7,7.8 Hz), 2.04 (3H, H_1, s), 1.22 (9H, H_5, s).

***Cis* isomer (minor) of S6**

1H NMR (600 MHz, DMSO-*d*$_6$): 13.43 (1H, H_3, bs), 8.16 (1H, H_8, dd, *J* = 9.0,2.9 Hz), 8.03 (1H, H_7, d, *J* = 2.7 Hz), 7.19 (1H, H_9, d, *J* = 9.1 Hz), 4.86-4.79 (1H, H_2, m), 4.52 (1H, H_{6b}, t, *J* = 16.9 Hz), 4.19 (1H, H_{6a}, d, *J* = 17.4 Hz), 3.94 (3H, H_{10}, s), 3.29-3.25 (1H, H_{4b}, m), 3.02-2.99 (1H, H_{4a}, m), 2.21 (3H, H_1, s), 1.25 (9H, H_5, s).

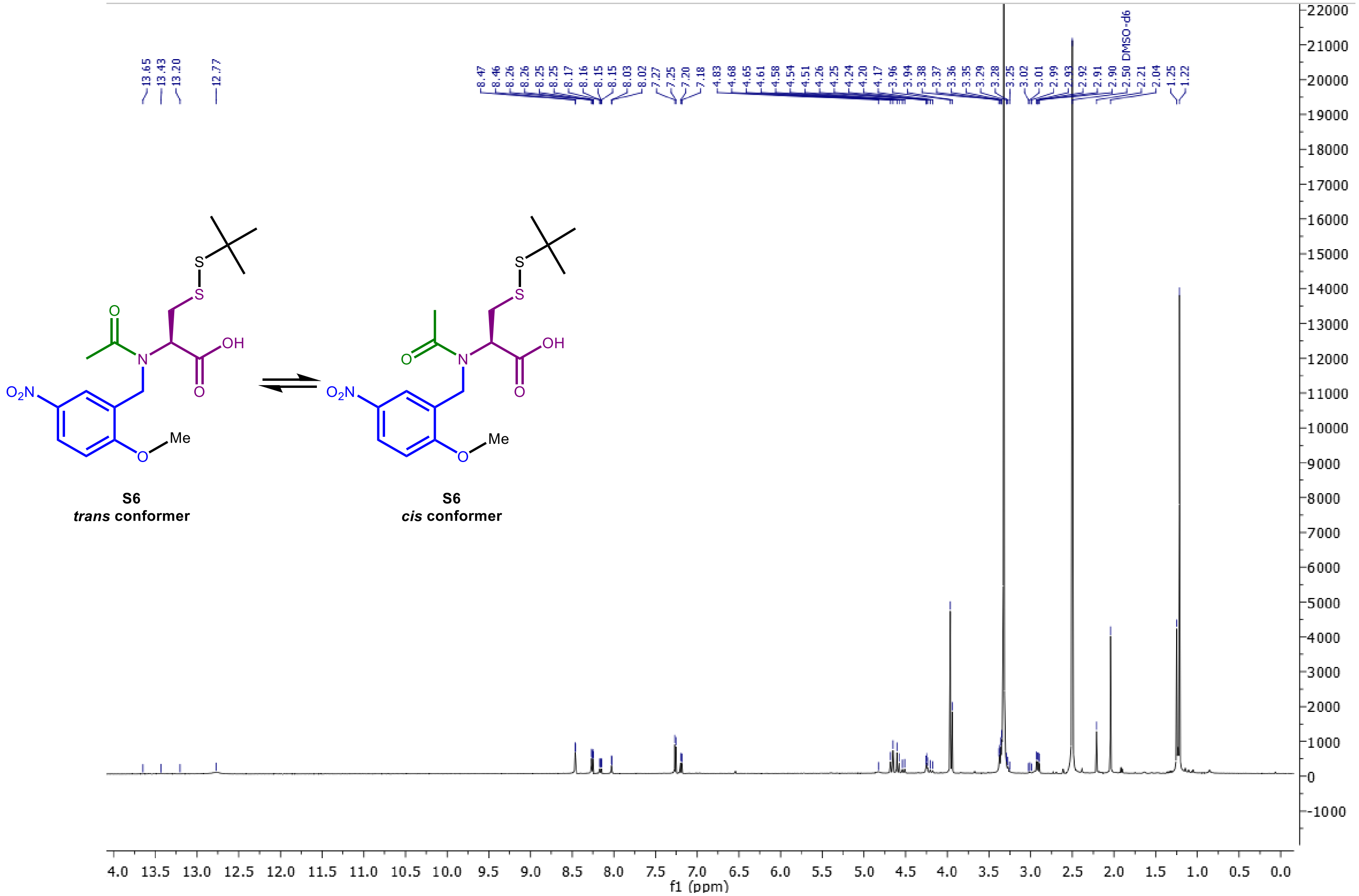

Supplementary figure S20: 1H NMR of **S6**.

5b- Molar extinction coefficient of the Hnb group of **S5**

The molar extinction coefficient (ε) of the Hnb group of **S5** was determined by UV spectroscopy at 275, 280, 320 nm in a H_2O/MeCN/TFA (8:2:0.001) solution.

**Wavelength (nm)**	**275**	**280**	**320**
**ε ($L.mol^{-1}.cm^{-1}$)**	**1846**	**2359**	**6325**

Supplementary table S1: Molar extinction coefficient of **S5**.

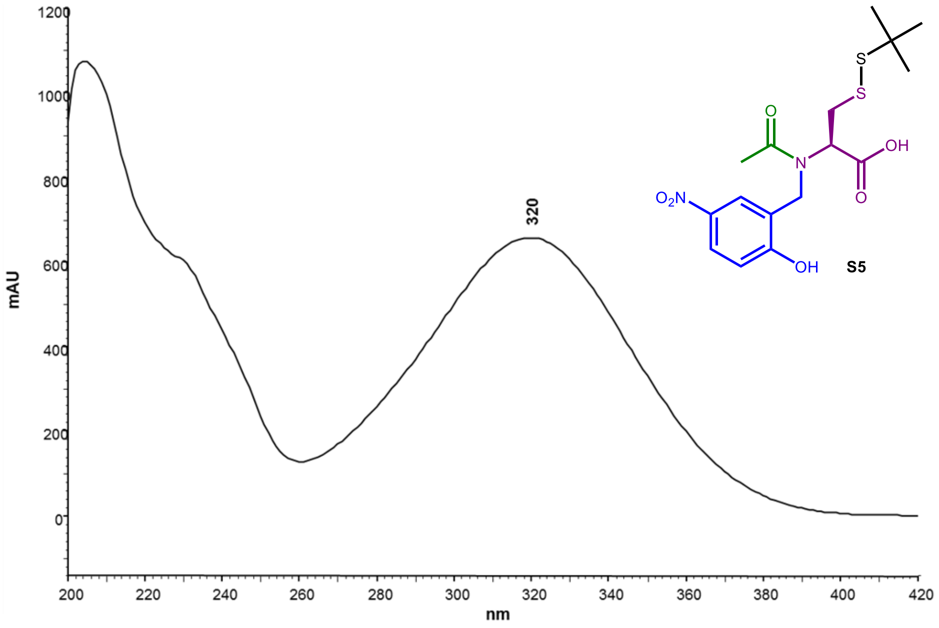

Supplementary figure S21: UV spectrum of **S5**.

6- Optimization of the cysteine thiol protecting group

6a- Synthesis and stability of (*S*-Trt) model peptide **S7**

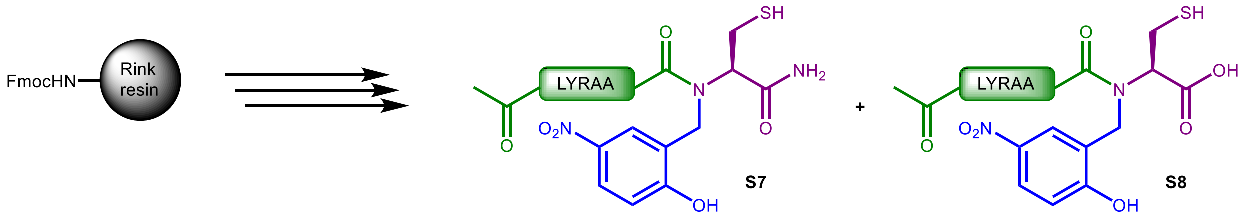

Supplementary scheme S9: Synthesis of the model peptide **S7** showing hydrolysis co-product **S8**.

Fmoc-protected Rink polystyrene resin (126.6 mg, 0.79 mmol/g, 0.1 mmol) was introduced into a polypropylene syringe fitted with a polypropylene frit and a PTFE stopcock and swollen with NMP. Fmoc group was deprotected by three successive treatments with 20% piperidine in NMP (10 mL) for 3 min. Then, Fmoc-Cys(Trt)-OH

(586 mg, 1 mmol, 10 equiv.) and HCTU (372 mg, 0.90 mmol, 9 equiv.) were dissolved in NMP (2 mL) prior to addition of *i*Pr_2NEt (348 µL, 2 mmol, 20 equiv.). The resulting solution was immediately added to the resin. The syringe was left for 1 h under gentle stirring, then the syringe was drained and the resin was washed with NMP. Fmoc group was removed by three successive treatments with 20% piperidine in NMP (10 mL) for 3 min. The 2-hydroxy-5-nitrobenzyl linker was then introduced following the general procedure (p S5). The Ac-LYRAA sequence was installed by standard SPPS procedure (protocol p S3), an extended double coupling (2 × 2h) was performed for the first alanine and a normal double coupling (2 × 30 min) was performed for the arginine. Peptide resin was then treated with 20% piperidine in NMP (10 mL, 3 × 3 min), washed with NMP then CH_2Cl_2, and finally cleaved following the general procedure p S3 to give compound **S7** and side product **S8** (89:11) (Measured by HPLC at 275nm). Peptides were obtained as a mixture of C-terminal amide and acid arising from hydrolysis of the amide under the TFA cleavage conditions.[2] Their corresponding thioester forms arising from a premature *N*-*S* shift was also observed (compounds **S7'** and **S8'**).

S7:

ESI-HRMS (*m/z*): $[MH]^+$ calcd for $C_{39}H_{58}N_{11}O_{11}S$: 888.4038, found: 888.4028.
HPLC: retention time: 4.36 min (Chromolith, gradient: 5-50 % B/A over 5 min).

S8:

ESI-HRMS (*m/z*): $[MH]^+$ calcd for $C_{39}H_{57}N_{10}O_{12}S$: 889.3878, found: 889.3881.
HPLC: retention time: 4.44 min (Chromolith, gradient: 5-50 % B/A over 5 min).

S7':

ESI-HRMS (*m/z*): $[MH]^+$ calcd for $C_{39}H_{58}N_{11}O_{11}S$: 888.4035, found: 888.4028.
HPLC: retention time: 3.82 min (Chromolith, gradient: 5-50 % B/A over 5 min).

S8':

ESI-HRMS (*m/z*): $[MH]^+$ calcd for $C_{39}H_{57}N_{10}O_{12}S$: 889.3884, found: 889.3881.
HPLC: retention time: 3.92 min (Chromolith, gradient: 5-50 % B/A over 5 min).

[2] a) C. J. Creighton, T. T. Romoff, J. H. Bu, M. Goodman, *J. Am. Chem. Soc.* **1999,** *121,* 6786–6791; b) M. Teixidó, F. Albericio, E. Giralt, *J. Peptide Res.* **2005**, *65*, 153–166.

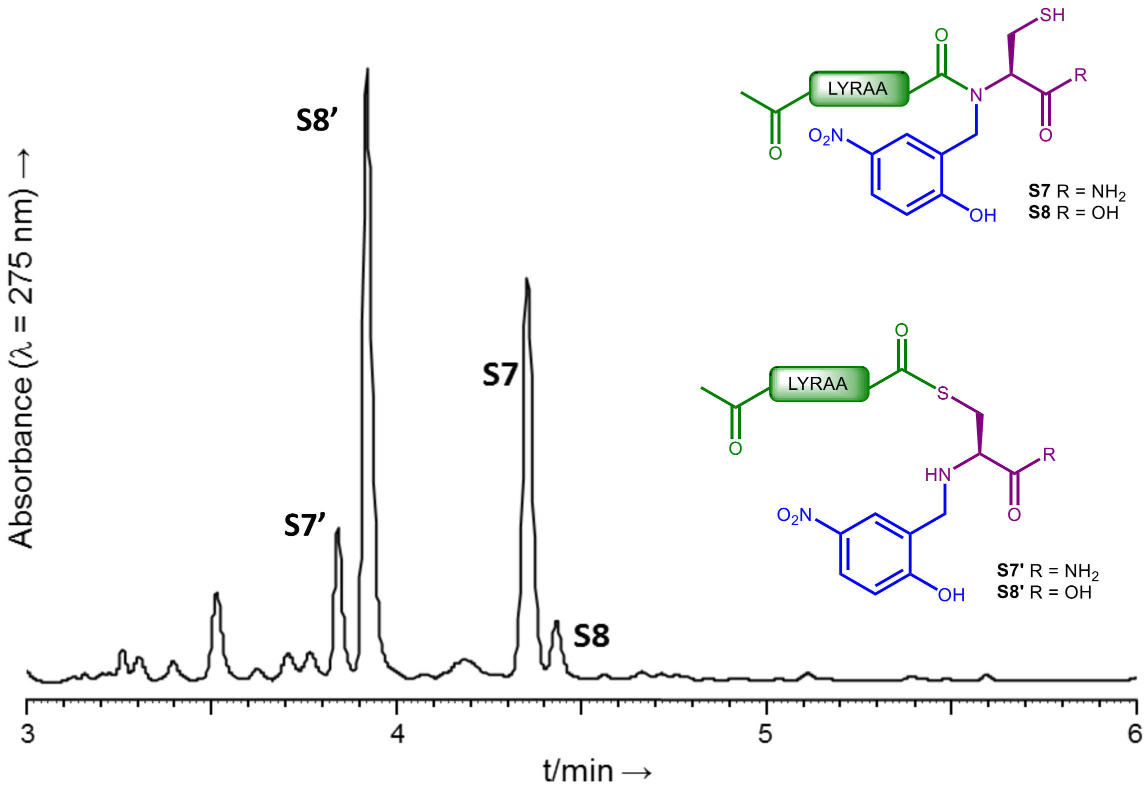

Supplementary figure S22: HPLC trace of crude **S7**.

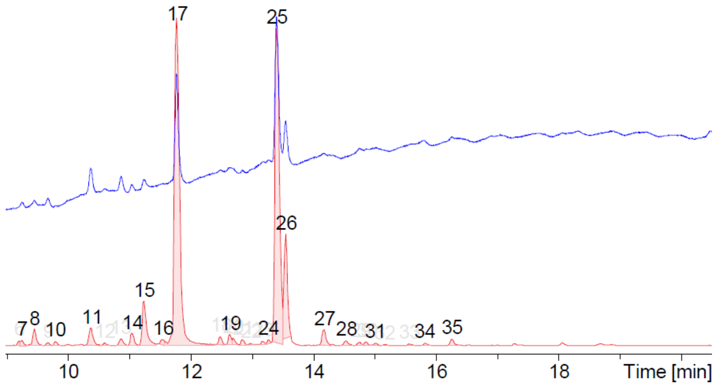

Supplementary figure S23: LC/MS analysis of crude **S7**. Blue trace UV 214 nm, red trace TIC.

Peak (tr (min))	$[MH]^+$ (*m/z*) Calc.	$[MH]^+$ (*m/z*) found	Attributed to
15 (11.24)	888.4038	888.4035	Premature *N*→*S* shift of **S7** (thioester form **S7'**)
17 (11.77)	889.3878	889.3884	Premature *N*→*S* shift of **S8** (thioester form **S8'**)
25 (13.40)	888.4038	888.4028	**S7**
26 (13.55)	889.3878	889.3881	**S8**

Supplementary table S2: Attribution of selected peaks observed in LC/MS analysis of crude **S7**.

To test the stability of this mixture of peptides, 1 mg of crude product was solubilized in 1 mL of a 8:2:0.01 H_2O/MeCN/TFA mixture. The stability of **S7** was studied by analyzing this solution by LC-MS after overnight incubation. Formation of Ac-LYRAA-OH was observed, probably arising from hydrolysis of **S7'** and **S8'**.

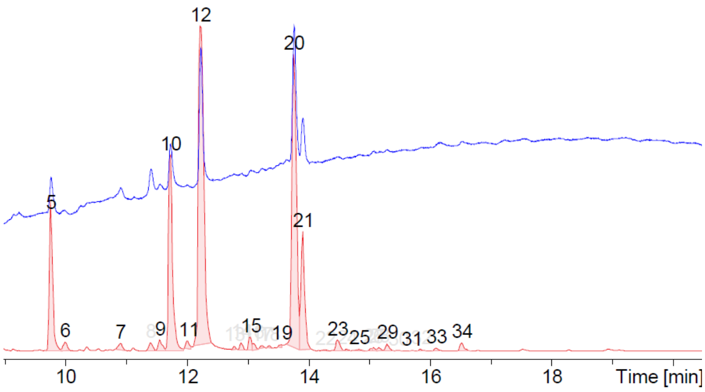

Supplementary figure S24:
LC/MS trace of crude **S7** dissolved in 8:2:0.01 H_2O/MeCN/TFA (1mg/mL), after t = 17.5 h incubation at room temperature. Blue trace UV 214 nm, red trace TIC.

Peak (tr (min))	$[MH]^+$ (*m/z*) Calc.	$[MH]^+$ (*m/z*) found	Attributed to
5 (9.76)	635.3517	635.3516	Ac-LYRAA-OH
10 (11.72)	888.4038	888.4040	Premature *N*→*S* shift of **S7** (thioester form **S7'**)
12 (12.21)	889.3878	889.3888	Premature *N*→*S* shift of **S8** (thioester form **S8'**)
20 (13.75)	888.4038	888.4028	**S7**
22 (13.90)	889.3878	889.3881	**S8**

Supplementary table S3: Attribution of selected peaks observed in LC/MS analysis of crude **S7** after 17.5 h in solvents A/B.

6b- Synthesis and stability of (*S*-*St*Bu) model peptide **7**

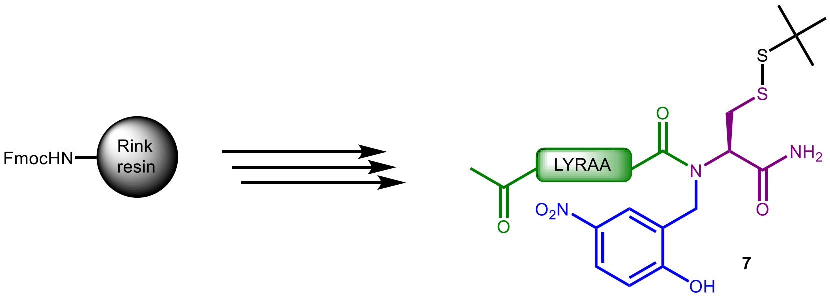

7

Supplementary scheme S10: Synthesis of model peptide **7**.

Fmoc-protected Rink polystyrene resin (126.6 mg, 0.79 mmol/g, 0.1 mmol) was introduced into a polypropylene syringe fitted with a polypropylene frit and a PTFE stopcock and swollen with NMP. Fmoc group was removed by three successive treatments with 20% piperidine in NMP (10 mL) for 3 min. Then, Fmoc-Cys(*St*Bu)-OH (432 mg, 1 mmol, 10 equiv.) and HCTU (372 mg, 0.90 mmol, 9 equiv.) were dissolved in NMP (2 mL) prior to addition of *i*Pr_2NEt (348 µL, 2 mmol, 20 equiv.). The resulting solution was immediately added to the resin. The syringe was left for 1 h under gentle stirring, then the syringe was drained and the resin was washed with NMP. Fmoc group was removed by three successive treatments with 20% piperidine in NMP (10 mL) for 3 min. The 2-hydroxy-5-nitrobenzyl linker was then introduced following the general procedure (p S5). The Ac-LYRAA sequence was installed by standard SPPS procedure (protocol p S3), an extended double coupling (2 × 2h) was performed for the first alanine and a normal double coupling (2 × 30 min) was performed for the arginine. Peptide resin was then treated with 20% piperidine in NMP (10 mL, 3 × 3 min), washed with NMP then CH_2Cl_2, and finally cleaved following the general procedure p S3 to give compound **7**. Peptide was obtained as a mixture of C-terminal amide and acid arising from hydrolysis of the amide under the TFA cleavage conditions.[2]

7:

MALDI-TOF (*m/z*): $[MH]^+$ calcd for $C_{43}H_{66}N_{11}O_{11}S_2$: 976.44, found: 976.45.
HPLC: retention time: 30.89 min (Nucleosil, gradient: 5-50% B/A over 30 min).

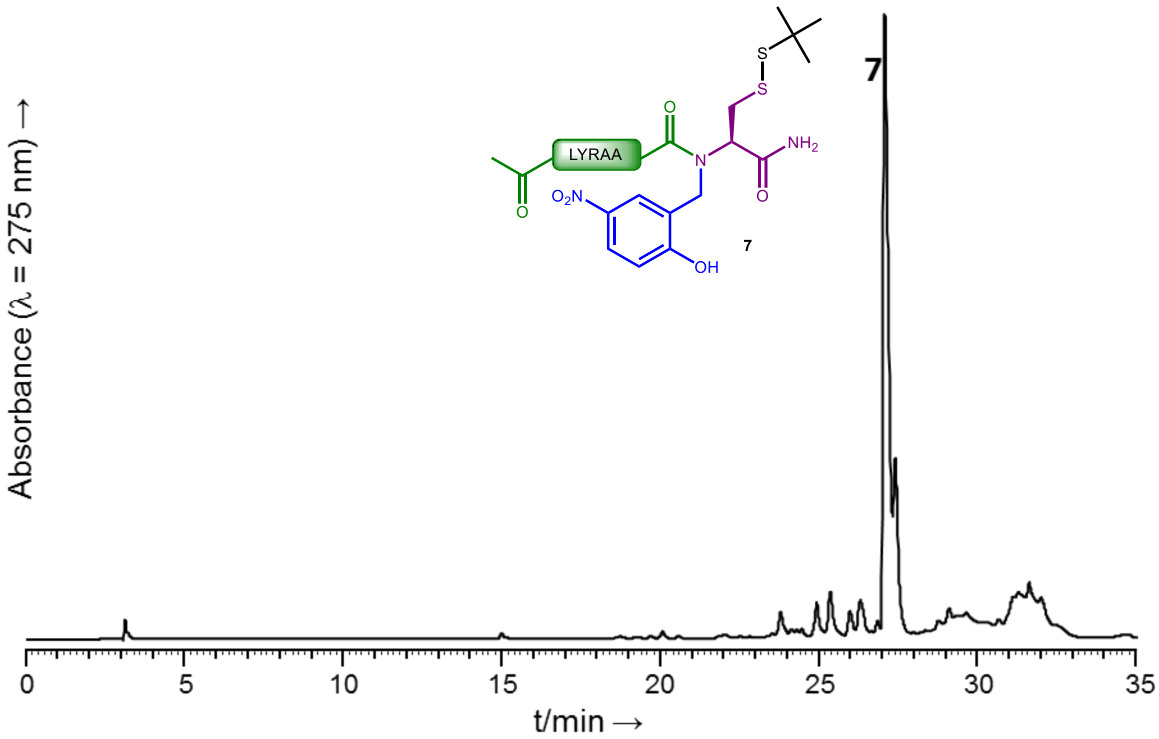

<u>Supplementary figure S25</u>: HPLC trace of crude **7**.

To test the stability of this mixture of peptides, 1 mg of crude product was solubilized in 1 mL of 8:2:0.01 H_2O/MeCN/TFA. The stability of **7** was studied by analyzing this solution by HPLC at different times. Those products are perfectly stable in acidic aqueous conditions.

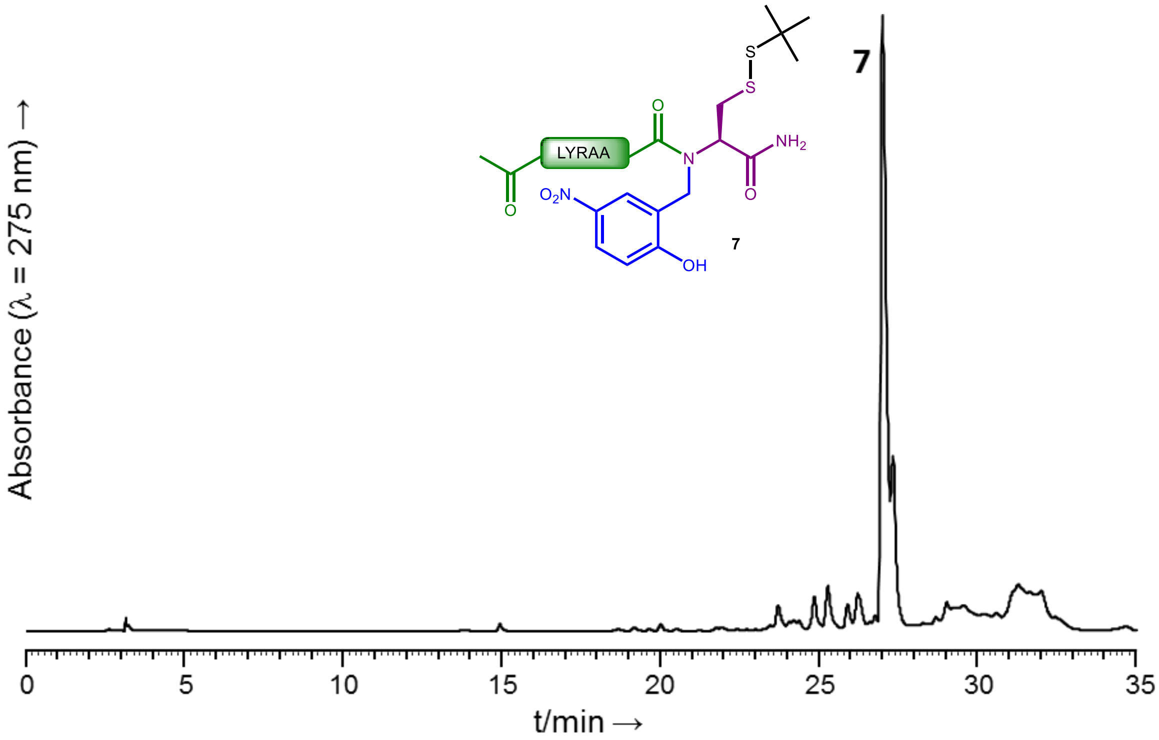

<u>Supplementary figure S26</u>: HPLC trace of crude **7** dissolved in 8:2:0.01 H_2O/MeCN/TFA (1mg/mL), after t = 17.5 h incubation at room temperature.

7- Optimization of the spacer between the cysteine and the resin

7a- Direct attachment to Rink linker – amide hydrolysis co-product[2]

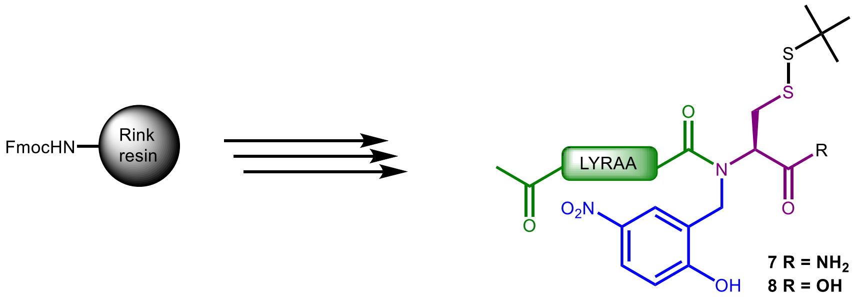

Supplementary scheme S11: Synthesis of the model peptide **7**.

During the cleavage step (using procedure p S3) in order to obtain peptide **7**, the formation of peptide **8** was also observed (12 % determined by HPLC integration at 275 nm) from the hydrolysis of the C-terminal amide function into a C-terminal carboxylic acid.

7:

MALDI-TOF (*m/z*): $[MH]^+$ calcd for $C_{43}H_{66}N_{11}O_{11}S_2$: 976.44, found: 976.45.

HPLC: retention time: 30.89 min (Nucleosil, gradient: 5-50% B/A over 30 min).

8 – C-terminal hydrolysis co-product:

MALDI-TOF (*m/z*): $[MH]^+$ calcd for $C_{43}H_{65}N_{10}O_{12}S_2$: 977.42, found: 977.43.

HPLC: retention time: 31.27 min (Nucleosil, gradient: 5-50% B/A over 30 min).

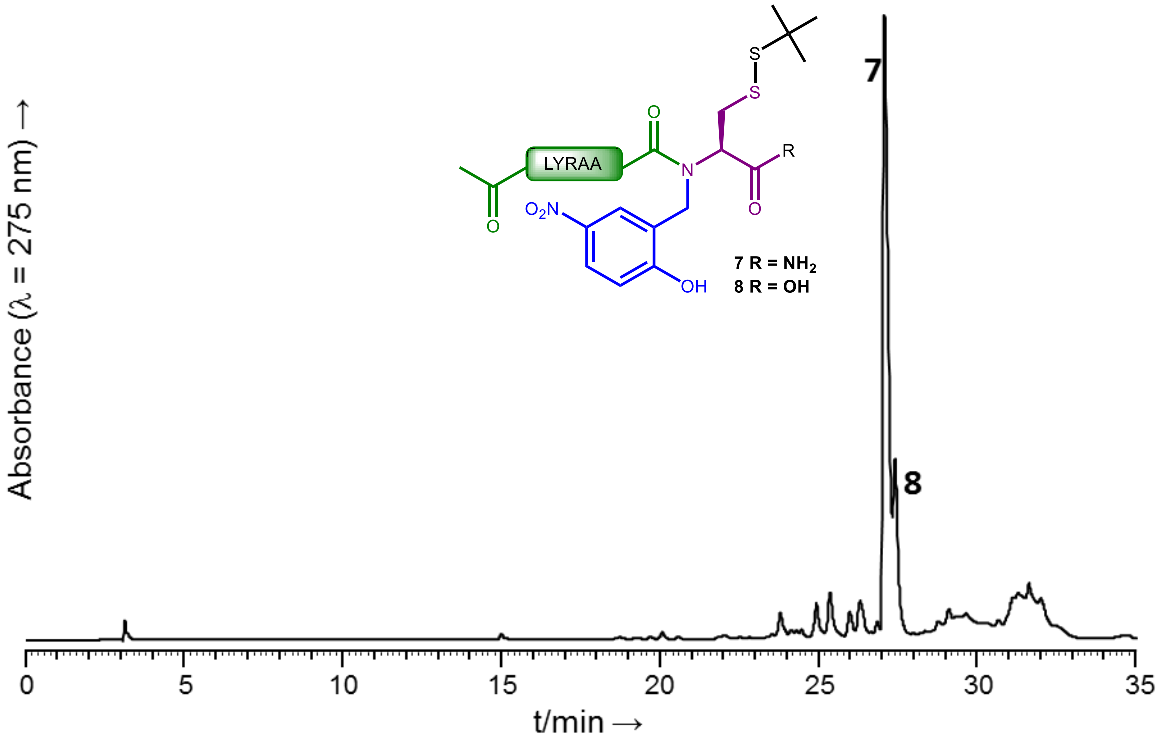

Supplementary figure S27: HPLC trace of crude **7** showing hydrolysis side product **8**.

7b- Direct attachment to PHB Tentagel resin (Wang type linker)

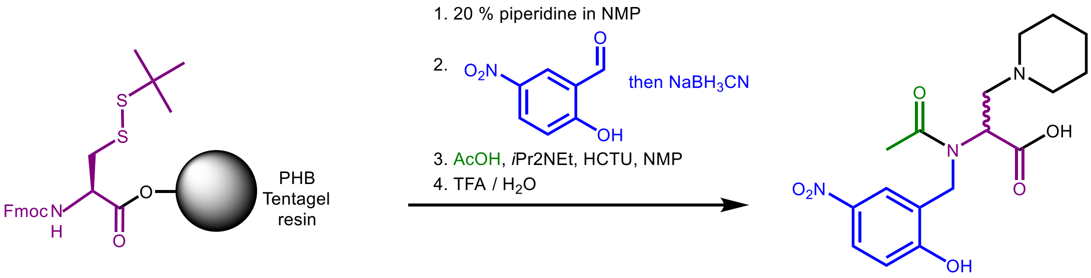

S9

Supplementary scheme S12: Formation of the C-terminal piperidine adduct **S9**.[3]

Pre-loaded Fmoc-Cys(S*t*Bu)-PHB Tentagel resin (527 mg, 0.19 mmol/g, 0.1 mmol) was introduced into a polypropylene syringe fitted with a polypropylene frit and a PTFE stopcock and swollen with NMP. Fmoc group was removed by three successive treatments with 20% piperidine in NMP (10 mL) for 3 min. The 2-hydroxy-5-nitrobenzyl linker was then introduced following the general procedure (p S5). For a 10 µmol aliquot, acetic acid (6 µL, 0.1 mmol, 10 equiv.) and HCTU (37.2 mg, 0.09 mmol, 9 equiv.) were dissolved in NMP (0.2 mL) prior to addition of *i*Pr_2NEt (35 µL, 0.2 mmol, 20 equiv.). The resulting solution was immediately added to the resin. The syringe was left for 1 h under gentle stirring, then the syringe was drained and the resin was washed with NMP. Amino acid-resin was then treated with 20% piperidine in NMP (1 mL, 3 × 3 min), washed with NMP then CH_2Cl_2. Finally, the product was cleaved using TFA / H_2O (95:5) for 2h. The solvents were removed by evaporation and analysis showed the complete formation of the C-terminal piperidine adduct **S9** and no trace of the expected Cys(S*t*Bu) compound.

S9:

ESI-HRMS (*m/z*): $[MH]^+$ calcd for $C_{17}H_{24}N_3O_6$: 366.1665, found: 366.1662.
HPLC: retention time: 6.50 min (Nucleosil, gradient: 20-50% B/A over 30 min).

[3] Lukszo J., Patterson D., Albericio F. , Kates S. A., *Letters in Peptide Science,* 1996, **3**, 157-166

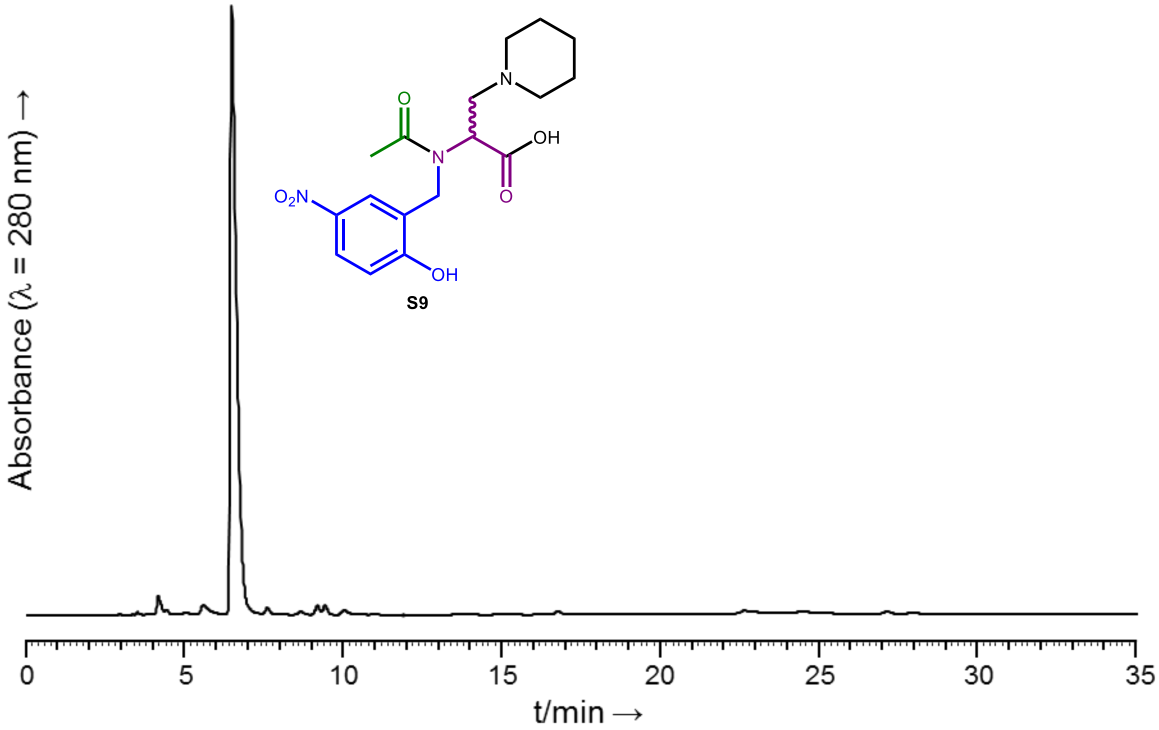

Supplementary figure S28: HPLC trace of crude **S9**.

7c- Introduction of a Gly residue as a spacer

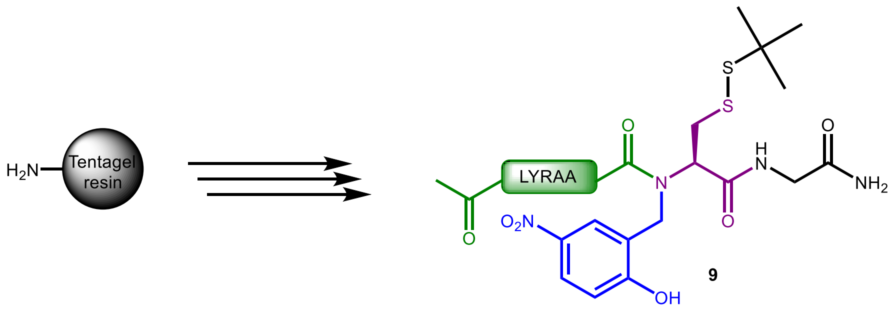

Supplementary scheme S13: Synthesis of the model peptide **9**.

Tentagel R resin (476 mg, 0.21 mmol/g, 0.1 mmol) was introduced into a polypropylene syringe fitted with a polypropylene frit and a PTFE stopcock and swollen with NMP. Fmoc group was removed by three successive treatments with 20% piperidine in NMP (10 mL) for 3 min. Then, Fmoc-Rink-OH (270 mg, 0.5 mmol, 5 equiv.) and HATU (190 mg, 0.5 mmol, 5 equiv.) were dissolved in NMP (2 mL) prior to addition of *i*Pr_2NEt (175 µL, 1 mmol, 10 equiv.). The resulting solution was immediately added to the resin. The syringe was left for 1.5 h under gentle stirring, then the syringe was drained and the resin was washed with NMP. Fmoc group was removed by three successive treatments with 20% piperidine in NMP (10 mL) for 3 min. Fmoc-Gly-OH (297 mg, 1 mmol, 10 equiv.) and HCTU (372 mg, 0.90 mmol, 9 equiv.) were dissolved in NMP (2 mL) prior to addition of *i*Pr_2NEt (348 µL, 2 mmol, 20 equiv.). The resulting solution was immediately added to the resin. The syringe was left for 1 h under gentle stirring, then the syringe was drained and the resin was

washed with NMP. Then, Fmoc group was removed by three successive treatments with 20% piperidine in NMP (10 mL) for 3 min. Fmoc-Cys(S*t*Bu)-OH (432 mg, 1 mmol, 10 equiv.) and HCTU (372 mg, 0.90 mmol, 9 equiv.) were dissolved in NMP (10 mL) prior to addition of *i*Pr_2NEt (348 µL, 2 mmol, 20 equiv.). The resulting solution was immediately added to the resin. The syringe was left for 1.5 h under gentle stirring, then the syringe was drained and the resin was washed with NMP. Fmoc group was removed by three successive treatments with 20% piperidine in NMP (10 mL) for 3 min. The 2-hydroxy-5-nitrobenzyl linker was then introduced following the general procedure (p S5). The Ac-LYRAA sequence was installed by standard SPPS procedure (protocol p S3), an extended double coupling (2 × 2h) was performed for the first alanine and a normal double coupling (2 × 30 min) was performed for the arginine. Peptide resin was then treated with 20% piperidine in NMP (10 mL, 3 × 3 min), washed with NMP then CH_2Cl_2, and finally cleaved following the general procedure p S3 to give compound **9**. The product was purified by HPLC (Nucleosil, 40-45 % B/A over 10 min).

9:

MALDI-TOF (*m/z*): $[MH]^+$ calcd for $C_{45}H_{69}N_{12}O_{12}S_2$: 1033.45, found: 1033.49.
HPLC: retention time: 8.70 min (Chromolith, gradient: 5-50% B/A over 10 min).

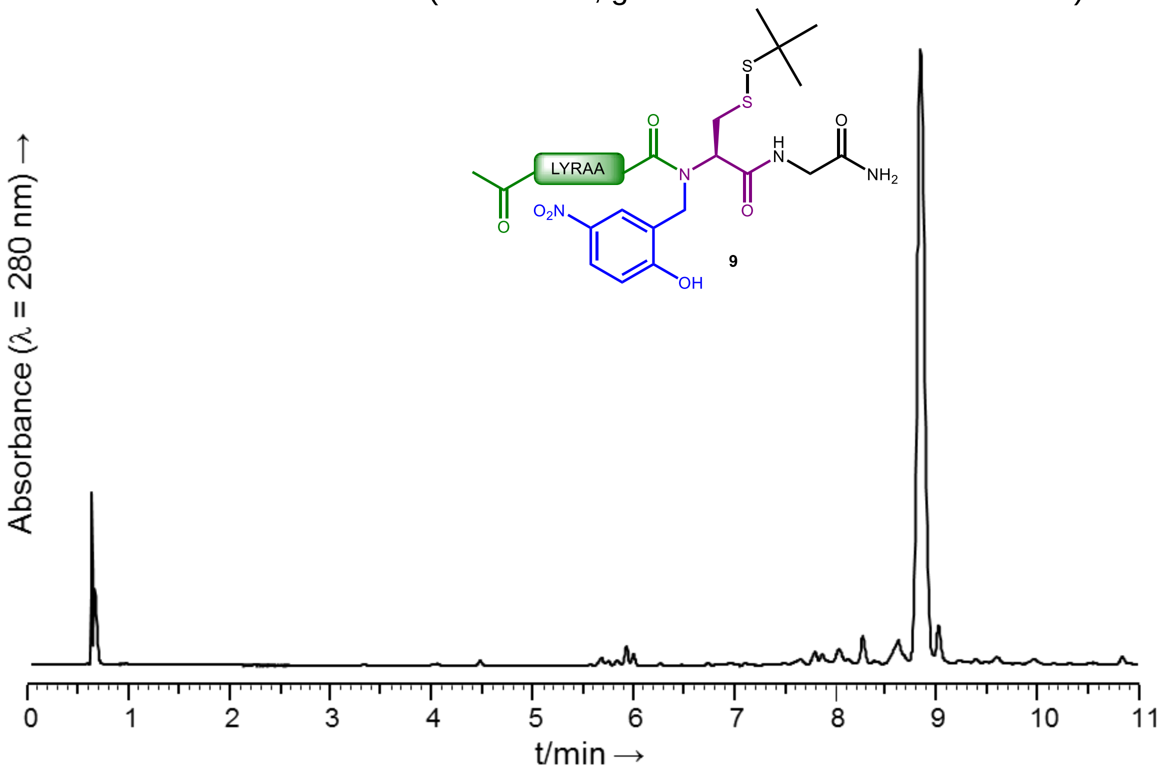

Supplementary figure S29: HPLC trace of crude **9**.

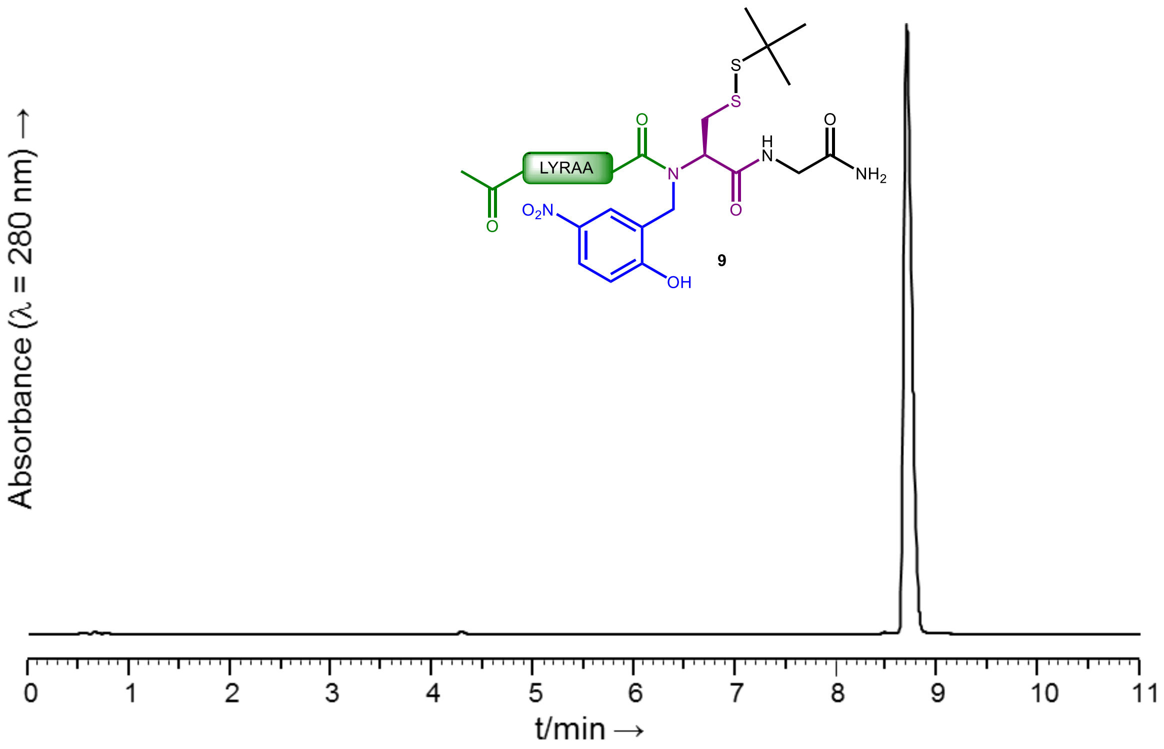

Supplementary figure S30: HPLC trace of pure **9**.

8- *N*-Acylation with the 20 different proteogenic amino acids

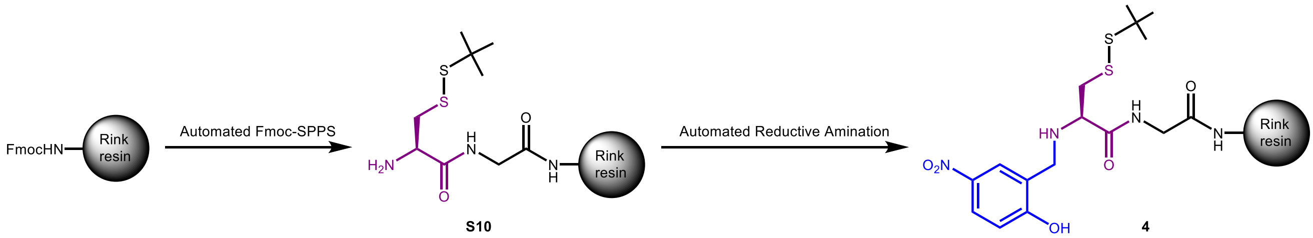

Supplementary scheme S14: Synthesis of (Hnb)-dipeptide **4**.

The Rink linker, Gly and Cys(S*t*Bu) were installed through automated SPPS (protocol p S3) starting from Tentagel R resin (120 mg, 0.21 mmol/g, 25 µmol) in order to obtain peptide resin **S10**.

Automated reductive amination procedure for a peptide synthesizer

Peptide resin **S10** (25 µmol) was introduced into a Prelude synthesizer reactor, washed two times with 3 mL of a DMF/MeOH (1:1) mixture for 30 sec, then swollen in 3 mL of a DMF/MeOH/AcOH (9:9:2) mixture for 5 min. The reactor was drained off and the resin was washed four times with 3 mL of a DMF/MeOH (1:1) mixture for 30 s. 2 mL of a solution of 2-hydroxy-5-nitrobenzaldehyde in DMF/MeOH (1:1) (125 mM, 10 equiv.) were added then the reactor was left for 1h under stirring by nitrogen bubbling. The reactor was drained off and the resin was washed four times with 3 mL of DMF/MeOH (1:1) for 15 sec. Without delay, 2 mL of a fresh solution of sodium

cyanoborohydride in a DMF/MeOH/AcOH (9:9:2) mixture (250 mM, 20 equiv.) were added and the reactor was left for 1 h under stirring by nitrogen bubbling. The reactor was drained and the resin was washed with DMF/MeOH (1:1) (3 mL, 30 sec, × 4), NMP (3 mL, 30 s, × 3), 20% piperidine in NMP (3 mL, 30 s, × 3), NMP (3 mL, 30 sec, × 3), dichloromethane (5 mL, 30 s, × 3) and NMP (3 mL, 30 s, × 2).

**Resin conditioning**			
Operation	Reagent/Solvent	Volume	Time
1) DMF/MeOH wash	DMF/MeOH (1:1)	3 mL × 2	0.5 min × 2
2) DMF/MeOH/AcOH wash	DMF/MeOH/AcOH (9:9:2)	3 mL	5 min
3) DMF/MeOH wash	DMF/MeOH (1:1)	3 mL × 4	0.5 min × 4
**Imine formation**			
Operation	Reagent/Solvent	Volume	Time
1) Add 2-hydroxy- 5-nitrobenzaledhyde (Hnba)	125 mM Hnba in DMF/MeOH (1:1)	2 mL	60 min
2) DMF/MeOH wash	DMF/MeOH (1:1)	3 mL × 4	0.25 min ×4
**Reduction**			
Operation	Reagent/Solvent	Volume	Time
1) Add $NaBH_3CN$	250 mM $NaBH_3CN$ in DMF/MeOH/AcOH (9:9:2)	2 mL	60 min
2) DMF/MeOH wash	DMF/MeOH (1:1)	3 mL × 4	0.5 min × 4
3) NMP wash	NMP	3 mL × 3	0.5 min × 3
4) Base wash	20 % piperidine in NMP	3 mL × 3	3 min × 3
5) NMP wash	NMP	3 mL × 3	0.5 min × 3
6) Dichloromethane wash	CH_2Cl_2	5 mL × 3	0.5 min × 3
7) NMP Wash	NMP	3 mL × 2	0.5 min × 2

N-acylation yield determination

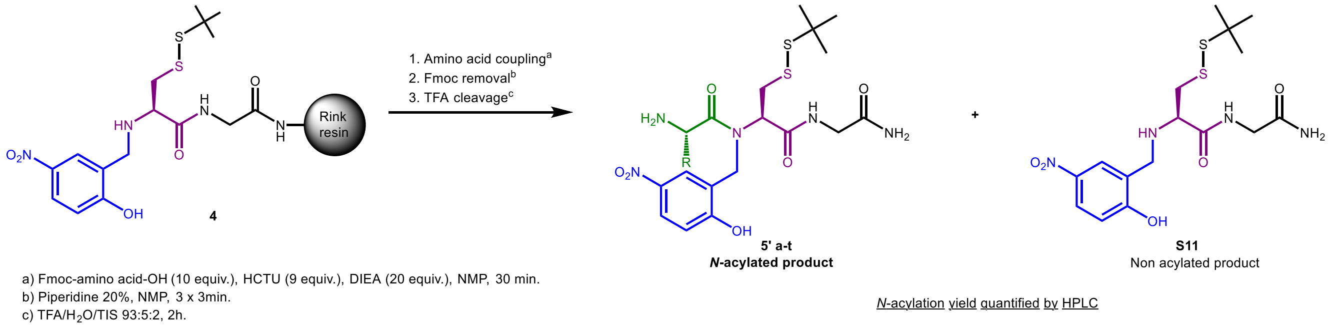

a) Fmoc-amino acid-OH (10 equiv.), HCTU (9 equiv.), DIEA (20 equiv.), NMP, 30 min.
b) Piperidine 20%, NMP, 3 x 3min.
c) $TFA/H_2O/TIS$ 93:5:2, 2h.

Supplementary scheme S15: *N*-Acylation of Hnb-peptide-resin **4**.

The 20 different proteogenic amino acids were then coupled on **4** using a single standard automated coupling on the prelude (protocol p S3), after that the peptide resins were treated with 20 % piperidine in NMP (3 × 3 min); finally, an aliquot (5 µmol) of each resins was cleaved using 1mL of a $TFA/H_2O/iPr_3SiH$ (93:5:2) solution for 2 h. After evaporation of TFA, the samples were analyzed by HPLC; the *N*-acylation yields were quantified by integration at 315 nm, not taking into account eventual differences in molar extinction coefficient of the products.

Non acylated product - S11:

ESI-HRMS (*m/z*): $[MH]^+$ calcd for $C_{16}H_{25}N_4O_5S_2$: 417.1266, found: 417.1261.

HPLC: retention time: 2.12 min (Chromolith, gradient: 20-50 % B/A over 5 min).

Ala - 5'c:

ESI-HRMS (*m/z*): $[MH]^+$ calcd for $C_{19}H_{30}N_5O_6S_2$: 488.1637, found: 488.1636.

HPLC: retention time: 3.55 min (Chromolith, gradient: 20-50 % B/A over 5 min).

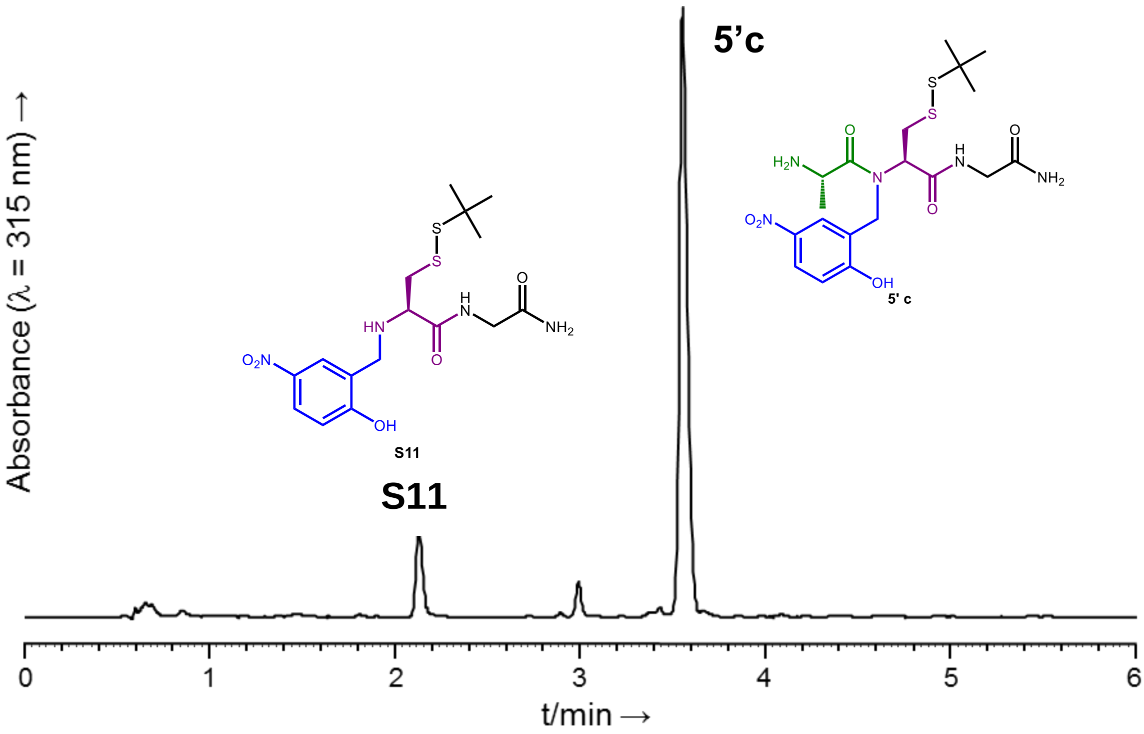

Supplementary figure S31: HPLC trace of crude **5'c**.

Arg - 5’m:

ESI-HRMS (*m/z*): $[MH]^+$ calcd for $C_{22}H_{37}N_8O_6S_2$: 573.2277, found: 573.2272.

HPLC: retention time: 2.54 min (Chromolith, gradient: 20-50 % B/A over 5 min).

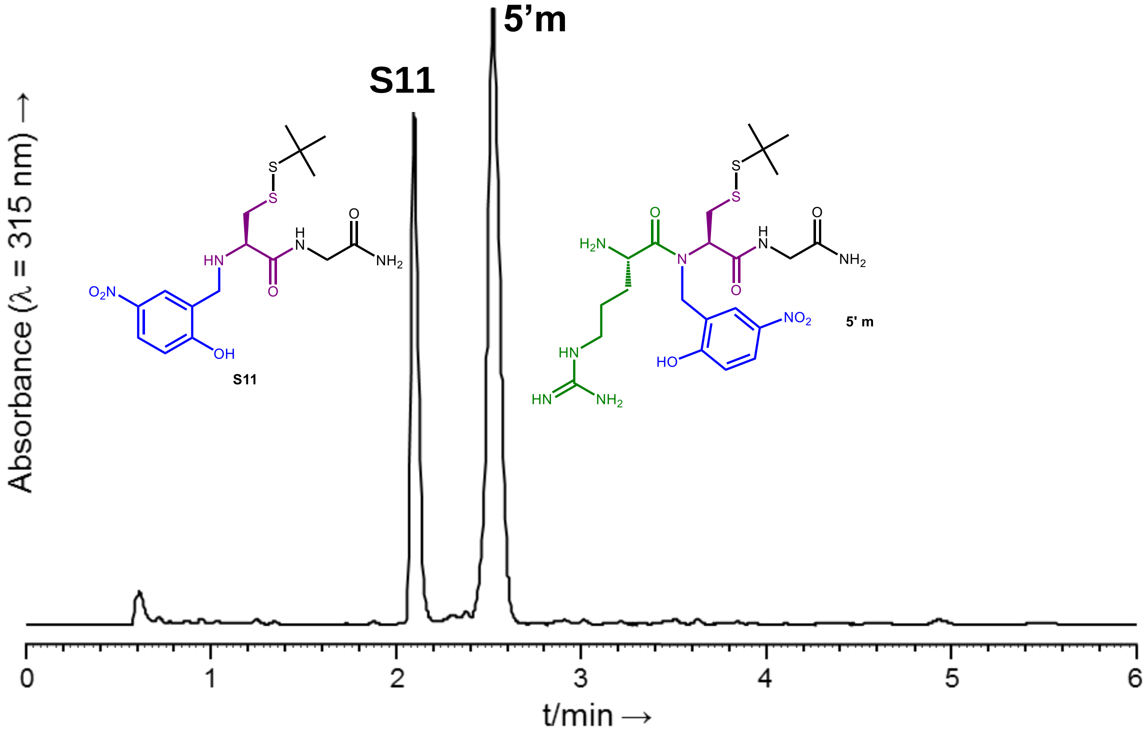

Supplementary figure S32: HPLC trace of crude **5’m**.

Asn - 5'g:

ESI-HRMS (*m/z*): $[MH]^+$ calcd for $C_{20}H_{31}N_6O_7S_2$: 531.1696, found: 531.1690.

HPLC: retention time: 2.97 min (Chromolith, gradient: 20-50 % B/A over 5 min).

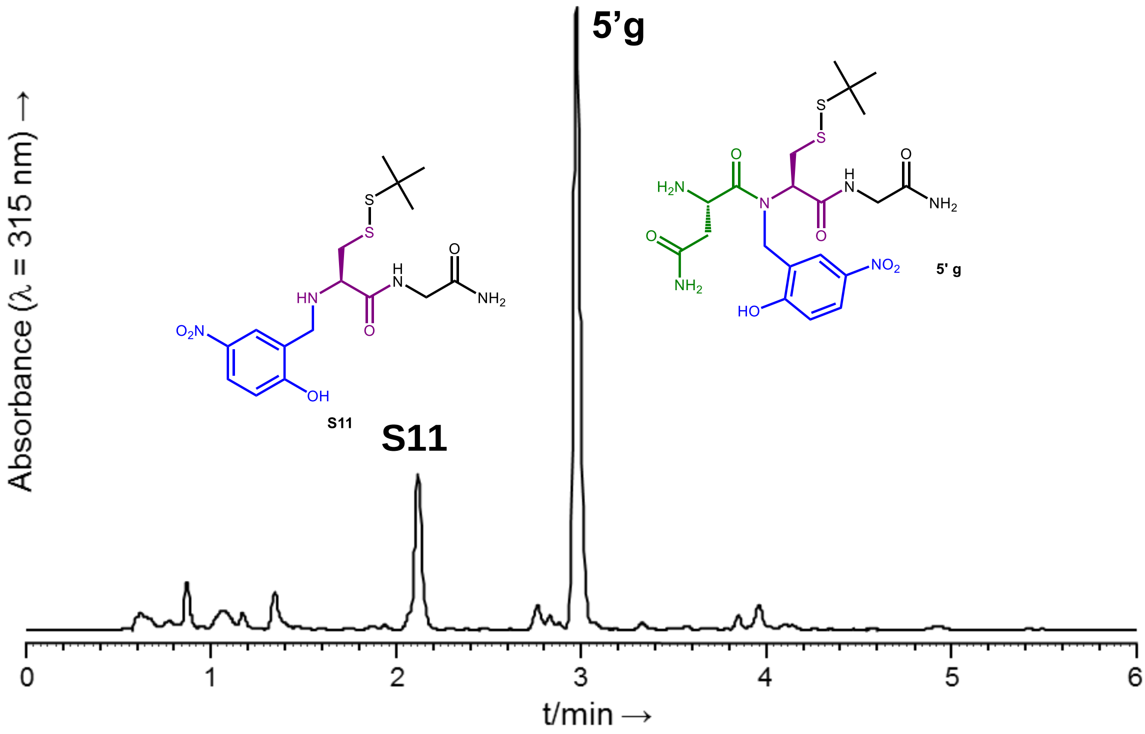

Supplementary figure S33: HPLC trace of crude **5'g**.

Asp - 5'b:

ESI-HRMS (*m/z*): $[MH]^+$ calcd for $C_{20}H_{30}N_5O_8S_2$: 532.1536, found: 532.1528.

HPLC: retention time: 3.48 min (Chromolith, gradient: 20-50 % B/A over 5 min).

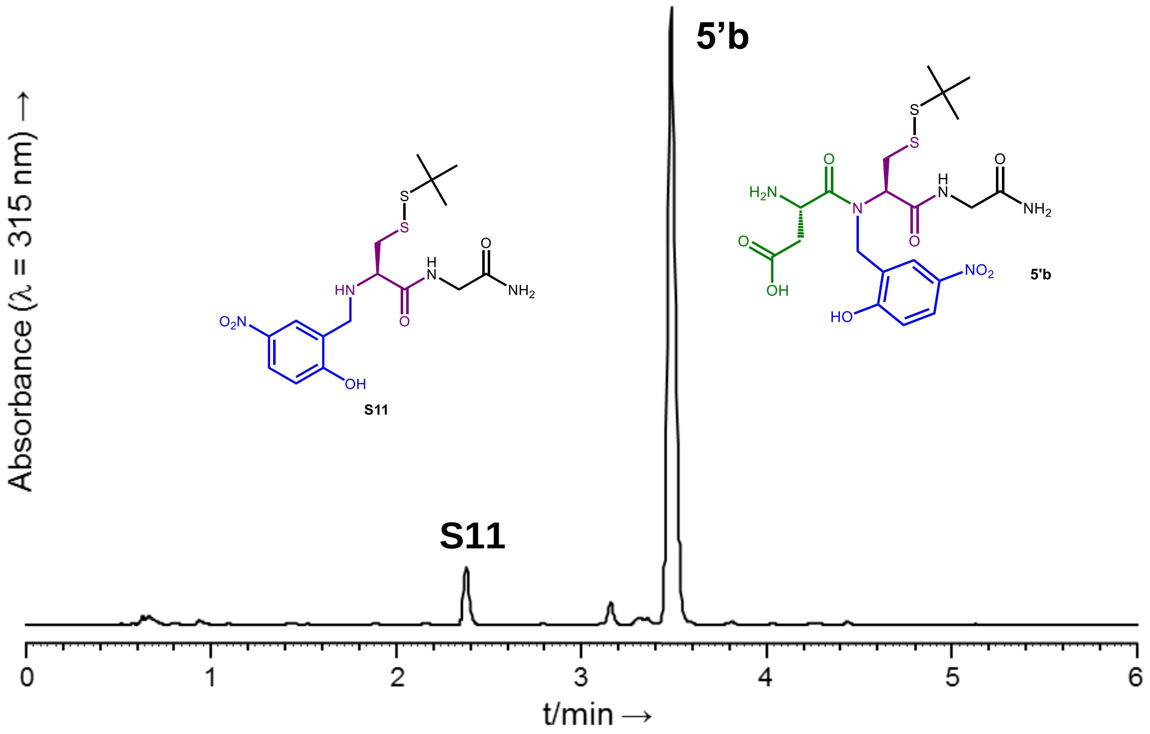

Supplementary figure S34: HPLC trace of crude **5'b**.

Cys - 5'e:

ESI-HRMS (*m/z*): $[MH]^+$ calcd for $C_{19}H_{30}N_5O_6S_3$: 520.1358, found: 520.1351.
HPLC: retention time: 4.07 min (Chromolith, gradient: 20-50 % B/A over 5 min).

In the case of compound **5'e**, a minor peak was observed showing the same *m/z* as expected for compound **5'e**. It was attributed to epimerization of Fmoc-Cys(Trt) during coupling, yielding compound **S12** after TFA cleavage (ratio: L-Cys / D-Cys 93:7).

D-Cys - S12:

ESI-HRMS (*m/z*): $[MH]^+$ calcd for $C_{19}H_{30}N_5O_6S_3$: 520.1358, found: 520.1353.
HPLC: retention time: 3.41 min (Chromolith, gradient: 20-50 % B/A over 5 min).

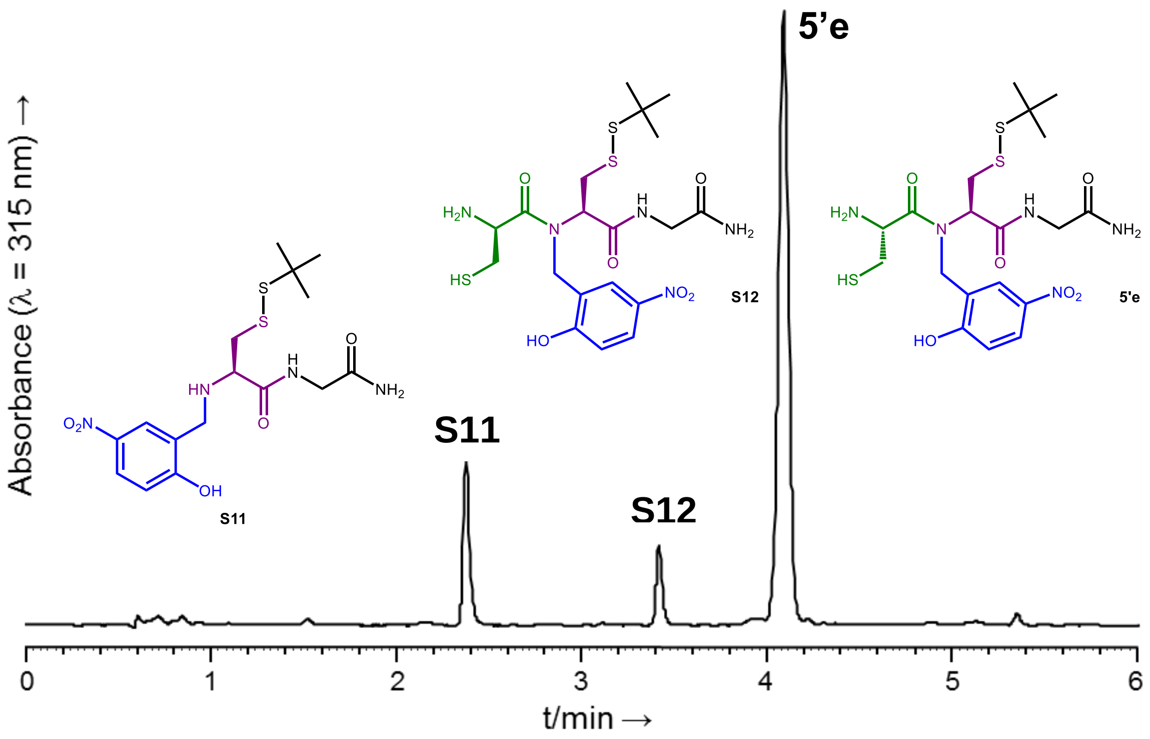

Supplementary figure S35: HPLC trace of crude **5'e**.

Gln - 5’j:

ESI-HRMS (*m/z*): $[MH]^+$ calcd for $C_{21}H_{33}N_6O_7S_2$: 545.1852, found: 545.1847.
HPLC: retention time: 3.27 min (Chromolith, gradient: 20-50 % B/A over 5 min).

In the case of compound **5’j**, concomitant formation of pyroglutamate (**S13**) was observed during the TFA treatment.

PyroGlu - S13:

ESI-HRMS (*m/z*): $[MH]^+$ calcd for $C_{21}H_{30}N_5O_7S_2$: 528.1587, found: 528.1579.
HPLC: retention time: 4.34 min (Chromolith, gradient: 20-50 % B/A over 5 min).

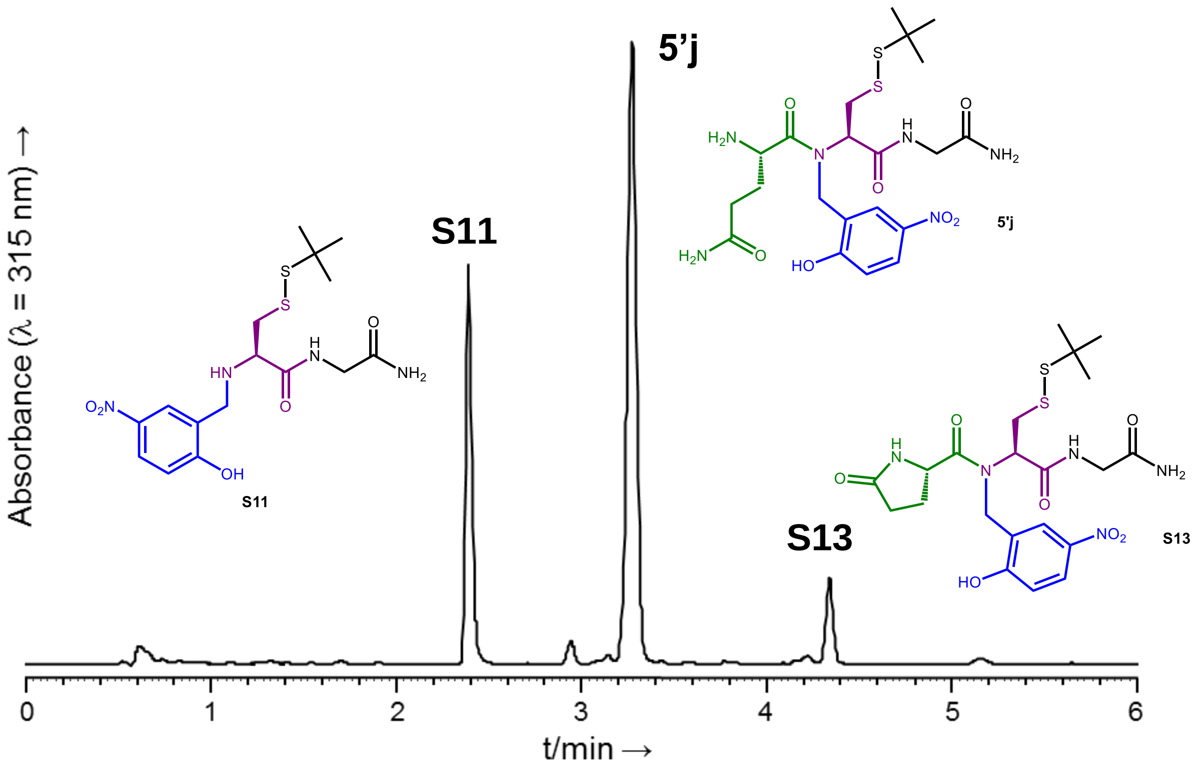

Supplementary figure S36: HPLC trace of crude **5’j**.

Glu 5'd:

ESI-HRMS (*m/z*): $[MH]^+$ calcd for $C_{21}H_{32}N_5O_8S_2$: 546.1692, found: 546.1684.

HPLC: retention time: 3.55 min (Chromolith, gradient: 20-50 % B/A over 5 min).

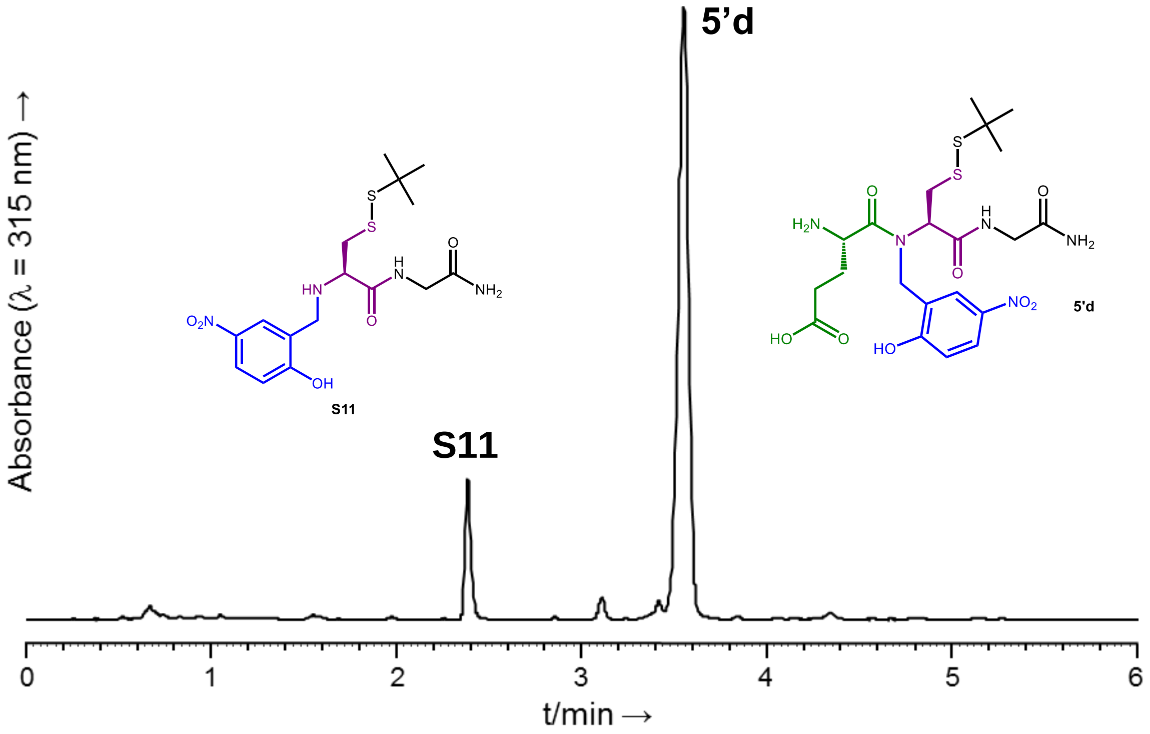

Supplementary figure S37: HPLC trace of crude **5'd**.

Gly - 5'a:

ESI-HRMS (*m/z*): $[MH]^+$ calcd for $C_{18}H_{28}N_5O_6S_2$: 474.1481, found: 474.1476.

HPLC: retention time: 3.01 min (Chromolith, gradient: 20-50 % B/A over 5 min).

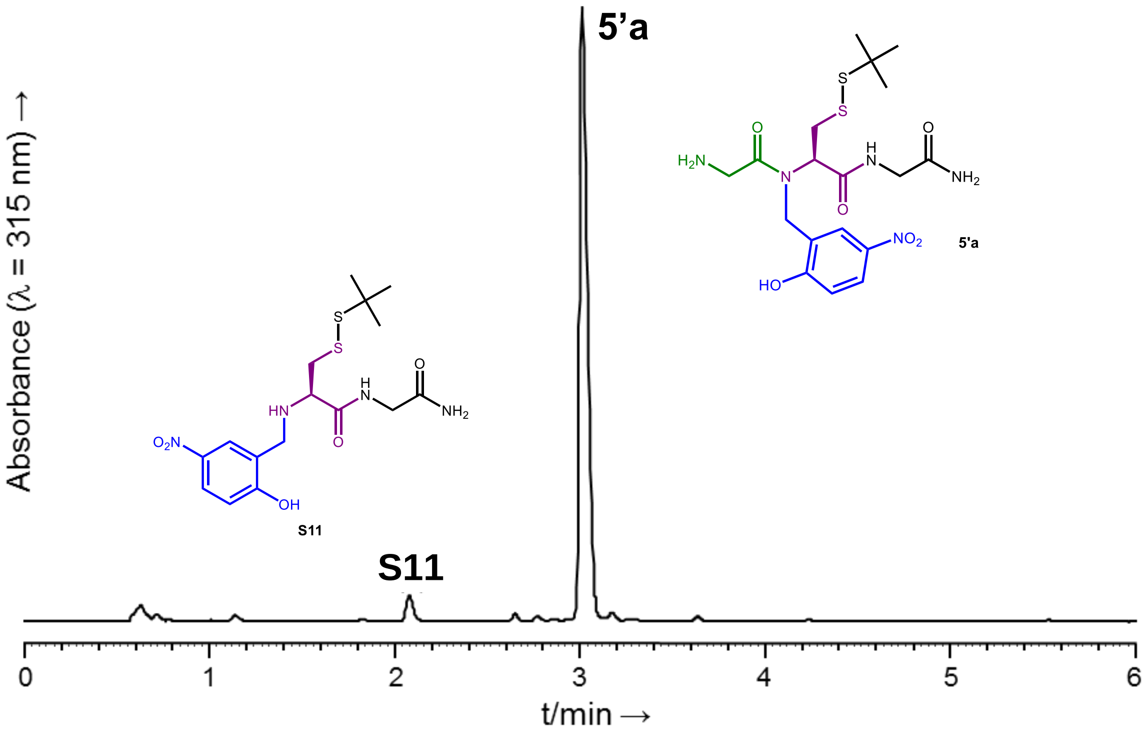

Supplementary figure S38: HPLC trace of crude **5'a**.

His - 5’h:

ESI-HRMS (*m/z*): $[MH]^+$ calcd for $C_{22}H_{32}N_7O_6S_2$: 554.1855, found: 554.1848.

HPLC: retention time: 2.80 min (Chromolith, gradient: 20-50 % B/A over 5 min).

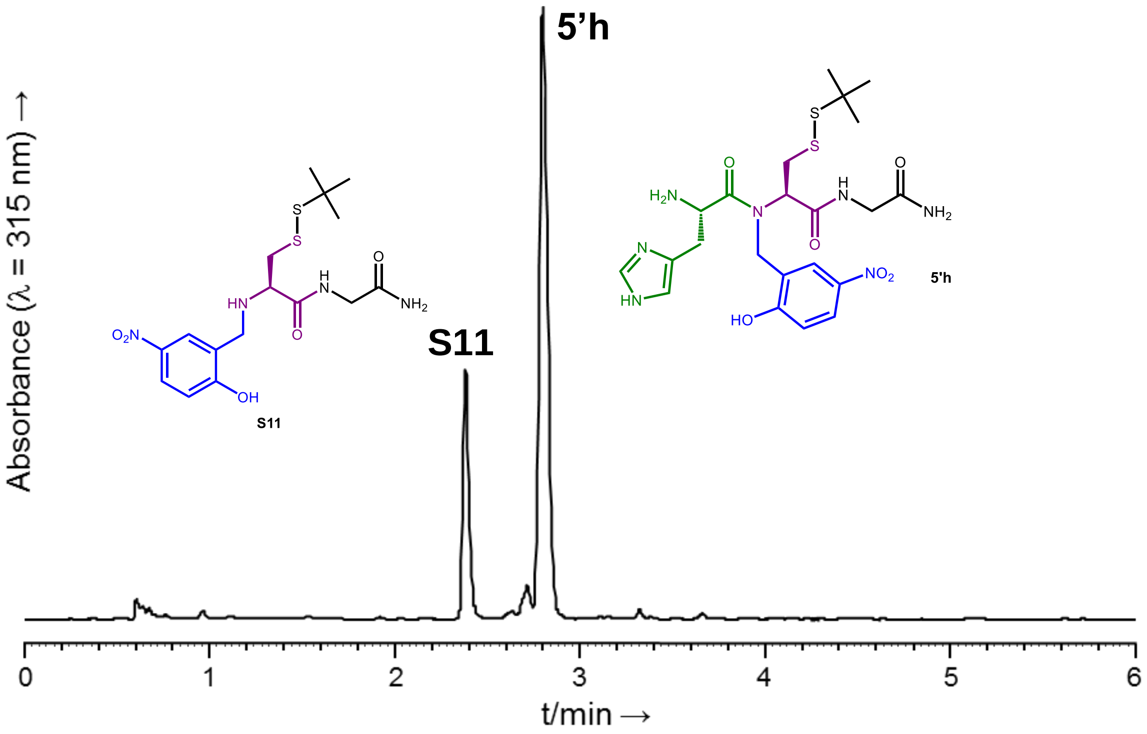

Supplementary figure S39: HPLC trace of crude **5’h**.

Ile - 5’t:

ESI-HRMS (*m/z*): $[MH]^+$ calcd for $C_{22}H_{36}N_5O_6S_2$: 530.2107, found: 530.2104.

HPLC: retention time: 4.72 min (Chromolith, gradient: 20-50 % B/A over 5 min).

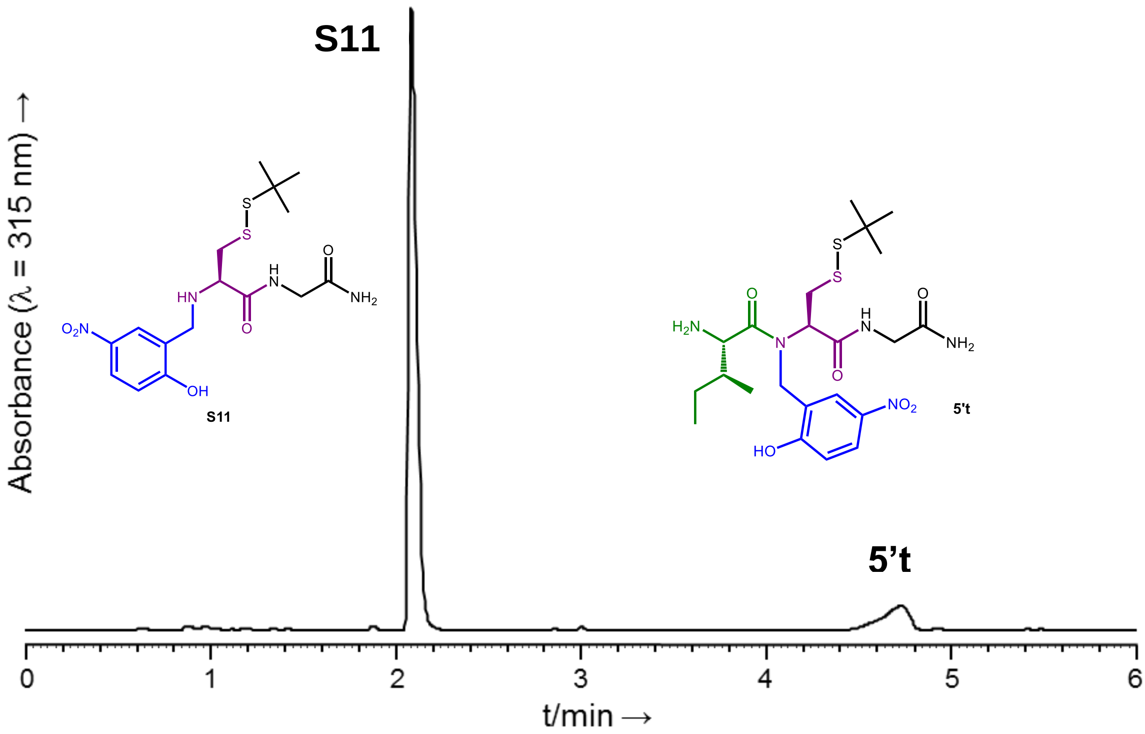

Supplementary figure S40: HPLC trace of crude **5’t**.

Fmoc-Ile-OH (0.05 mmol, 10 equiv.) was coupled using a Biotage Initiator+ SP Wave, on 5 µmol of resin **4** using HCTU (19.6 mg, 0.0476 mmol, 9.5 equiv.) and *i*Pr_2NEt (17.4 µL, 0.1 mmol, 20 equiv.) in NMP (0.6 mL) under microwave heating at 70°C for 30 min. After that the peptide resin was treated with 20 % piperidine in NMP (3 × 3 min); finally, resin was cleaved using 1mL of a TFA/H_2O/*i*Pr_3SiH (93:5:2) solution for 2 h. After evaporation of TFA, the sample was analyzed by HPLC; the *N*-acylation yield was quantified by integration at 315 nm, not taking into account eventual differences in molar extinction coefficient of the products.

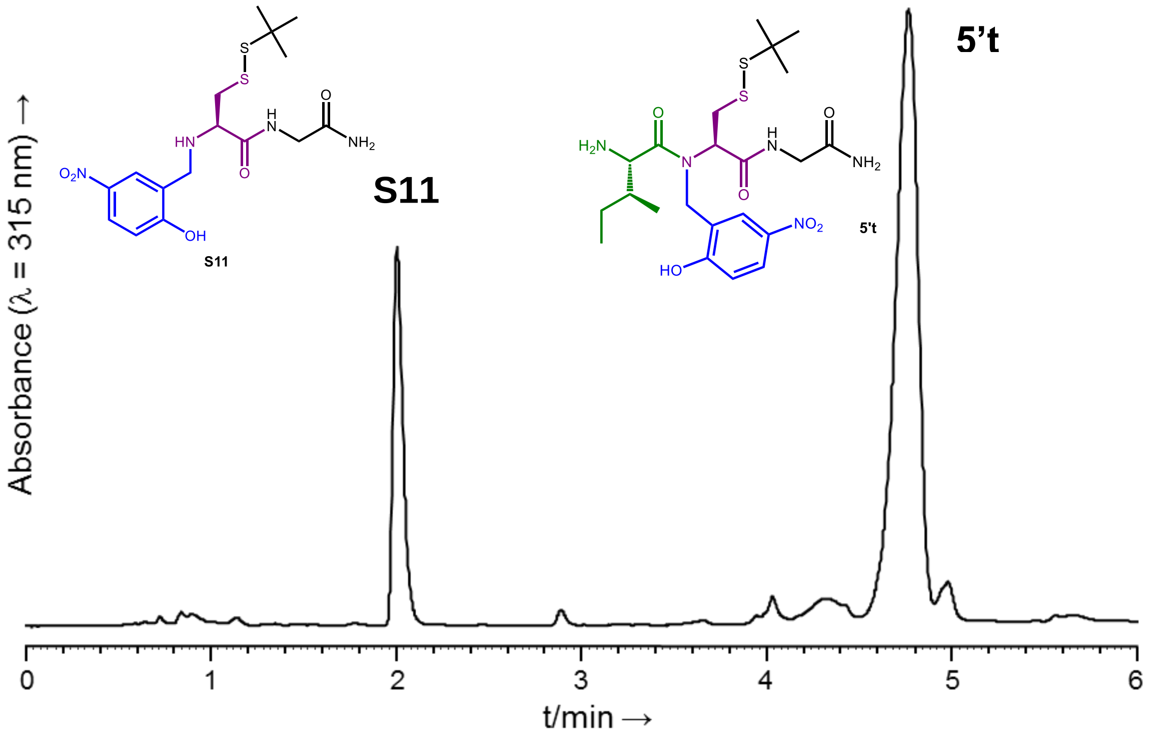

Supplementary figure S41: HPLC trace of crude **5’t** after microwave-assisted coupling.

Leu - 5’k:

ESI-HRMS (*m/z*): [MH]$^+$ calcd for $C_{22}H_{36}N_5O_6S_2$: 530.2107, found: 530.2106.

HPLC: retention time: 4.66 min (Chromolith, gradient: 20-50 % B/A over 5 min).

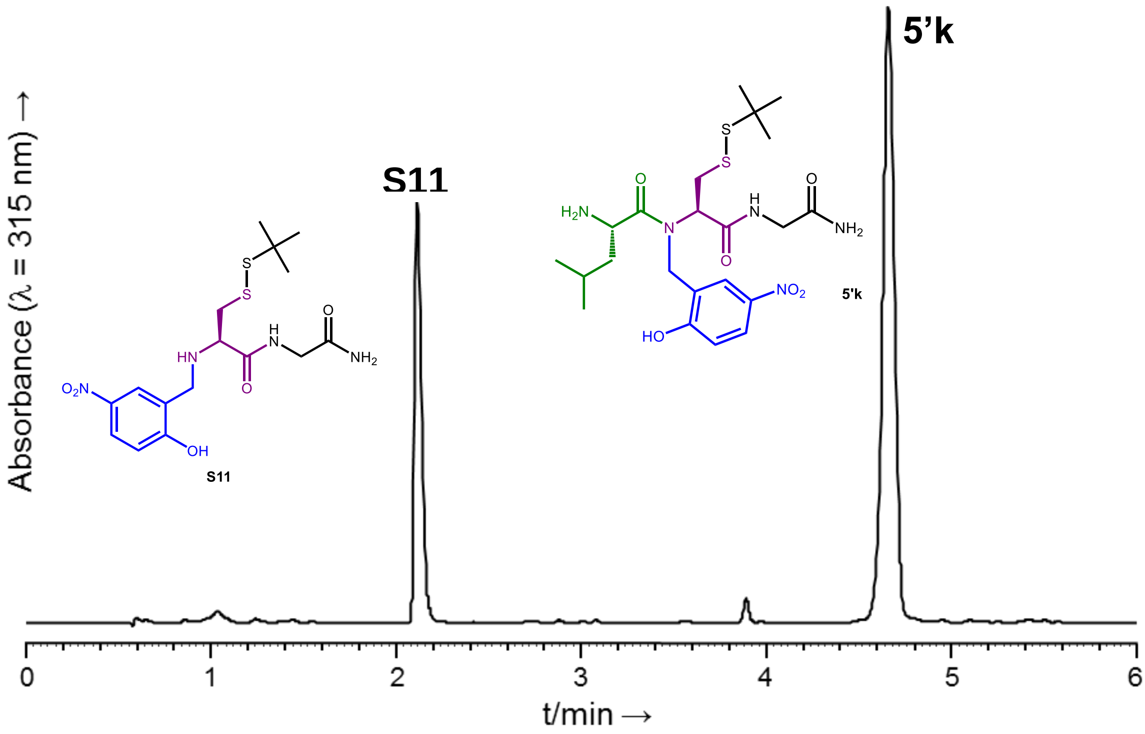

Supplementary figure S42: HPLC trace of crude **5’k**.

Lys - 5’p:

ESI-HRMS (*m/z*): $[MH]^+$ calcd for $C_{22}H_{37}N_6O_6S_2$: 545.2216, found: 545.2210.
HPLC: retention time: 2.40 min (Chromolith, gradient: 20-50 % B/A over 5 min).

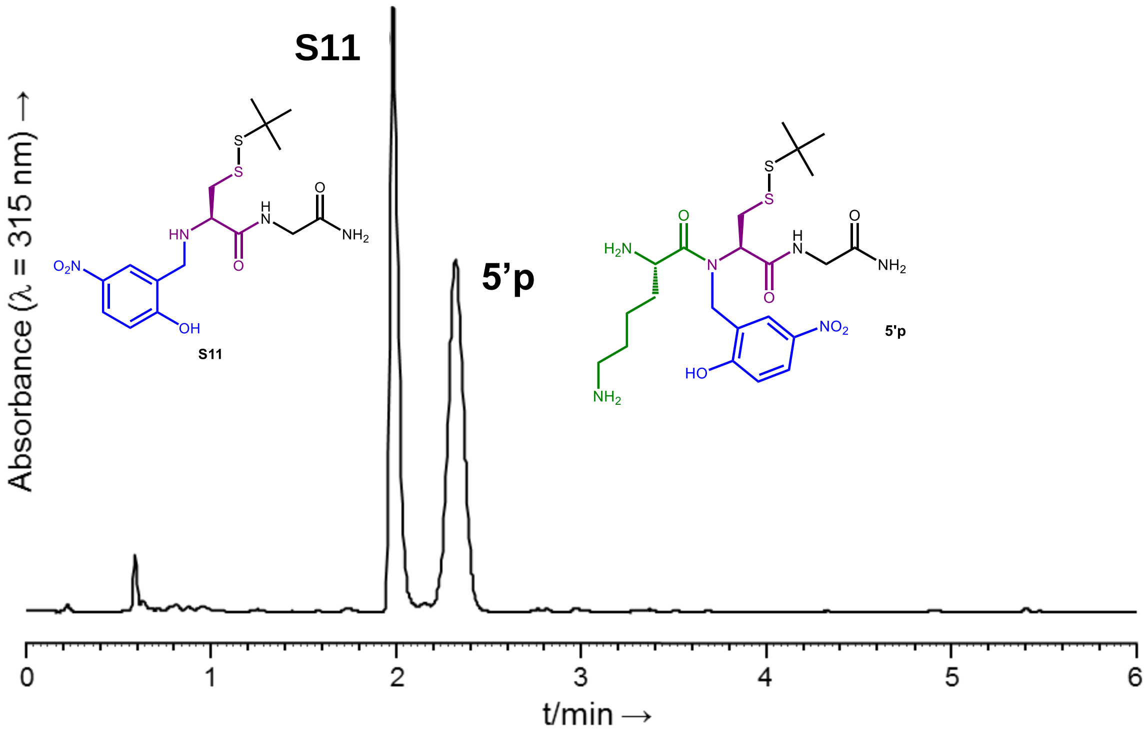

Supplementary figure S43: HPLC trace of crude **5’p**.

Met - 5’f:

ESI-HRMS (*m/z*): $[MH]^+$ calcd for $C_{21}H_{34}N_5O_6S_3$: 548.1671, found: 548.1665.

HPLC: retention time: 4.56 min (Chromolith, gradient: 20-50 % B/A over 5 min).

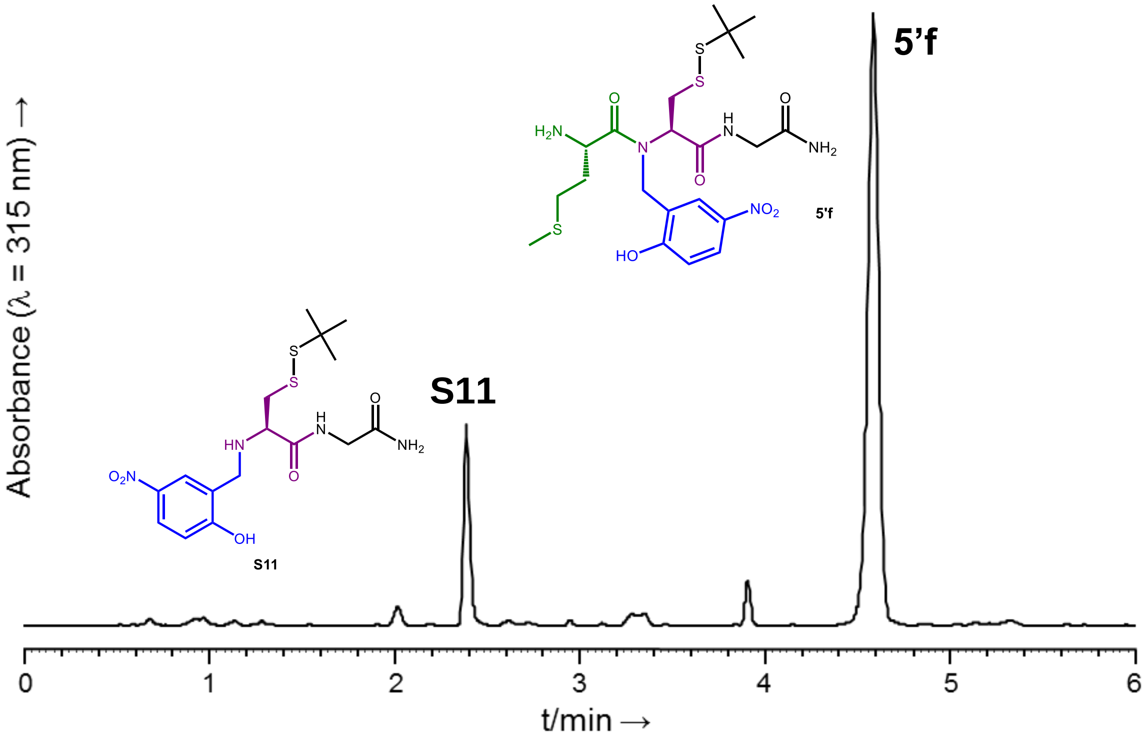

Supplementary figure S44: HPLC trace of crude **5’f**.

Phe - 5’i:

ESI-HRMS (*m/z*): $[MH]^+$ calcd for $C_{25}H_{34}N_5O_6S_2$: 564.1950, found: 564.1942.

HPLC: retention time: 5.07 min (Chromolith, gradient: 20-50 % B/A over 5 min).

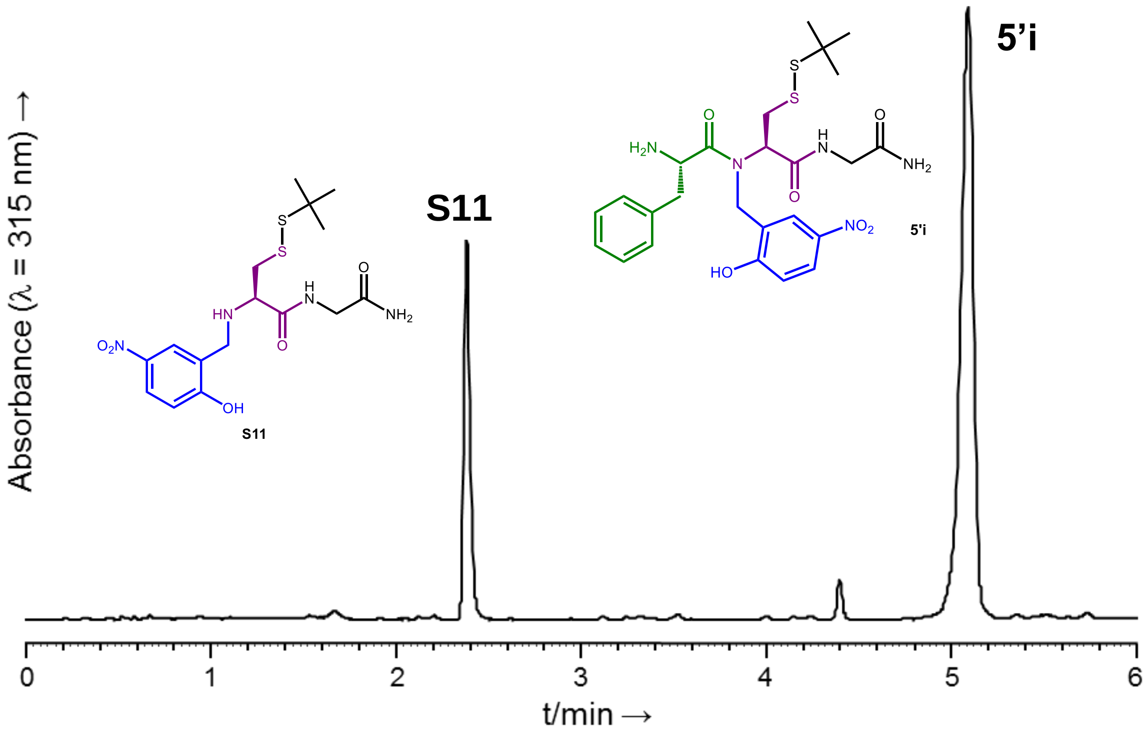

Supplementary figure S45: HPLC trace of crude **5’i**.

Pro - 5'r:

ESI-HRMS (*m/z*): $[MH]^+$ calcd for $C_{21}H_{32}N_5O_6S_2$: 514.1794, found: 514.1788.

HPLC: retention time: 3.86 min (Chromolith, gradient: 20-50 % B/A over 5 min).

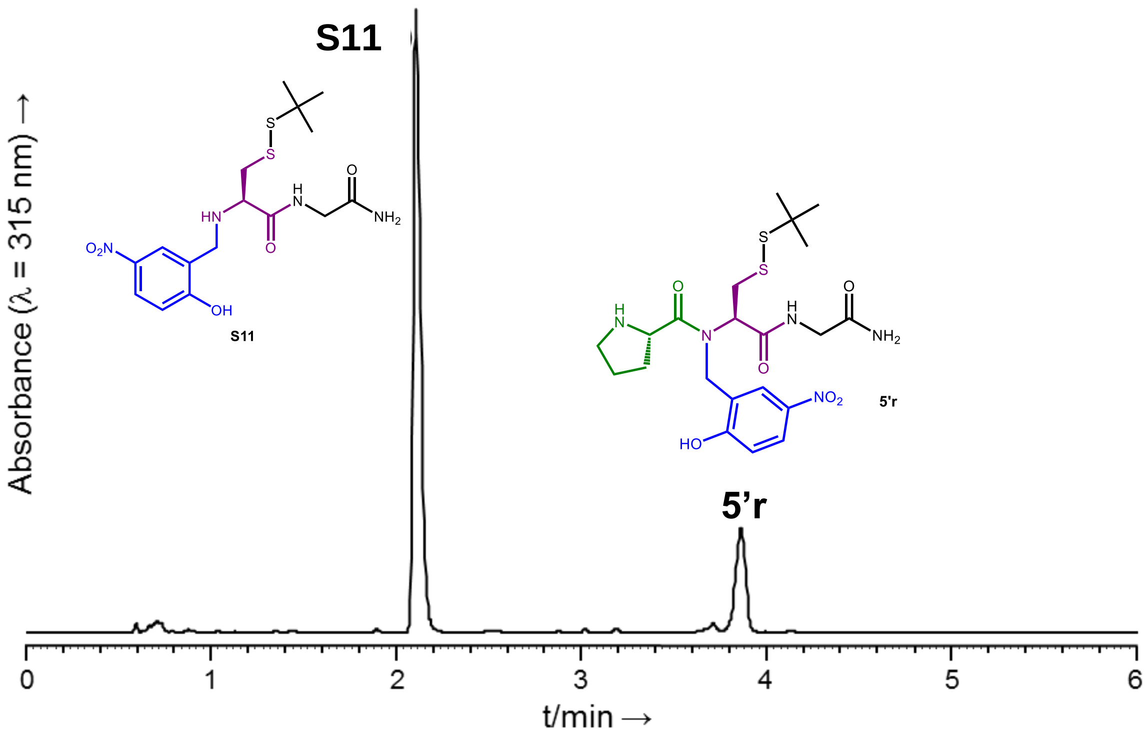

Supplementary figure S46: HPLC trace of crude **5'r**.

Ser - 5'o:

ESI-HRMS (*m/z*): $[MH]^+$ calcd for $C_{19}H_{30}N_5O_7S_2$: 504.1587, found: 504.1580.

HPLC: retention time: 3.13 min (Chromolith, gradient: 20-50 % B/A over 5 min).

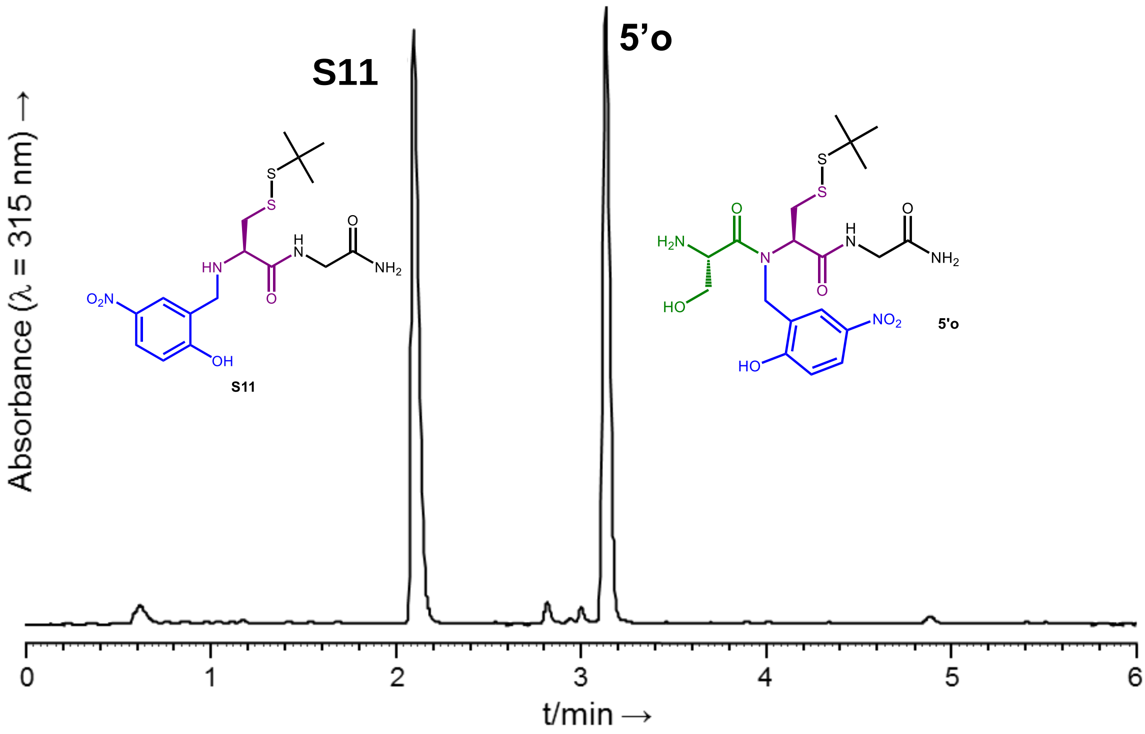

Supplementary figure S47: HPLC trace of crude **5'o**.

Thr - 5’q:

ESI-HRMS (*m/z*): $[MH]^+$ calcd for $C_{20}H_{32}N_5O_7S_2$: 518.1743, found: 518.1736.

HPLC: retention time: 3.46 min (Chromolith, gradient: 20-50 % B/A over 5 min).

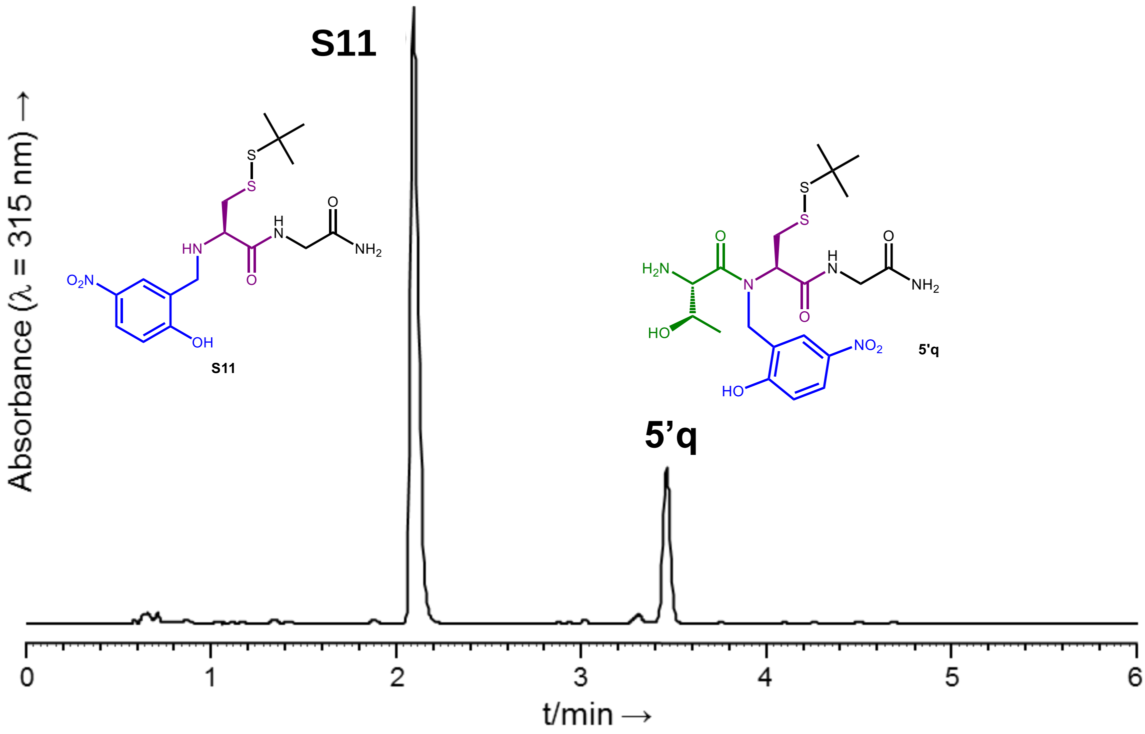

Supplementary figure S48: HPLC trace of crude **5’q**.

Trp - 5'l:

ESI-HRMS (*m/z*): $[MH]^+$ calcd for $C_{27}H_{35}N_6O_6S_2$: 603.2059, found: 603.2052.

HPLC: retention time: 5.00 min (Chromolith, gradient: 20-50 % B/A over 5 min).

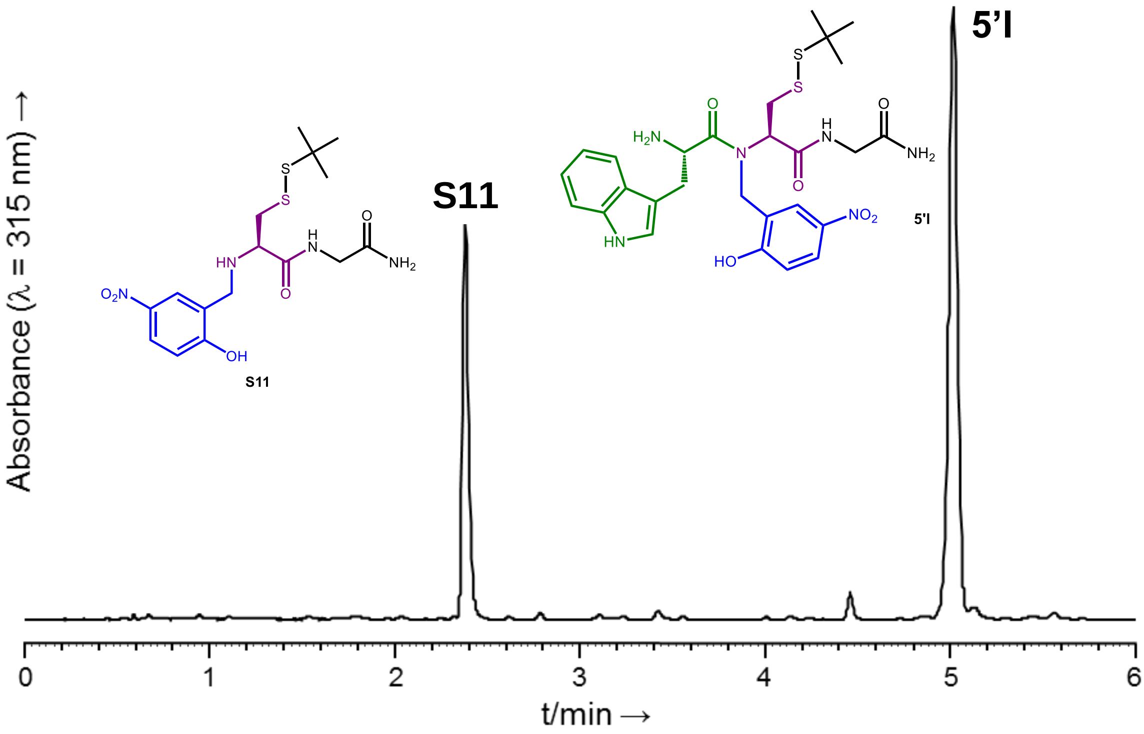

Supplementary figure S49: HPLC trace of crude **5'l**.

Tyr - 5’n:

ESI-HRMS (*m/z*): $[MH]^+$ calcd for $C_{25}H_{34}N_5O_7S_2$: 580.1900, found: 580.1892.

HPLC: retention time: 4.18 min (Chromolith, gradient: 20-50 % B/A over 5 min).

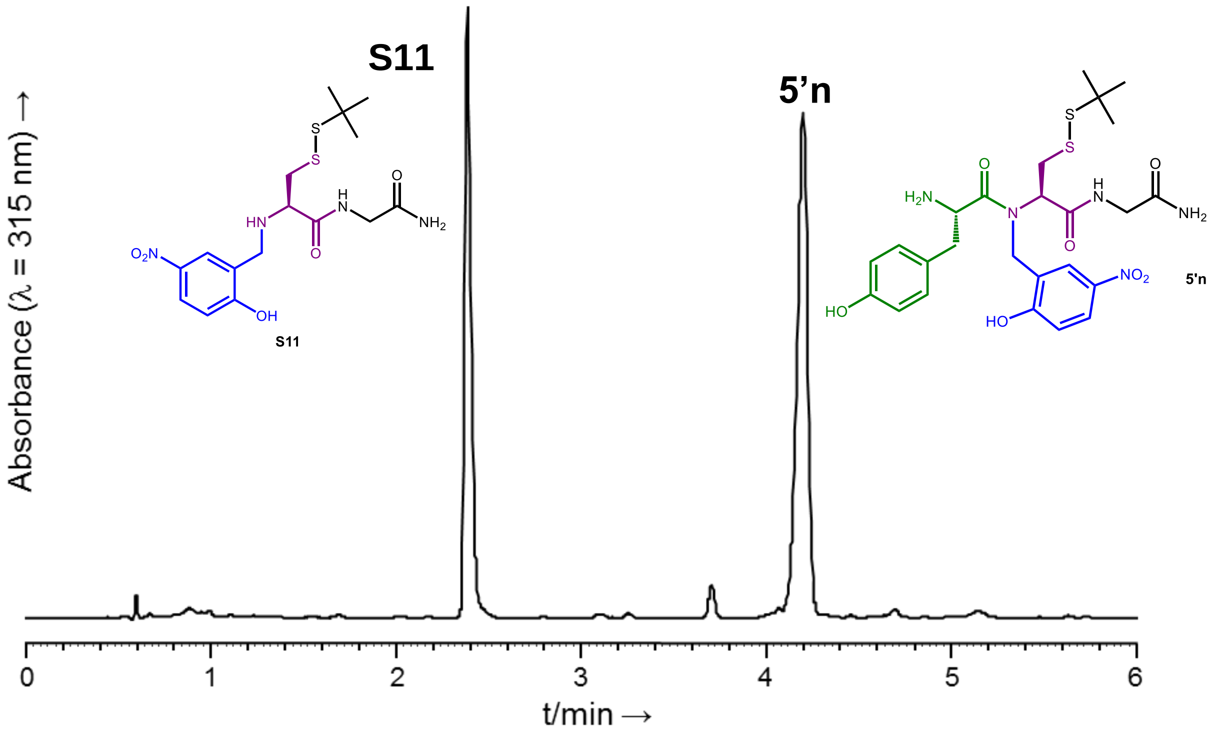

Supplementary figure S50: HPLC trace of crude **5’n**.

Val - 5’s:

ESI-HRMS (*m/z*): $[MH]^+$ calcd for $C_{21}H_{34}N_5O_6S_2$: 516.1950, found: 516.1947.

HPLC: retention time: 4.33 min (Chromolith, gradient: 20-50 % B/A over 5 min).

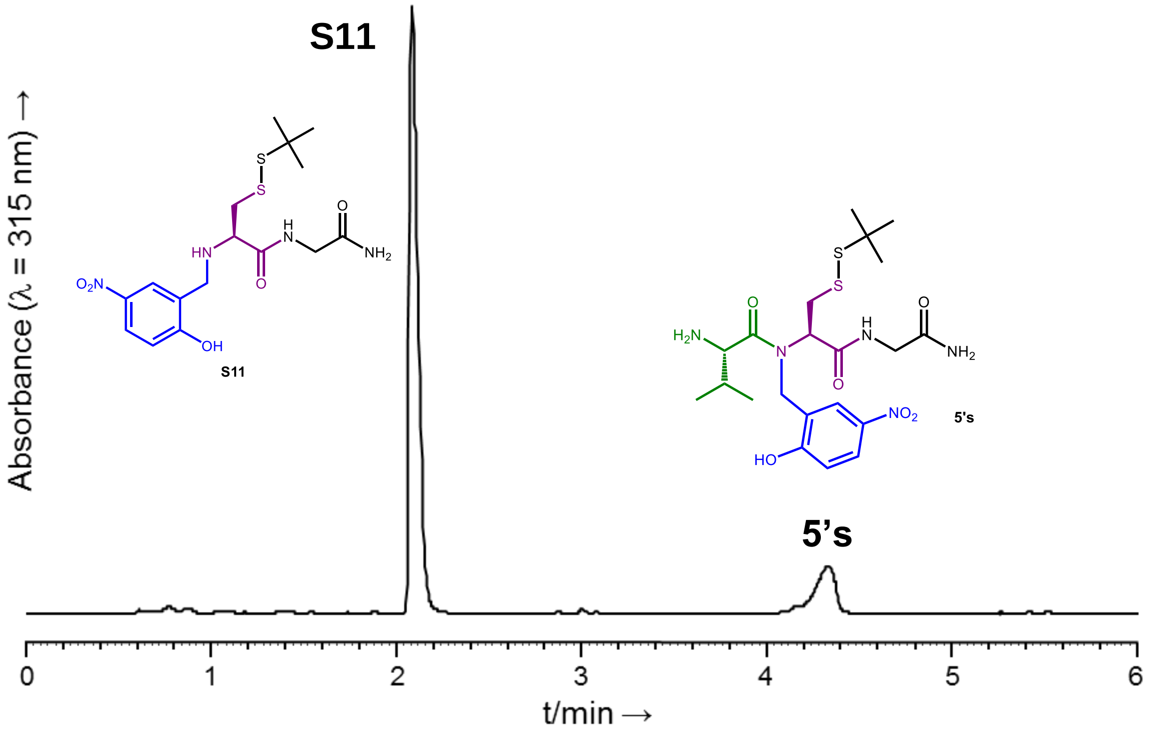

Supplementary figure S51: HPLC trace of crude **5’s**.

Fmoc-Val-OH (0.05 mmol, 10 equiv.) was coupled using a Biotage Initiator+ SP Wave, on 5 µmol of resin **4** using HCTU (19.6 mg, 0.0476 mmol, 9.5 equiv.) and *i*Pr_2NEt (17.4 µL, 0.1 mmol, 20 equiv.) in NMP (0.6 mL) under microwave heating at 70°C for 30 min. After that the peptide resin was treated with 20 % piperidine in NMP (3 × 3 min); finally, resin was cleaved using 1mL of a TFA/H_2O/*i*Pr_3SiH (93:5:2) solution for 2 h. After evaporation of TFA, the sample was analyzed by HPLC; the *N*-acylation yield was quantified by integration at 315 nm, not taking into account eventual differences in molar extinction coefficient of the products.

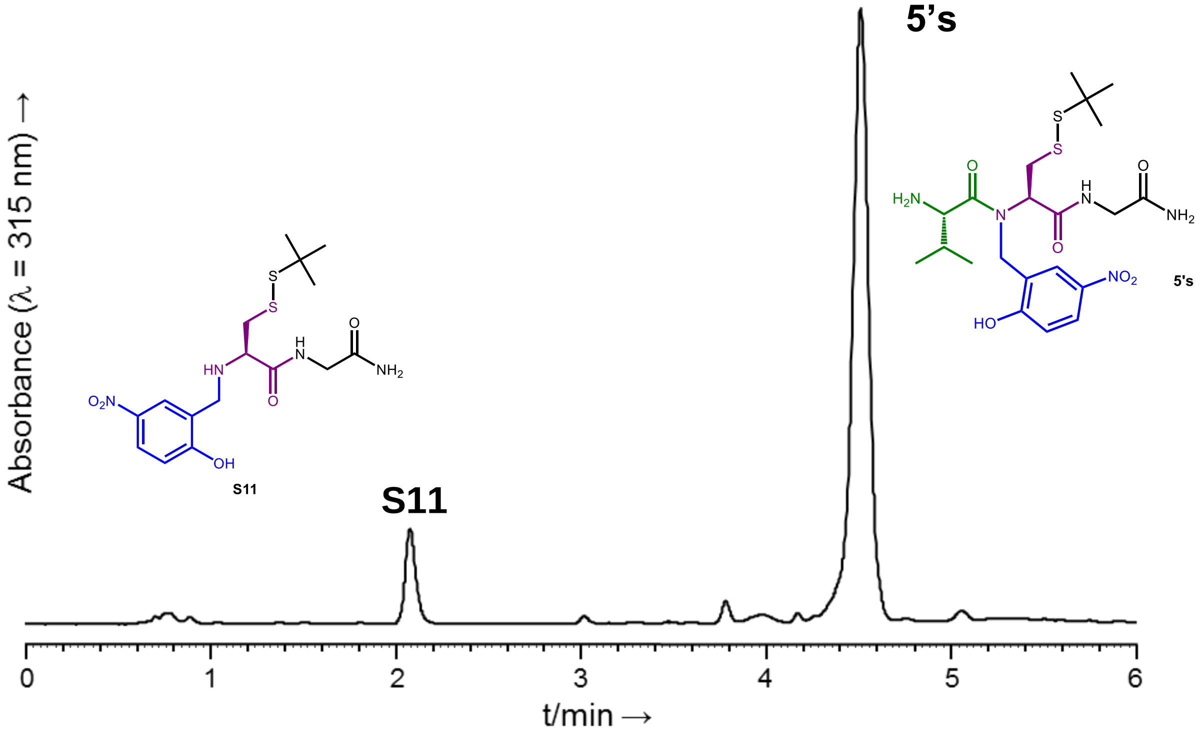

Supplementary figure S52: HPLC trace of crude **5's** after microwave-assisted coupling.

9- Kinetics studies of the NCL with model Ac-LYRAA(Hnb)C peptides

9a- Synthesis of the model cysteinyl peptide 6

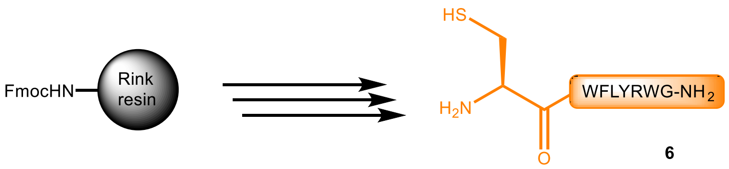

Supplementary scheme S16: Synthesis of the model cysteinyl peptide **6**.

Peptide **6** was obtained through automated SPPS (protocol p S3) starting from Fmoc-Rink polystyrene resin (130 mg, 0.79 mmol/g, 0.1 mmol). The peptide-resin

was cleaved (protocol p S3) and **6** was purified by HPLC (Nucleosil, gradient: 32-39 % B/A over 10 min).

6:

MALDI-TOF (*m/z*): $[MH]^+$ calcd for $C_{57}H_{73}N_{14}O_9S$: 1129.53, found: 1129.53.
HPLC: retention time: 26.24 min (Nucleosil, gradient: 5-50 % B/A over 30 min).

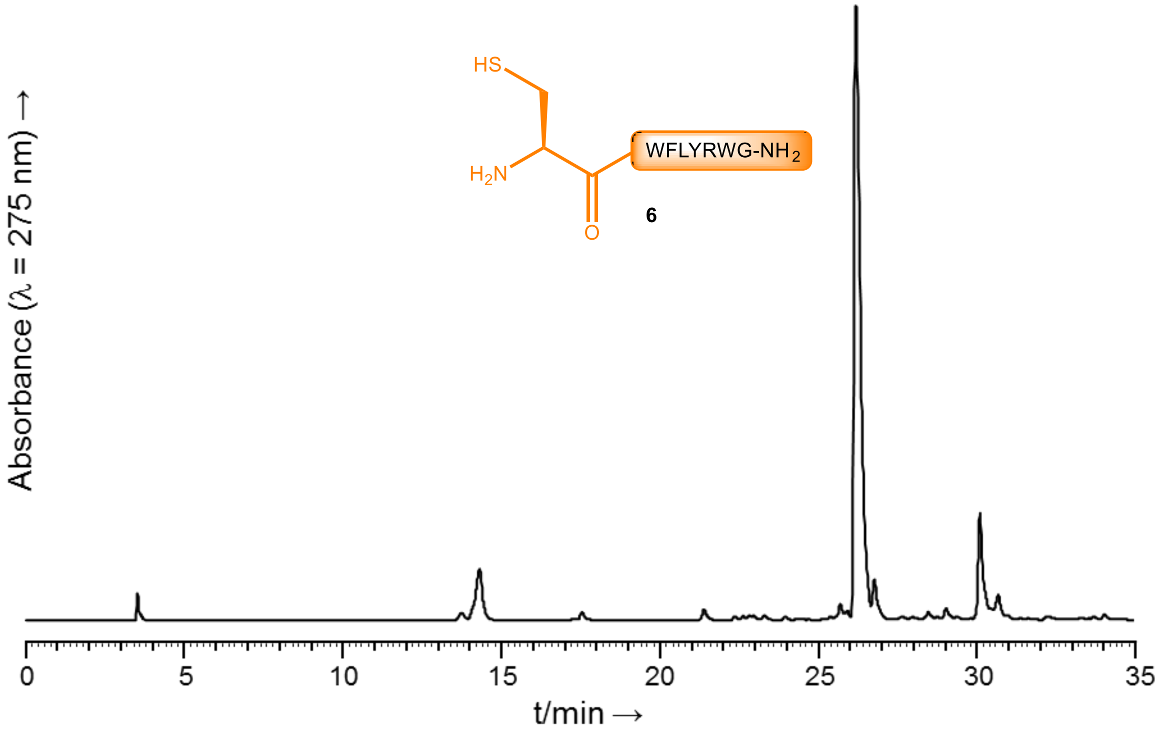

Supplementary figure S53: HPLC trace of crude **6**.

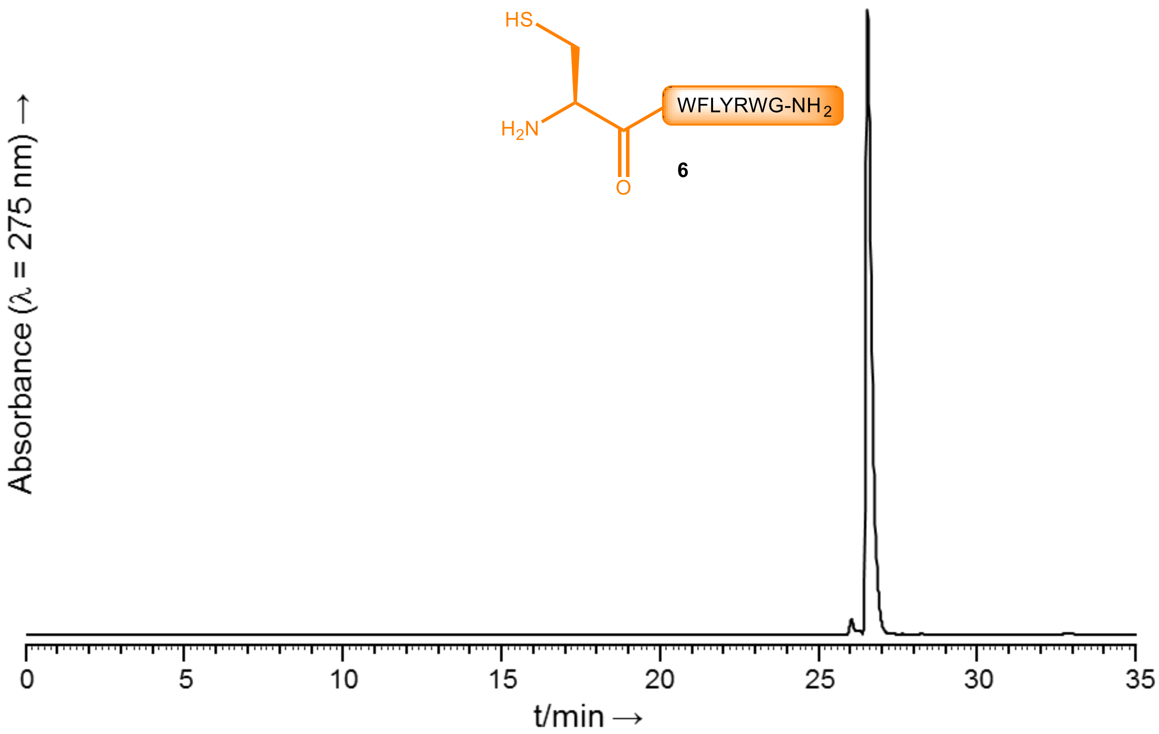

Supplementary figure S54: HPLC trace of pure **6**.

9b- Influence of the nature of the cysteine C-terminus

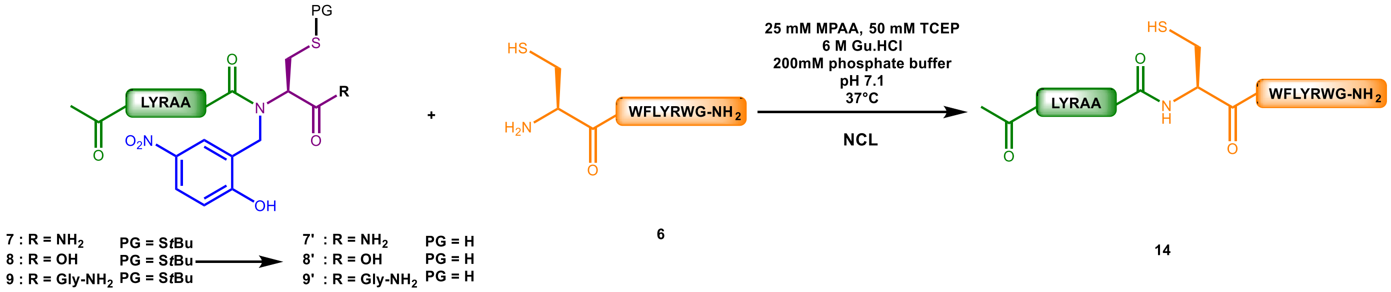

Supplementary scheme S17: Native chemical ligations with **7**, **8** or **9**.

Ligation with **7**:

500 µL of a degassed 0.2 M pH 7.1 sodium phosphate buffer containing 25 mM MPAA, 50 mM TCEP and 6 M guanidine hydrochloride were added to 1.2 mg of the peptide **7** (final concentration 2 mM) and 0.7 mg peptide **6** (final concentration 1 mM) under argon. The ligation was carried out at 37°C and monitored by RP-HPLC (Chromolith, gradient: 20-70 % B/A over 6 min). For this, aliquots of 2 µL were diluted in 108 µL of 1.5 % TFA in water and 100 µL were injected in HPLC.

Ligation with **8**:

500 µL of a degassed 0.2 M pH 7.1 sodium phosphate buffer containing 25 mM MPAA, 50 mM TCEP and 6 M guanidine hydrochloride were added to 1.05 mg of the

peptide **8** (final concentration 2 mM) and 0.69 mg peptide **6** (final concentration 1 mM) under argon. The ligation was carried out at 37°C and monitored by RP-HPLC (Chromolith, gradient: 20-70 % B/A over 6 min). For this, aliquots of 2 µL were diluted in 108 µL of 1.5 % TFA in water and 100 µL were injected in HPLC.

Ligation with **9**:

500 µL of a degassed 0.2 M pH 7.1 sodium phosphate buffer containing 25 mM MPAA, 50 mM TCEP and 6 M guanidine hydrochloride were added to 1.3 mg of the peptide **9** (final concentration 2 mM) and 0.7 mg peptide **6** (final concentration 1 mM) under argon. The ligation was carried out at 37°C and monitored by RP-HPLC (Chromolith, gradient: 20-70 % B/A over 6 min). For this, aliquots of 2 µL were diluted in 108 µL of 1.5 % TFA in water and 100 µL were injected in HPLC.

Ligation product 14:

ESI-HRMS (*m/z*): $[MH]^+$ calcd for $C_{86}H_{117}N_{22}O_{16}S$: 1745.8739, found: 1745.8773.
HPLC: retention time: 4.46 min (Chromolith, gradient: 20-70 % B/A over 6 min).

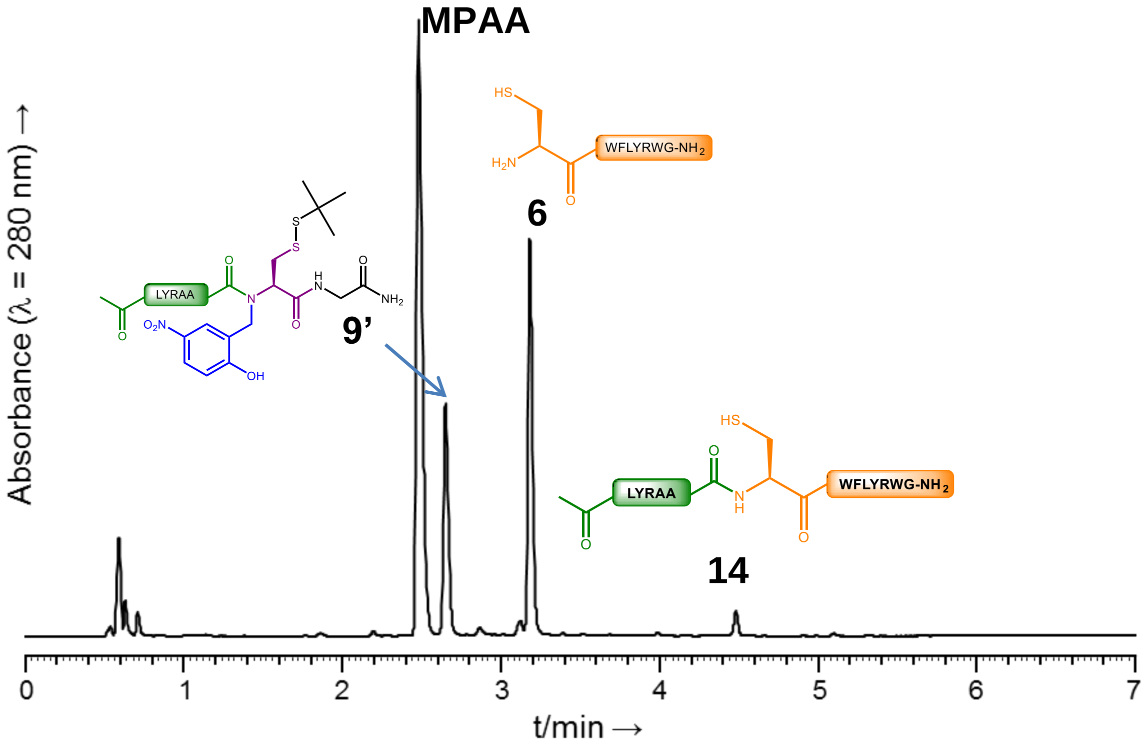

Supplementary figure S55: NCL at 15 min using **9**.

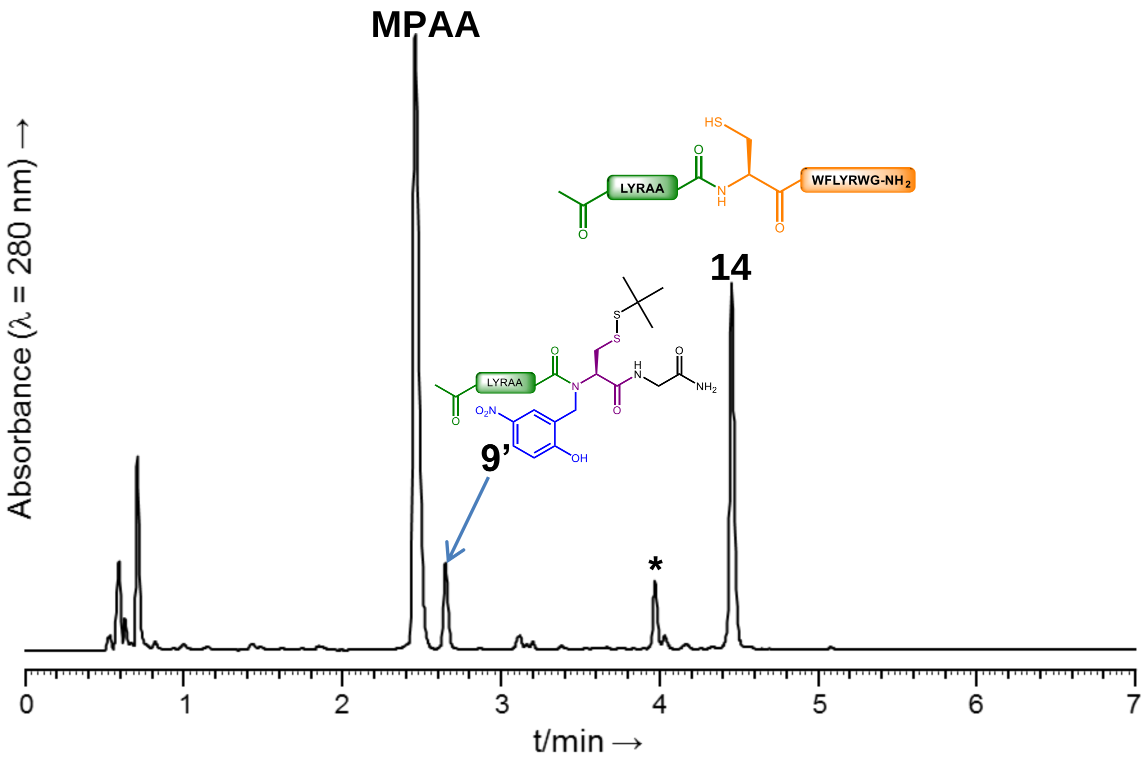

Supplementary figure S56: NCL after 24h using **9**. *: non peptidic compound

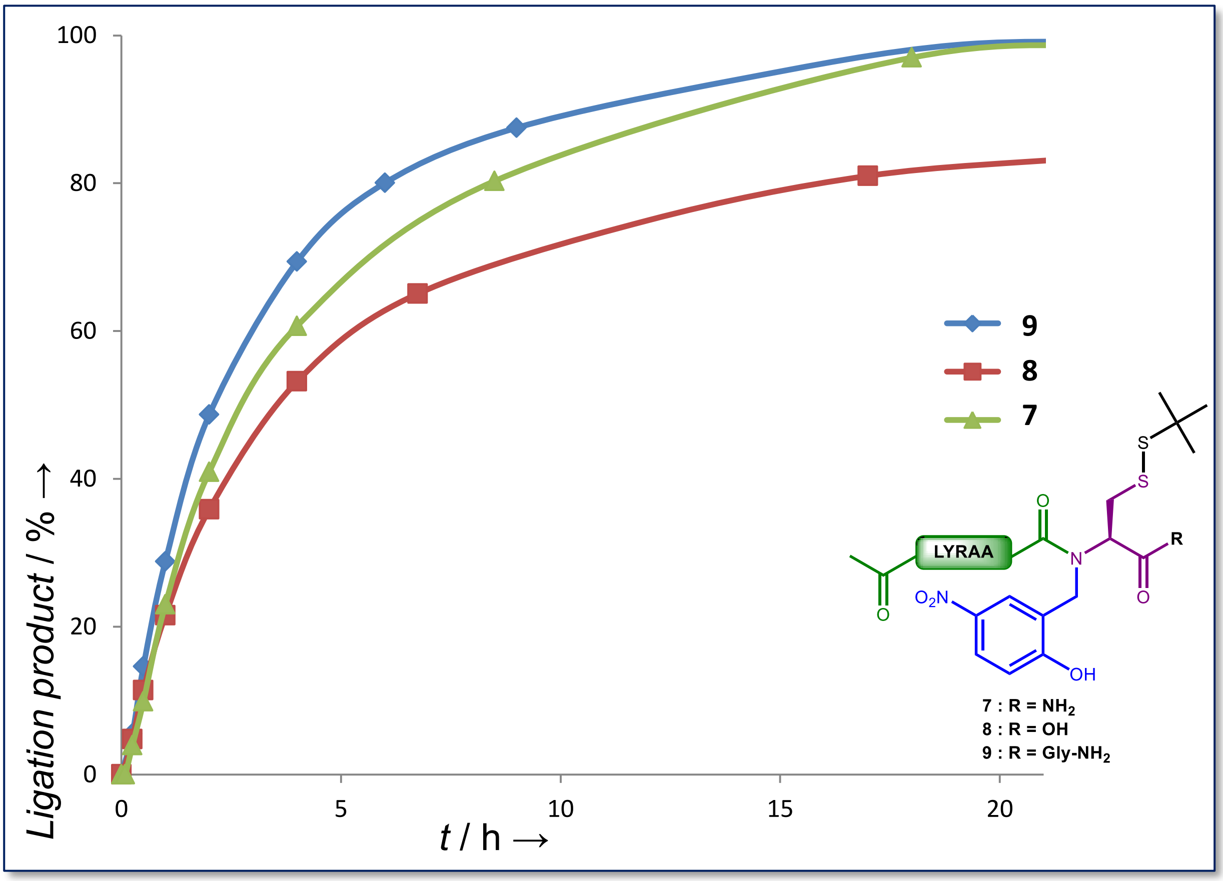

Supplementary figure S57: Ligation kinetics observed using **7**, **8** or **9**.

9c- Study of the hydrolysis of the thioester precursor **9** and epimerization at the ligation site during a typical NCL with **9**

In order to quantify the hydrolysis of the thioester and the epimerization at the ligation site during a classical NCL with **6** and **9** (page S60), HPLC standards **S14** and **15** were synthesized.

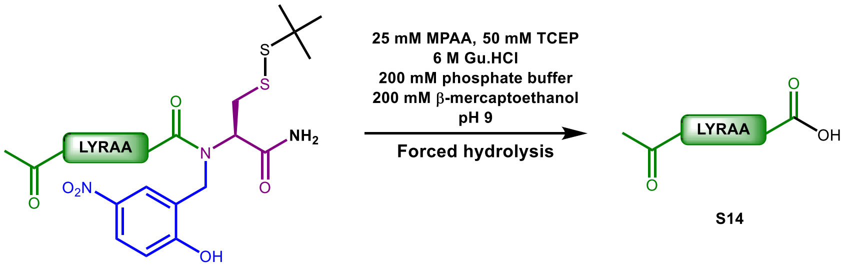

Supplementary scheme S18: Hydrolysis of peptide **7** to give HPLC standard **S14**.[4]

Reaction conditions inspired by Kent *et al.*[4]

Peptide **7** (1 mM) was incubated under argon in 100 µL of a deoxygenated 200 mM pH 9 sodium phosphate buffer containing 25 mM MPAA, 50 mM TCEP, 200 mM β-mercaptoethanol and 6 M guanidine hydrochloride. The reaction was monitored by RP-HPLC (Chromolith, gradient: 20-70 % B/A over 6 min). After 16h at room temperature, the starting material was consumed and the product **S14** was characterized and purified to serve as a HPLC standard.

S14:

MALDI-TOF (*m/z*): $[MH]^+$ calcd for $C_{29}H_{47}N_8O_8$: 635.35, found: 635.28.

HPLC: retention time: 1.04 min (Chromolith, gradient: 20-70 % B/A over 6 min).

[4] Gates Z. P., Stephan J. R., Lee D. J., Kent S. B. H., *Chem. Comm.,* 2013, **49**, 786-788

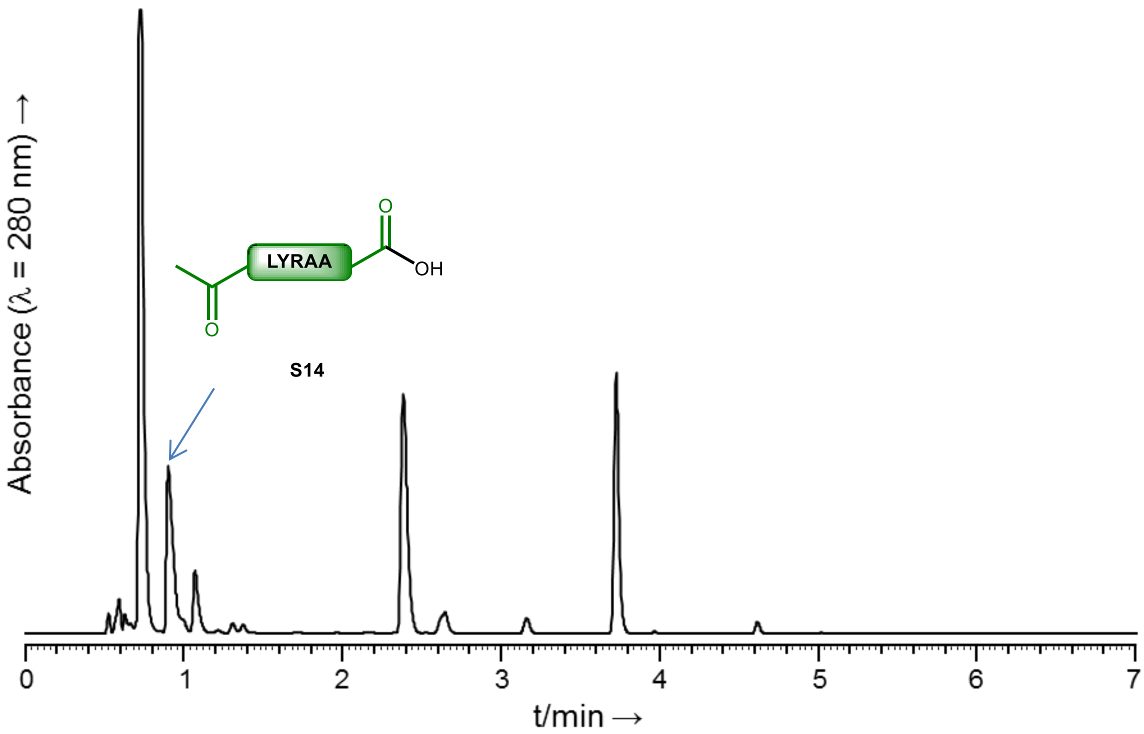

Supplementary figure S58: HPLC trace of crude **S14**.

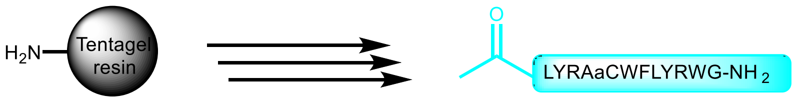

Supplementary scheme S19: Synthesis of HPLC standard **15**.

Peptide **15** was obtained through automated SPPS (protocol p S3) starting from Tentagel resin (476 mg, 0.21 mmol/g, 0.1 mmol). The peptide-resin was cleaved (protocol p S3) in order to characterize the corresponding peptide **15**.

15:

MALDI-TOF (*m/z*): $[MH]^+$ calcd for $C_{86}H_{117}N_{22}O_{16}S$: 1745.87, found: 1745.84.
HPLC: retention time: 3.94 min (Chromolith, gradient: 20-70 % B/A over 6 min).

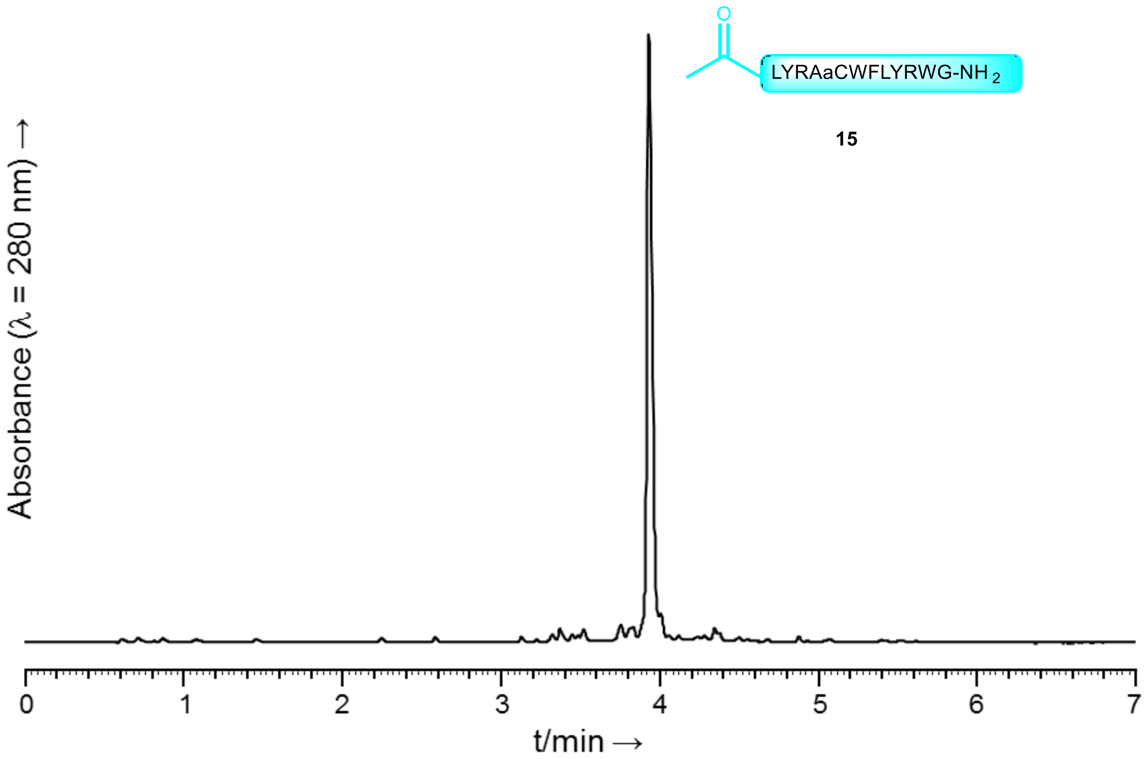

Supplementary figure S59: HPLC trace of crude **15**.

An HPLC co-injection of the NCL mixture after 24h using **9** with HPLC standards **S14** and **15** allowed determining the amount of hydrolysis of the thioester moiety and epimerization at the ligation site after a day of reaction.

**15** formed (%)	**< 0.4**
**S14** formed (%)	**< 4**

Supplementary table S4: Hydrolysis and epimerization during a typical NCL with **9**.

9d- Influence of the buffer

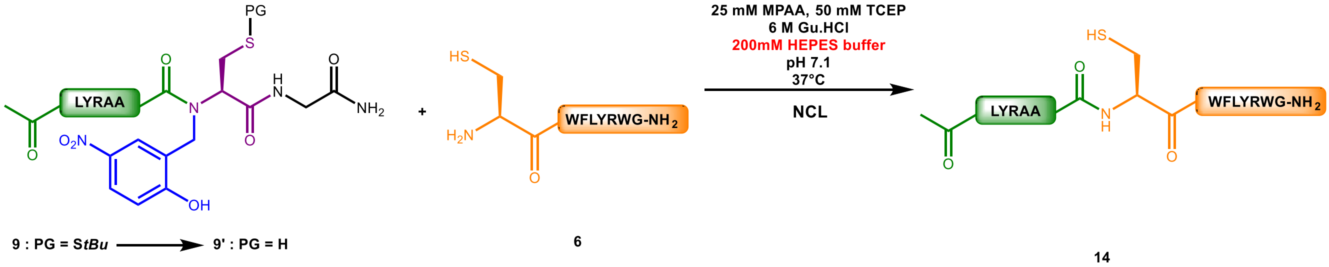

Supplementary scheme S20: NCL in HEPES buffer with **9**.

500 µL of a degassed 0.2 M pH 7.1 HEPES buffer containing 25 mM MPAA, 50 mM TCEP and 6 M guanidine hydrochloride were added to 0.8 mg peptide **9** (final concentration 1.5 mM) and 0.7 mg peptide **6** (final concentration 1 mM) under argon.

The ligation was carried out at 37°C and monitored by RP-HPLC (Chromolith, gradient: 20-70 % B/A over 6 min). For this, aliquots of 2 µL were diluted in 108 µL of 1.5 % TFA in water and 100 µL were injected in HPLC.

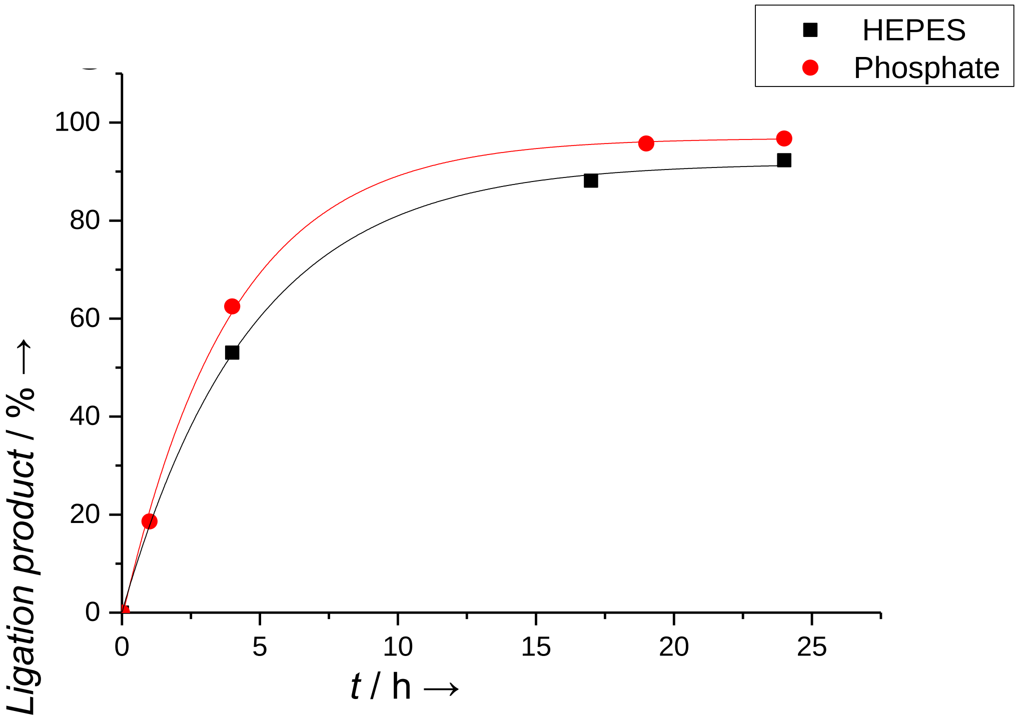

Supplementary figure S60: Comparison of reaction kinetics using phosphate or HEPES buffer using **9**.

9e- Influence of masking phenol or thiol groups

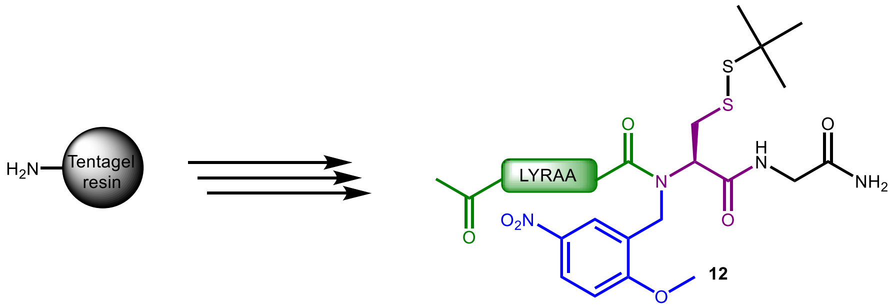

Supplementary scheme S21: Synthesis of *O*-methylated peptide **12**.

Tentagel R resin (476 mg, 0.21 mmol/g, 0.1 mmol) was introduced into a polypropylene syringe fitted with a polypropylene frit and a PTFE stopcock and swollen with NMP. Fmoc group was removed by three successive treatments with 20% piperidine in NMP (10 mL) for 3 min. Then, Fmoc-Rink-OH (270 mg, 0.5 mmol, 5 equiv.) and HATU (190 mg, 0.5 mmol, 5 equiv.) were dissolved in NMP (2 mL) prior to addition of *i*Pr_2NEt (175 µL, 1 mmol, 10 equiv.). The resulting solution was immediately added to the resin. The syringe was left for 1.5 h under gentle stirring,

then the syringe was drained and the resin was washed with NMP. Fmoc group was removed by three successive treatments with 20% piperidine in NMP (10 mL) for 3 min. Fmoc-Gly-OH (297 mg, 1 mmol, 10 equiv.) and HCTU (372 mg, 0.90 mmol, 9 equiv.) were dissolved in NMP (2 mL) prior to addition of *i*Pr_2NEt (348 µL, 2 mmol, 20 equiv.). The resulting solution was immediately added to the resin. The syringe was left for 1 h under gentle stirring, then the syringe was drained and the resin was washed with NMP. Then, Fmoc group was removed by three successive treatments with 20% piperidine in NMP (10 mL) for 3 min. Fmoc-Cys(S*t*Bu)-OH (432 mg, 1 mmol, 10 equiv.) and HCTU (372 mg, 0.90 mmol, 9 equiv.) were dissolved in NMP (10 mL) prior to addition of *i*Pr_2NEt (348 µL, 2 mmol, 20 equiv.). The resulting solution was immediately added to the resin. The syringe was left for 1.5 h under gentle stirring, then the syringe was drained and the resin was washed with NMP. Fmoc group was removed by three successive treatments with 20% piperidine in NMP (10 mL) for 3 min. The 2-hydroxy-5-nitrobenzyl linker was then introduced following the general procedure (p S5). The Ac-LYRAA sequence was installed by standard SPPS procedure (protocol p S3), an extended double coupling (2 × 2h) was performed for the first alanine and a normal double coupling (2 × 30 min) was performed for the arginine. Peptide resin was then treated with 20% piperidine in NMP (10 mL, 3 × 3 min), washed with NMP then CH_2Cl_2. Methylation of the phenol group was performed using a solution of MeI (50 equiv.), *i*Pr_2NEt (50 equiv.) in DMF for 4 h and the resin was washed with NMP and CH_2Cl_2. Finally, peptide-resin was cleaved following the general procedure (p S3) to give compound **12**, which was purified by semi-preparative HPLC (Nucleosil, gradient: 40-45 % B/A over 10 min).

12:

MALDI-TOF (*m/z*): $[MH]^+$ calcd for $C_{46}H_{71}N_{12}O_{12}S_2$: 1047.48, found: 1047.46.
HPLC: retention time: 8.94 min (Chromolith, gradient: 5-50 % B/A over 10 min).

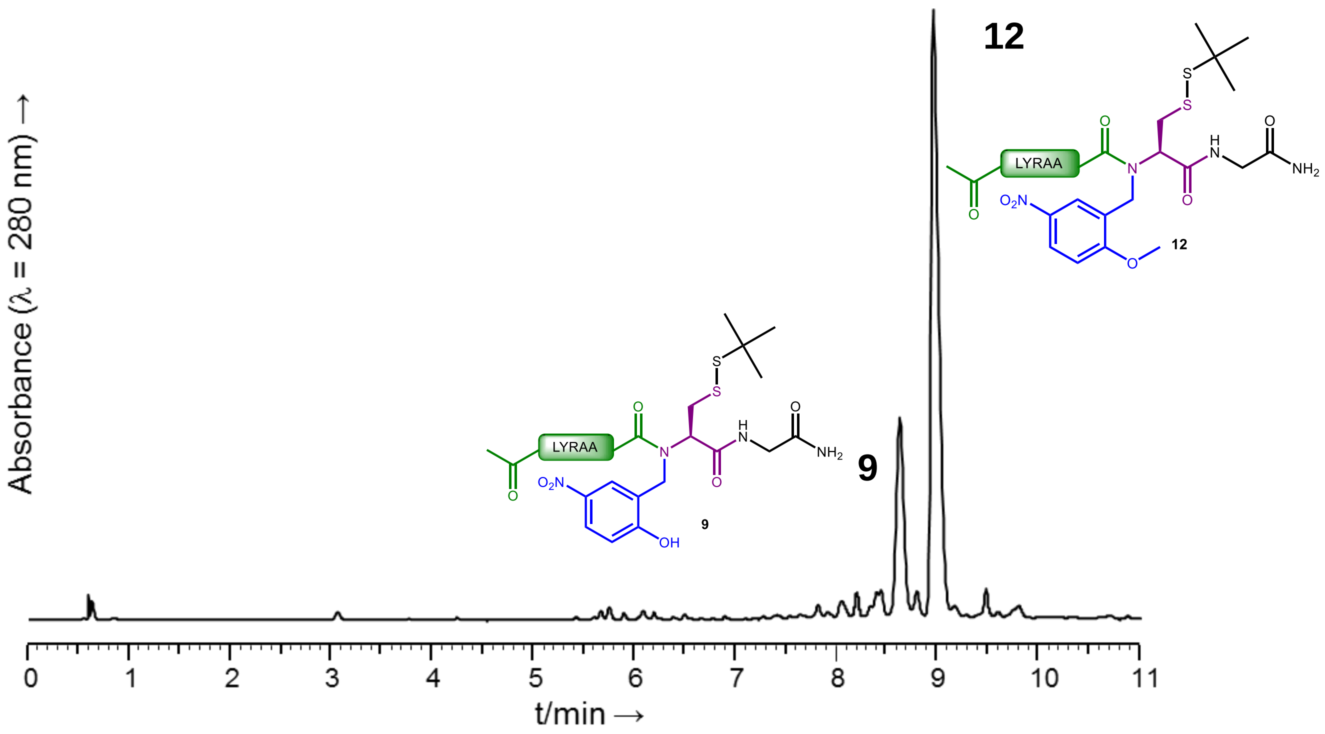

<u>Supplementary figure S61</u>: HPLC trace of crude **12**.

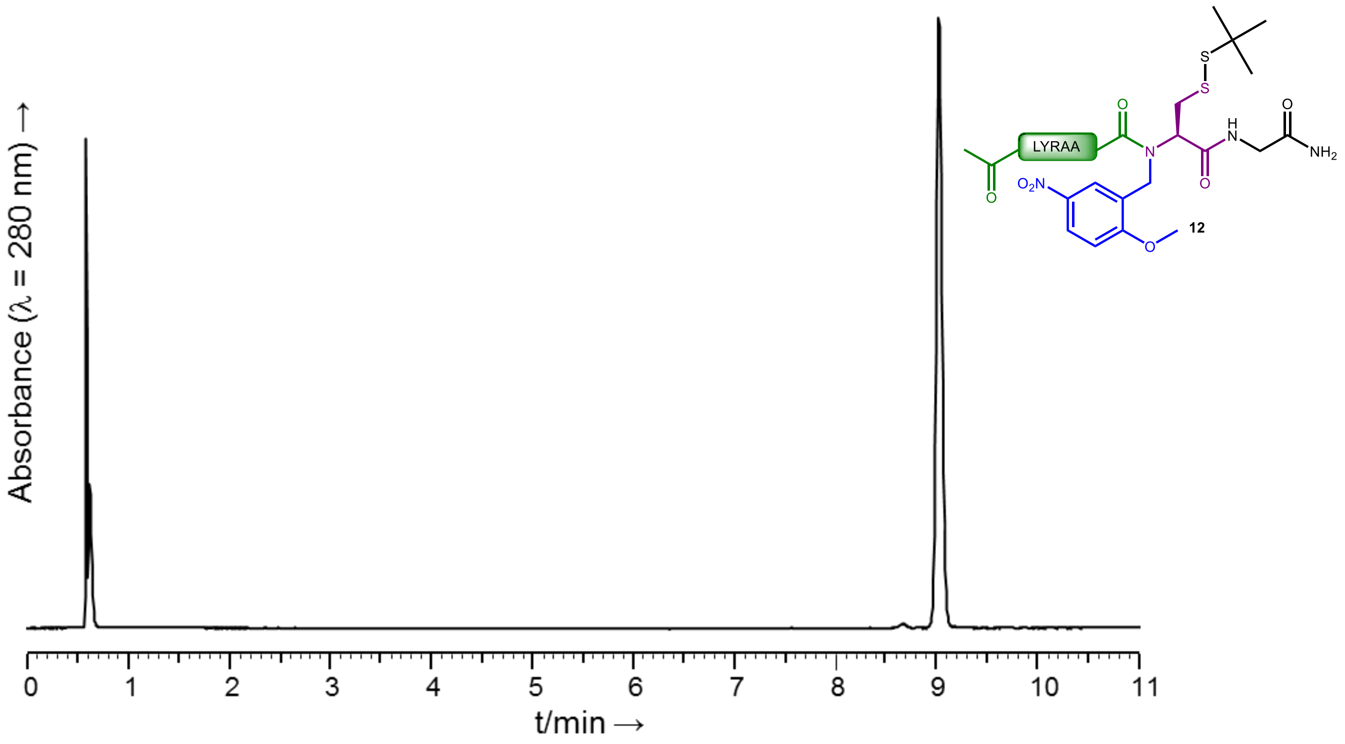

<u>Supplementary figure S62</u>: HPLC trace of pure **12**.

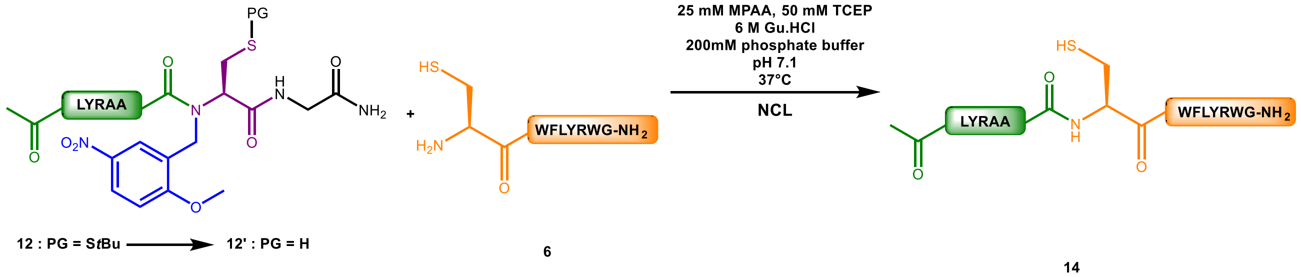

Supplementary scheme S22: Native chemical ligation with *O*-methylated **12**.

Ligation with **12**:

500 µL of a degassed 0.2 M pH 7.1 sodium phosphate buffer containing 25 mM MPAA, 50 mM TCEP and 6 M guanidine hydrochloride were added to 1.05 mg of the peptide **12** (final concentration 2 mM) and 0.69 mg peptide **6** (final concentration 1 mM) under argon. The ligation was carried out at 37°C and monitored by RP-HPLC (Chromolith, gradient: 20-70 % B/A over 6 min). For this, aliquots of 2 µL were diluted in 108 µL of 1.5 % TFA in water and 100 µL were injected in HPLC.

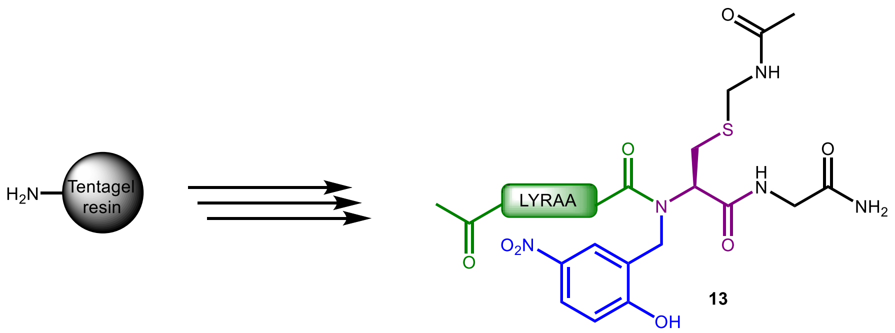

Supplementary scheme S23: Synthesis of the *S*-Acm peptide **13**.

Tentagel R resin (476 mg, 0.21 mmol/g, 0.1 mmol) was introduced into a polypropylene syringe fitted with a polypropylene frit and a PTFE stopcock and swollen with NMP. Fmoc group was removed by three successive treatments with 20% piperidine in NMP (10 mL) for 3 min. Then, Fmoc-Rink-OH (270 mg, 0.5 mmol, 5 equiv.) and HATU (190 mg, 0.5 mmol, 5 equiv.) were dissolved in NMP (2 mL) prior to addition of *i*Pr_2NEt (175 µL, 1 mmol, 10 equiv.). The resulting solution was immediately added to the resin. The syringe was left for 1.5 h under gentle stirring, then the syringe was drained and the resin was washed with NMP. Fmoc group was removed by three successive treatments with 20% piperidine in NMP (10 mL) for 3 min. Fmoc-Gly-OH (297 mg, 1 mmol, 10 equiv.) and HCTU (372 mg, 0.90 mmol, 9 equiv.) were dissolved in NMP (2 mL) prior to addition of *i*Pr_2NEt (348 µL, 2 mmol, 20 equiv.). The resulting solution was immediately added to the resin. The syringe was left for 1 h under gentle stirring, then the syringe was drained and the resin was washed with NMP. Then, Fmoc group was removed by three successive treatments with 20% piperidine in NMP (10 mL) for 3 min. Fmoc-Cys(Acm)-OH (414.5 mg, 1 mmol, 10 equiv.) and HCTU (372 mg, 0.90 mmol, 9 equiv.) were dissolved in NMP (10 mL) prior to addition of *i*Pr_2NEt (348 µL, 2 mmol, 20 equiv.). The resulting

solution was immediately added to the resin. The syringe was left for 1.5 h under gentle stirring, then the syringe was drained and the resin was washed with NMP. Fmoc group was removed by three successive treatments with 20% piperidine in NMP (10 mL) for 3 min. The 2-hydroxy-5-nitrobenzyl linker was then introduced following the general procedure (p S5). The Ac-LYRAA sequence was installed by standard SPPS procedure (protocol p S3), an extended double coupling (2 × 2h) was performed for the first alanine and a normal double coupling (2 × 30 min) was performed for the arginine. Peptide resin was then treated with 20% piperidine in NMP (10 mL, 3 × 3 min), washed with NMP then CH_2Cl_2. Finally, the peptide was cleaved following the general procedure p S3 to give compound **13**. **13** has been purified by semi-preparative HPLC (Nucleosil, gradient: 20-35 % B/A over 5 min).

13:

MALDI-TOF (*m/z*): $[MH]^+$ calcd for $C_{44}H_{66}N_{13}O_{13}S$: 1016.46, found: 1016.46.
HPLC: retention time: 5.88 min (Chromolith, gradient: 5-50 % B/A over 10 min).

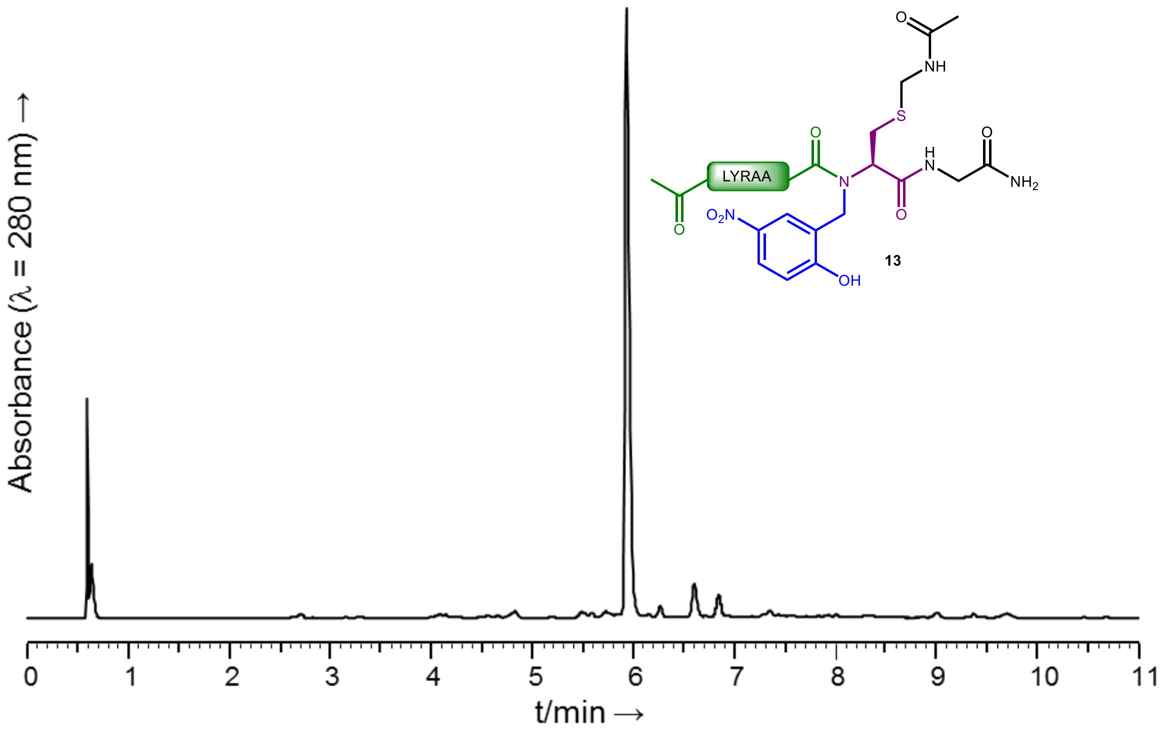

Supplementary figure S63: HPLC trace of crude **13**.

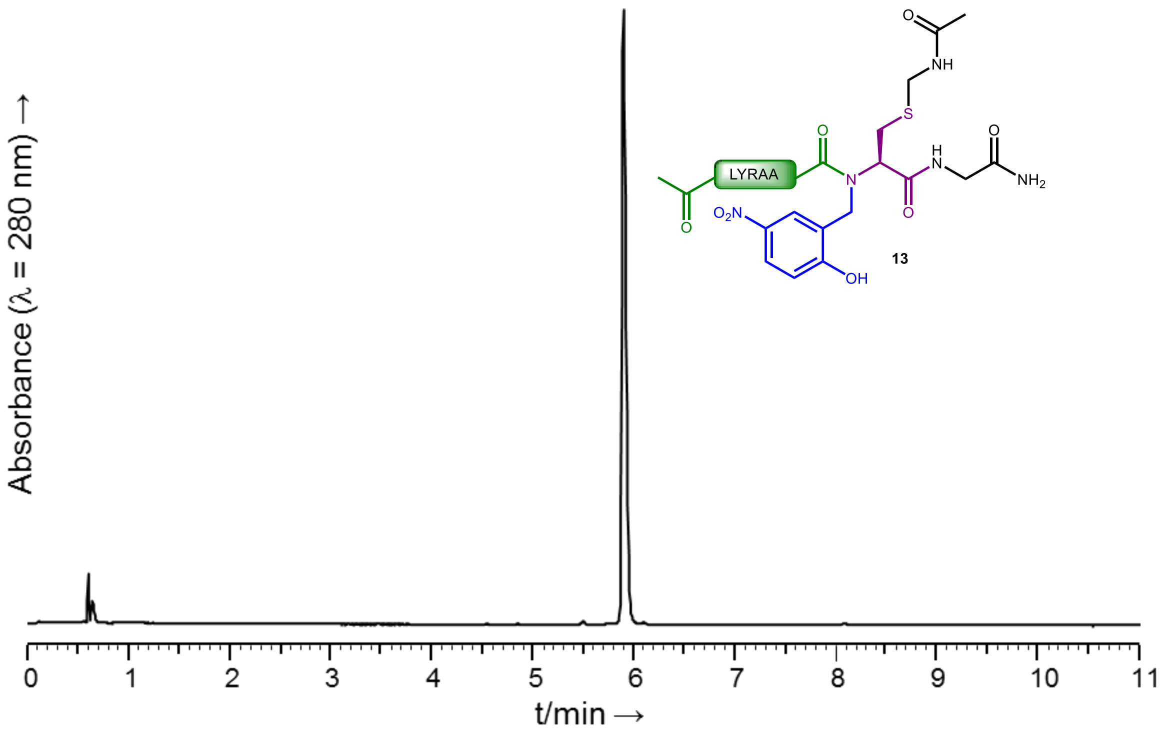

Supplementary figure S64: HPLC trace of pure **13**.

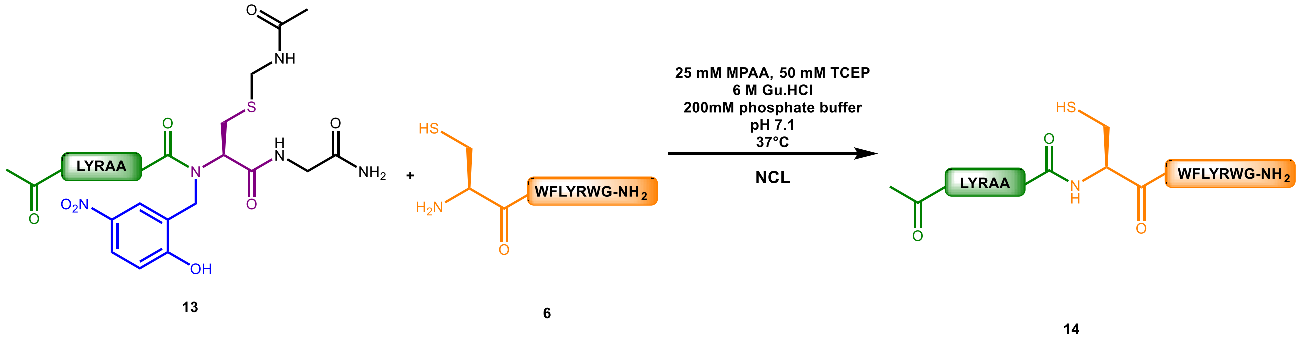

Supplementary scheme S24: Native chemical ligation with *S*-Acm peptide **13**.

Ligation with **13**:

500 µL of a degassed 0.2 M pH 7.1 sodium phosphate buffer containing 25 mM MPAA, 50 mM TCEP and 6 M guanidine hydrochloride were added to 1.2 mg of the peptide **13** (final concentration 2 mM) and 0.69 mg peptide **6** (final concentration 1 mM) under argon. The ligation was carried out at 37°C and monitored by RP-HPLC (Chromolith, gradient: 20-70 % B/A over 6 min). For this, aliquots of 2 µL were diluted in 108 µL of 1.5 % TFA in water and 100 µL were injected in HPLC.

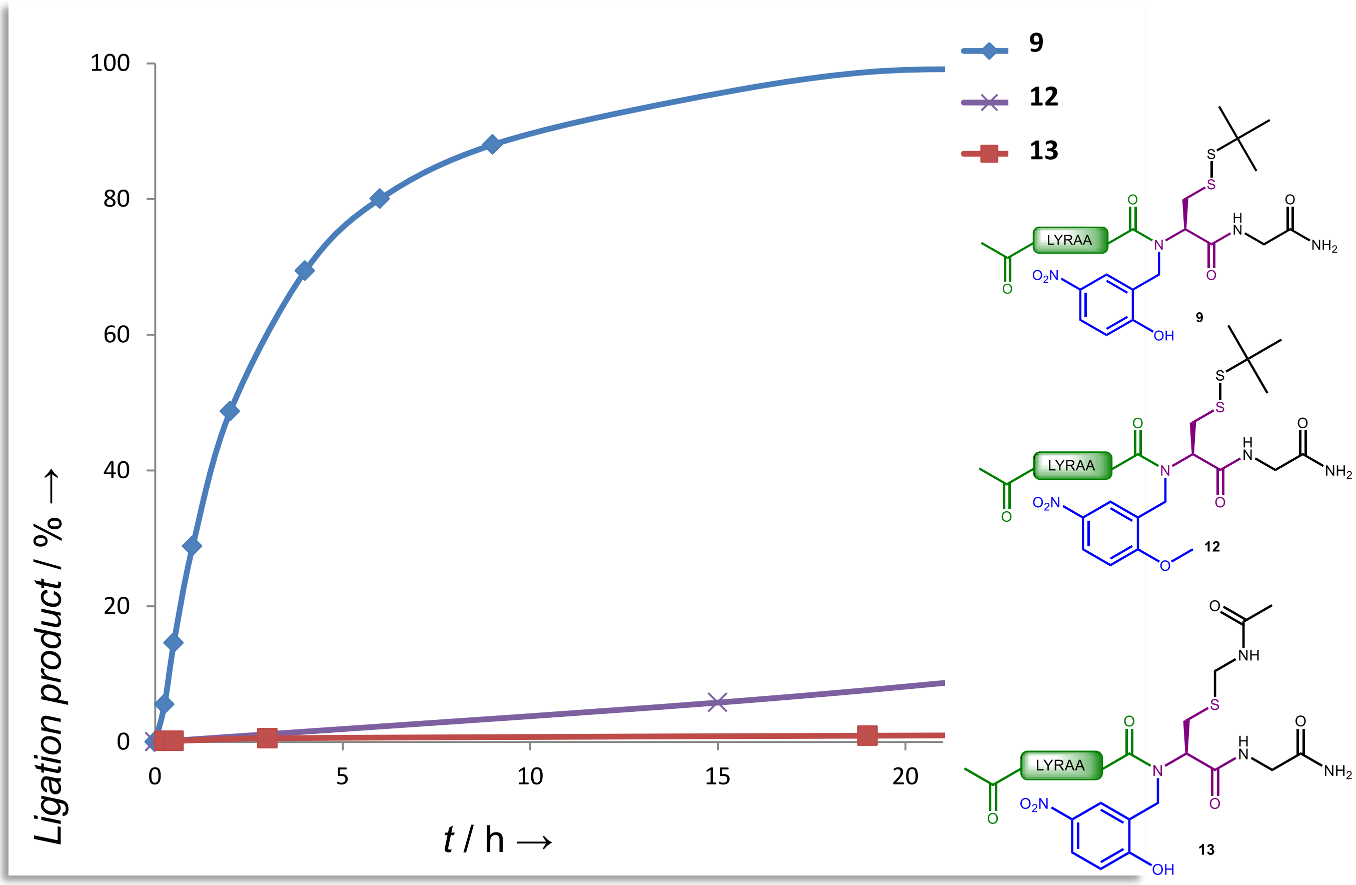

Supplementary figure S65: Ligation kinetics observed using **12** or **13** compared to ligation using **9**.

9f- Influence of the pH

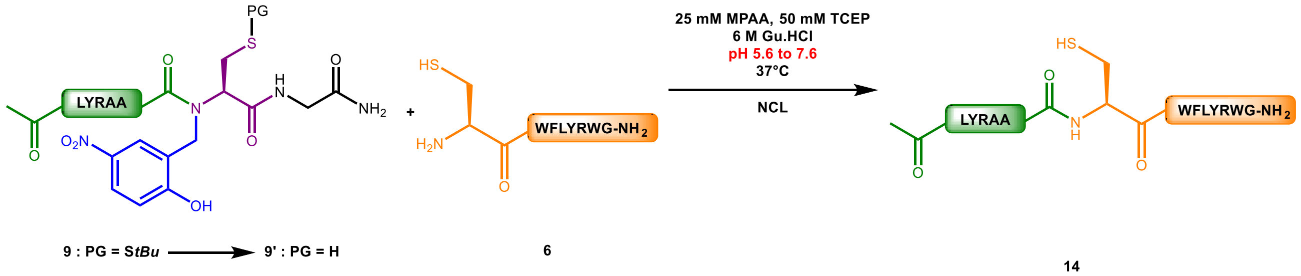

Supplementary scheme S25: NCL at different pHs with **9**.

500 µL of different degassed 0.2 M sodium phosphate buffers (pH 5.6, 6.1, 6.6, 7.1, 7.6) containing 25 mM MPAA, 50 mM TCEP and 6 M guanidine hydrochloride were added to 0.8 mg of the peptide **9** (final concentration 1.5 mM) and 0.69 mg peptide **6** (final concentration 1 mM) under argon. The ligation was carried out at 37°C and monitored by RP-HPLC (Chromolith, gradient: 20-70 % B/A over 6 min). For this, aliquots of 2 µL were diluted in 108 µL of 1.5 % TFA in water and 100 µL were injected in HPLC.

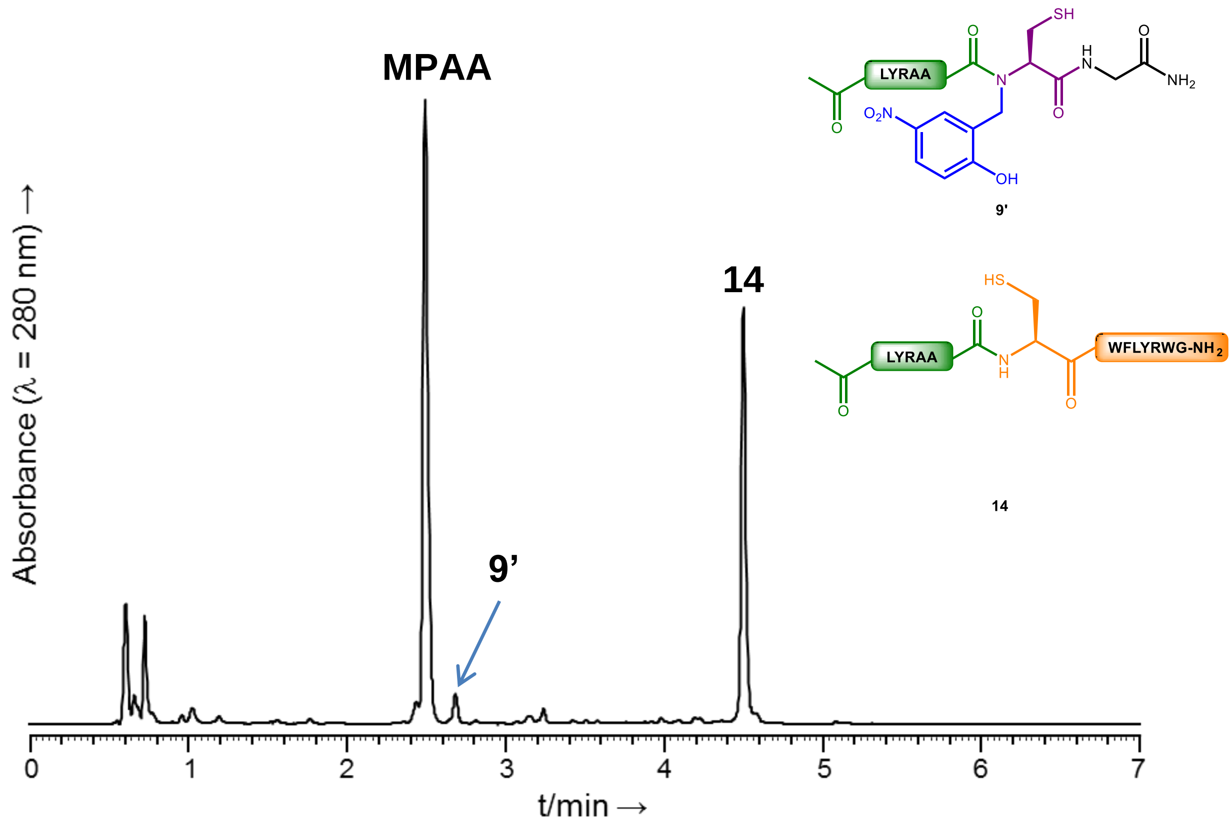

Supplementary figure S66: NCL after 24h using **9** at pH 6.6.

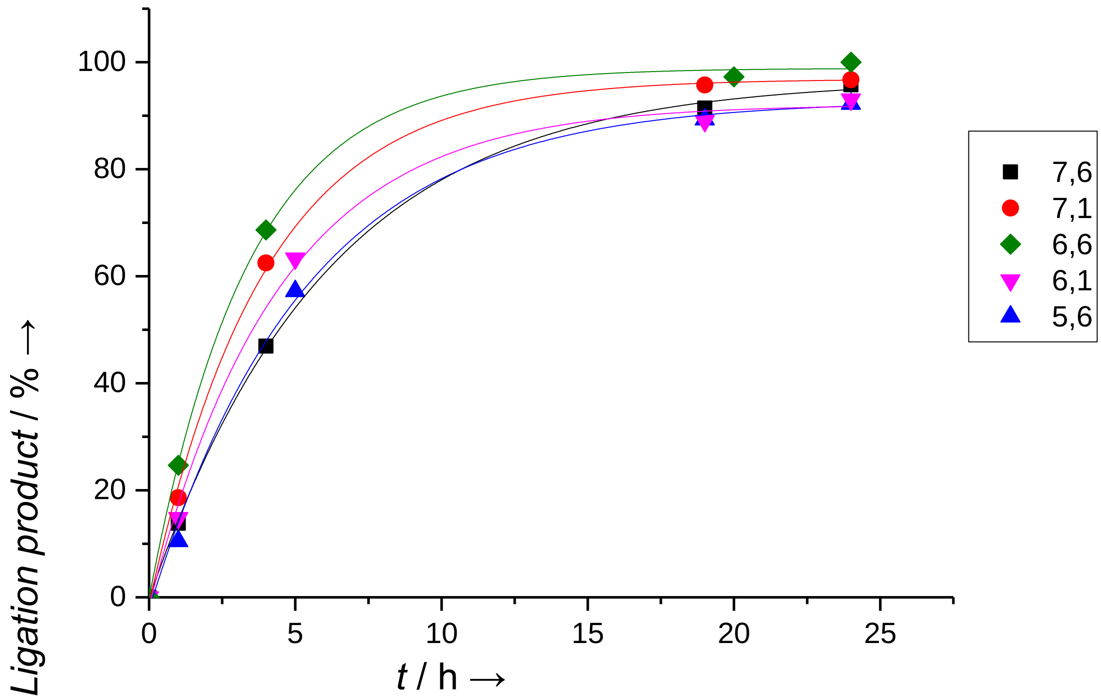

Supplementary figure S67: Ligation kinetics observed at different pHs using **9**.

10-Influence of the C-terminal aa: NCL with model Ac-LYRAX(Hnb)C peptides

10a- Synthesis of peptide crypto-thioester Ac-LYRAS-(Hnb)C(S*t*Bu)G-NH_2 (**10**)

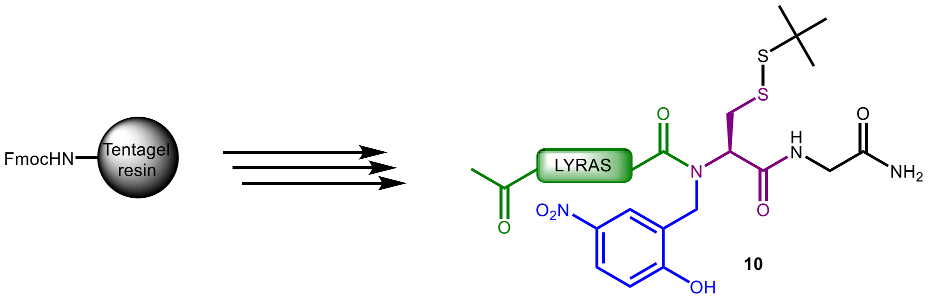

10

Supplementary scheme S26: Synthesis of model peptide **10**.

Peptide **10** was obtained through automated SPPS (protocol p S3) and using the automated reductive amination (protocol p S34) starting from Fmoc-Rink tentagel resin (120 mg, 0.21 mmol/g, 25 µmol). Fmoc-Ser(O*t*Bu)-OH was coupled for 3 × 30 min on the (Hnb)C(S*t*Bu)G-Rink Tentagel resin. The complete sequence was installed through standard Fmoc-SPPS (protocol p S3), Fmoc-Arg(Pbf)-OHwas coupled twice (2 × 30 min) and a final piperidine treatment (20 % in NMP, 3 mL, 3 min, ×3) was performed. Peptide-resin was cleaved (protocol p S3) and peptide **10** was purified by semi-preparative RP-HPLC (Nucleosil, gradient: 40-55 % B/A over 12 min).

10:

ESI-HRMS (*m/z*): $[MH]^+$ calcd for $C_{45}H_{69}N_{12}O_{13}S_2$: 1049.4548, found: 1049.4532.
HPLC: retention time: 2.14 min (Chromolith, gradient: 30-60 % B/A over 5 min).

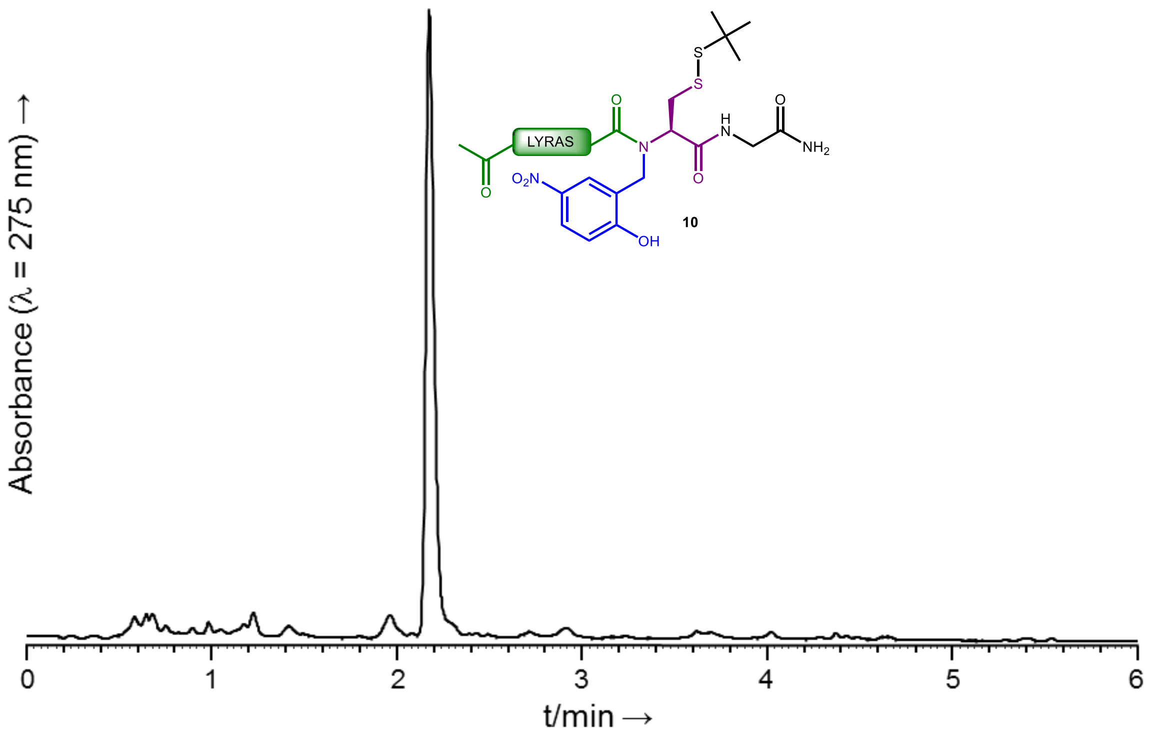

Supplementary figure S68: HPLC trace of crude **10**.

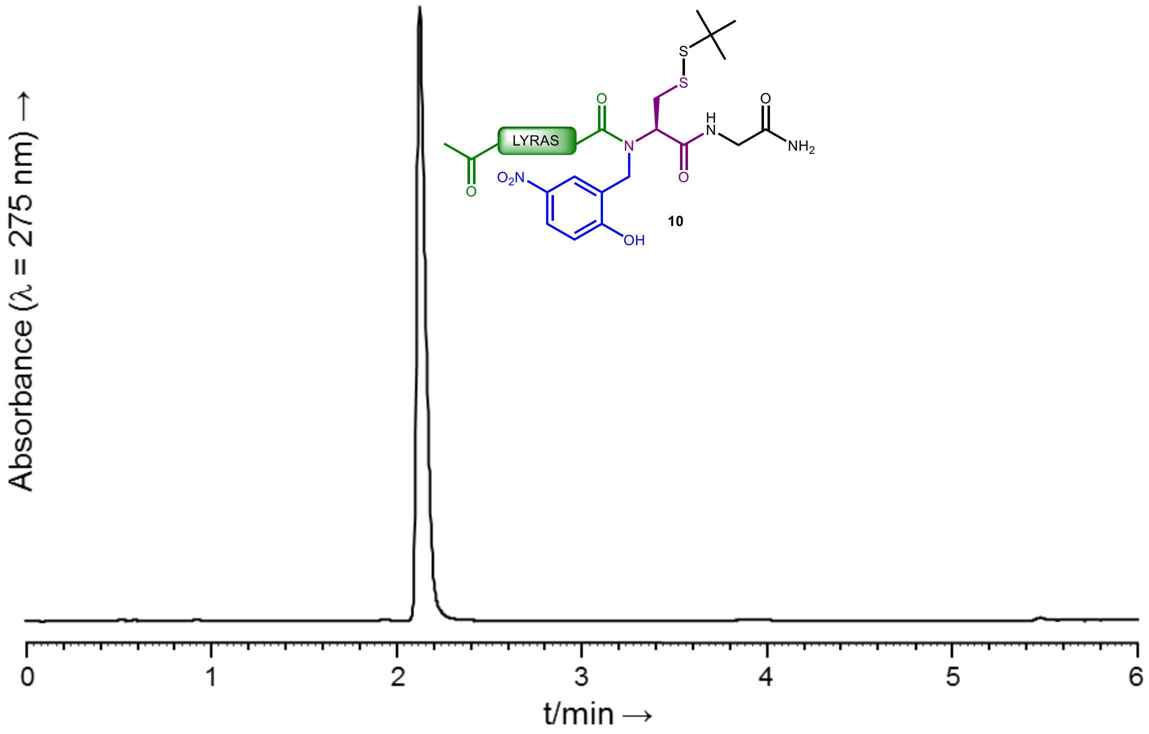

Supplementary figure S69: HPLC trace of pure **10**.

10b- NCL with peptide crypto-thioester Ac-LYRAS-(Hnb)C(S*t*Bu)G-NH_2 (**10**)

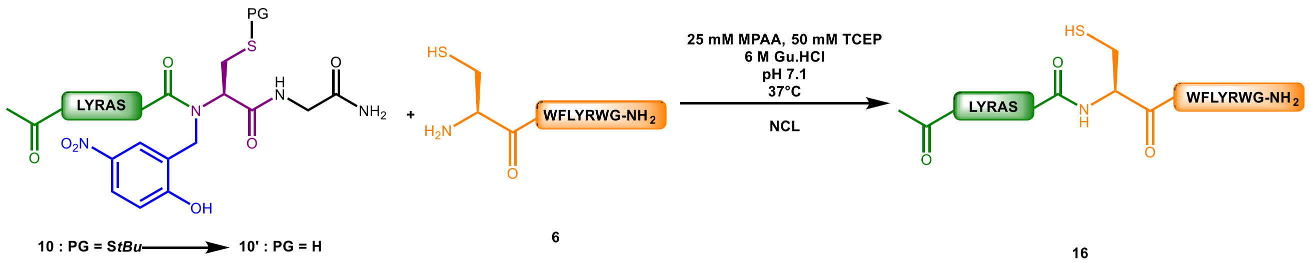

Supplementary scheme S27: Native chemical ligation with **10**.

250 µL of a degassed 0.2 M pH 7.1 sodium phosphate buffer containing 25 mM MPAA, 50 mM TCEP and 6 M guanidine hydrochloride were added to 0.53 mg of the peptide **10** (final concentration 2 mM) and 0.30 mg peptide **6** (final concentration 1 mM) under argon. The ligation was carried out at 37°C and monitored by RP-HPLC (Chromolith, gradient: 20-70 % B/A over 6 min). For this, aliquots of 2 µL were diluted in 108 µL of 1.5 % TFA in water and 100 µL were injected in HPLC.

Ligation product 16:

ESI-HRMS (*m/z*): $[MH]^+$ calcd for $C_{86}H_{117}N_{22}O_{17}S$: 1761.8688, found: 1761.8687.
HPLC: retention time: 3.81 min (Chromolith, gradient: 20-70 % B/A over 6 min).

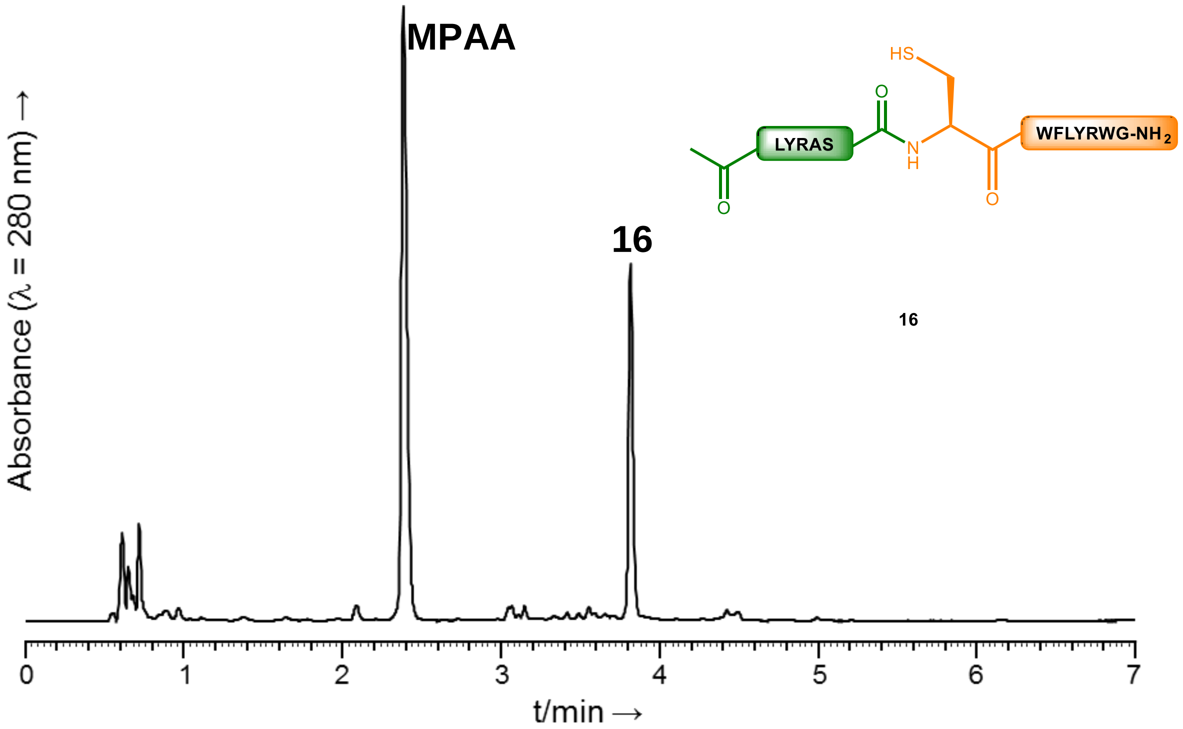

Supplementary figure S70: NCL after 25h using **10**.

10c-Synthesis of peptide crypto-thioester Ac-LYRAV-(Hnb)C(S*t*Bu)G-NH_2 (**11**)

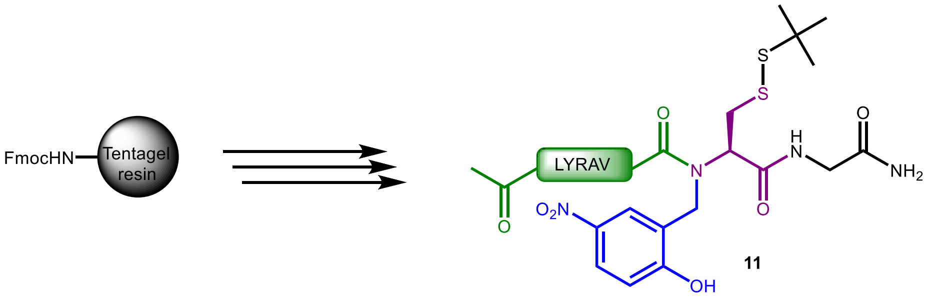

Supplementary scheme S28: Synthesis of model peptide **11**.

Peptide **11** was obtained through automated SPPS (protocol p S3) and using the automated reductive amination (protocol p S34) starting from Fmoc-Rink tentagel resin (120 mg, 0.21 mmol/g, 25 µmol). Fmoc-Val-OH has been coupled for 5 × 30 min on the (Hnb)C(S*t*Bu)G-Rink Tentagel resin. The complete sequence was installed through standard Fmoc-SPPS (protocol p S3), Fmoc-Arg(Pbf)-OH was coupled for 2 × 30 min and a final piperidine (20 % in NMP, 3mL, 3 min, ×3) was performed. Peptide-resin was cleaved (protocol p S3) and peptide **11** was purified by semi-preparative RP-HPLC (Nucleosil, gradient: 40-55 % B/A over 12 min).

11:

ESI-HRMS (*m/z*): $[MH]^+$ calcd for $C_{47}H_{73}N_{12}O_{12}S_2$: 1061.4912, found: 1061.4891.
HPLC: retention time: 3.51 min (Chromolith, gradient: 30-60 % B/A over 5 min).

*****: minor compound observed (Val deletion) resulting from incomplete coupling of Fmoc-Val-OH because no acetylation-mediated capping step was performed.

***:**

ESI-HRMS (*m/z*): $[MH]^+$ calcd for $C_{42}H_{64}N_{11}O_{11}S_2$: 962.4228, found: 962.4224.
HPLC: retention time: 3.51 min (Chromolith, gradient: 30-60 % B/A over 5 min).

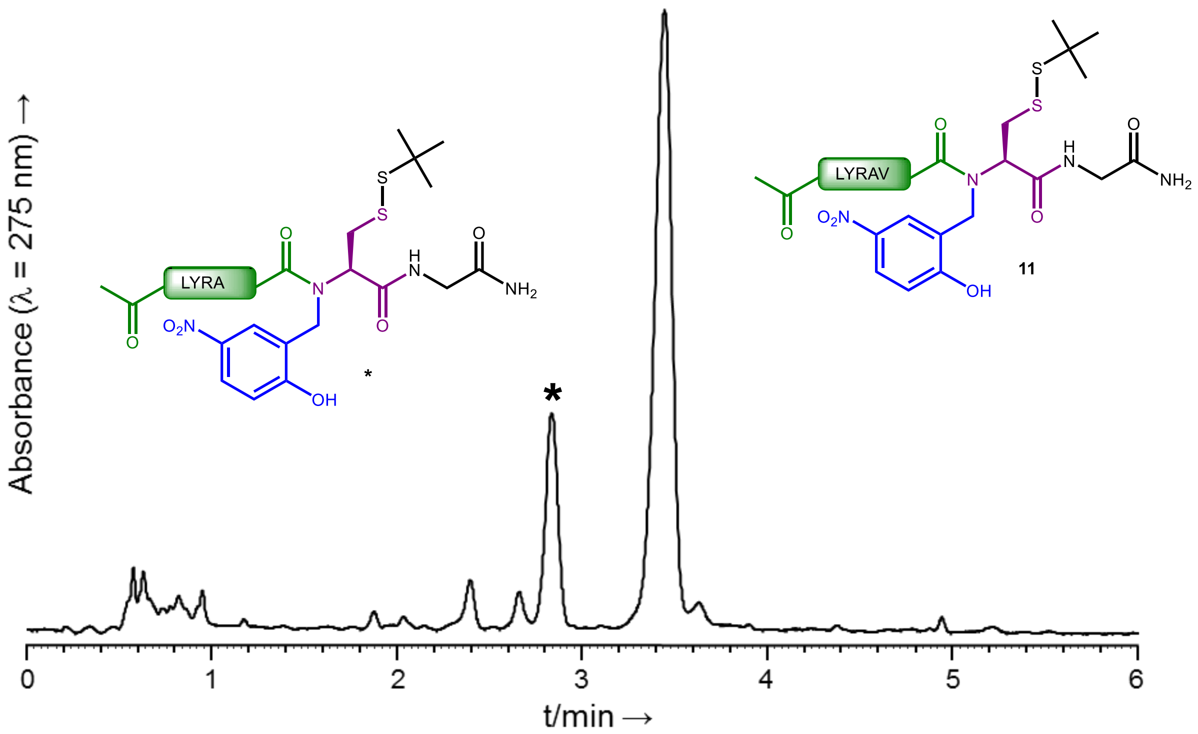

<u>Supplementary figure S71</u>: HPLC trace of crude **11**.

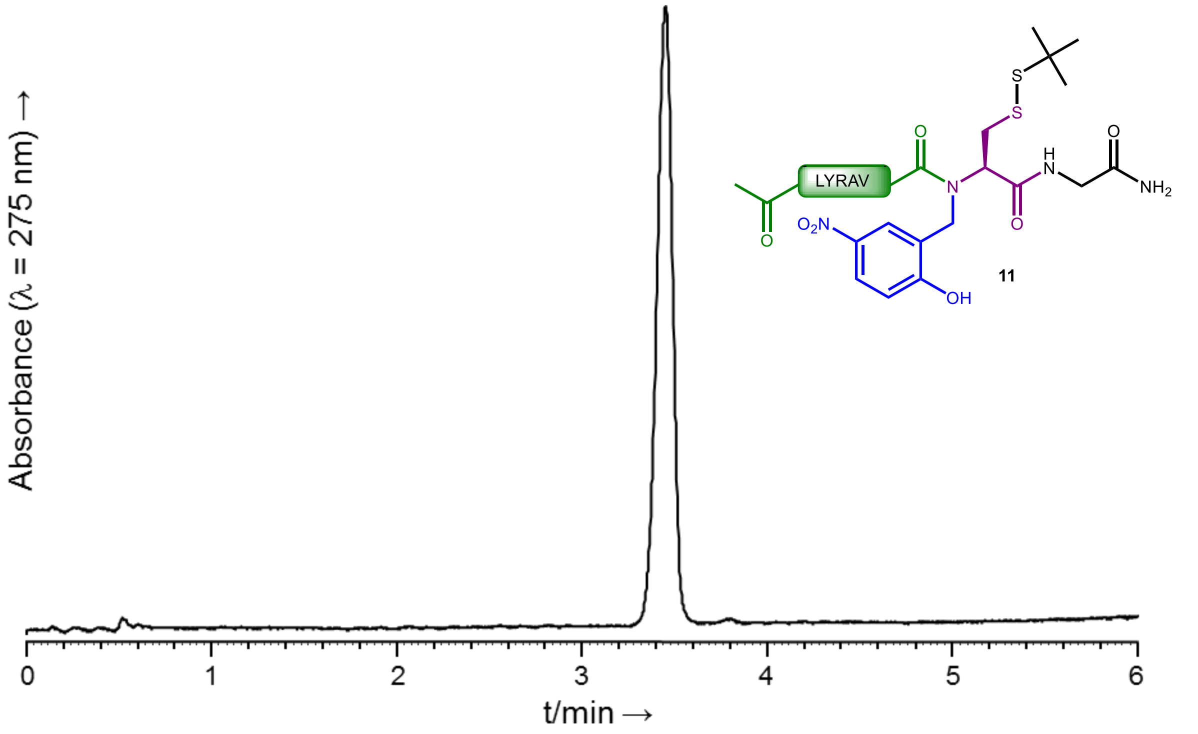

<u>Supplementary figure S72</u>: HPLC trace of pure **11**.

10d- NCL with peptide crypto-thioester Ac-LYRAV-(Hnb)C(S*t*Bu)G-NH_2 (**11**)

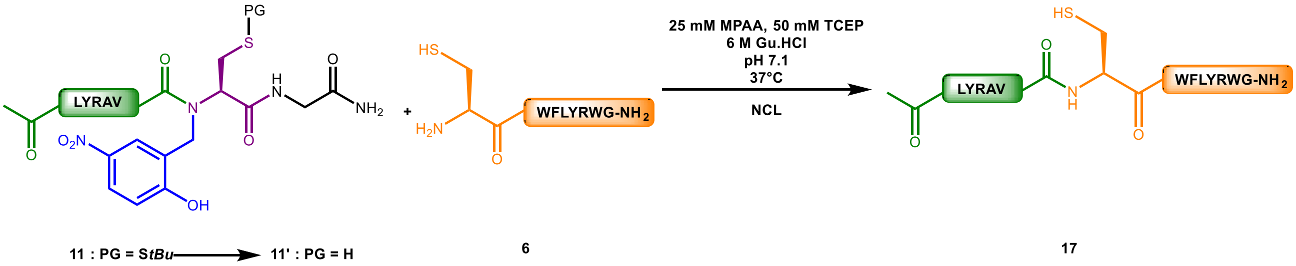

Supplementary scheme S29: Native chemical ligation with **11**.

250 µL of a degassed 0.2 M pH 7.1 sodium phosphate buffer containing 25 mM MPAA, 50 mM TCEP and 6 M guanidine hydrochloride were added to 0.53 mg of the peptide **11** (final concentration 2 mM) and 0.30 mg peptide **6** (final concentration 1 mM) under argon. The ligation was carried out at 37°C and monitored by RP-HPLC (Chromolith, gradient: 20-70 % B/A over 6 min). For this, aliquots of 2 µL were diluted in 108 µL of 1.5 % TFA in water and 100 µL were injected in HPLC.

10e- NCL with peptide crypto-thioester Ac-LYRAV-(Hnb)C(S*t*Bu)G-NH_2 (**11**) under optimized conditions

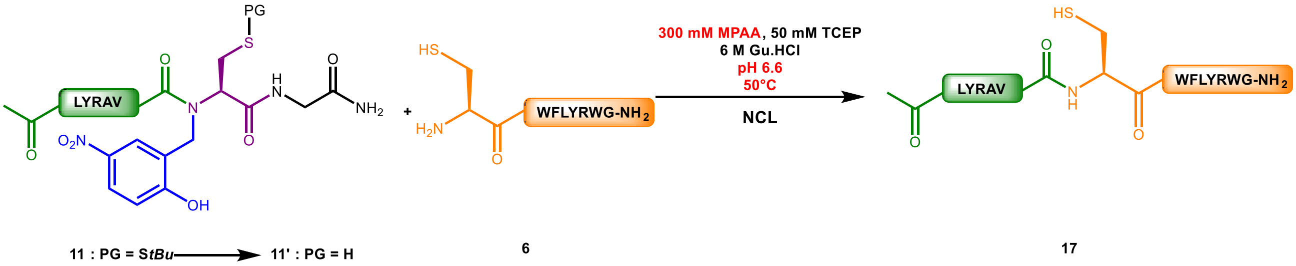

Supplementary scheme S30: Native chemical ligation with **11**.

500 µL of a degassed 0.2 M pH 6.6 sodium phosphate buffer containing 300 mM MPAA, 50 mM TCEP and 6 M guanidine hydrochloride were added to 1.2 mg of the peptide **11** (final concentration 2 mM) and 0.68 mg peptide **6** (final concentration 1 mM) under argon. The ligation was carried out at 50°C and monitored by RP-HPLC (Chromolith, gradient: 20-70 % B/A over 6 min). For this, aliquots of 2 µL were diluted in 108 µL of 1.5 % TFA in water, this aqueous phase was washed with di-ethyl ether (500 µL ×3) and 100 µL were injected in HPLC.

Ligation product 17:

ESI-HRMS (*m/z*): $[MH]^+$ calcd for $C_{88}H_{121}N_{22}O_{16}S$: 1773.9052, found: 1773.9059.
HPLC: retention time: 5.0 min (Chromolith, gradient: 20-70 % B/A over 6 min).

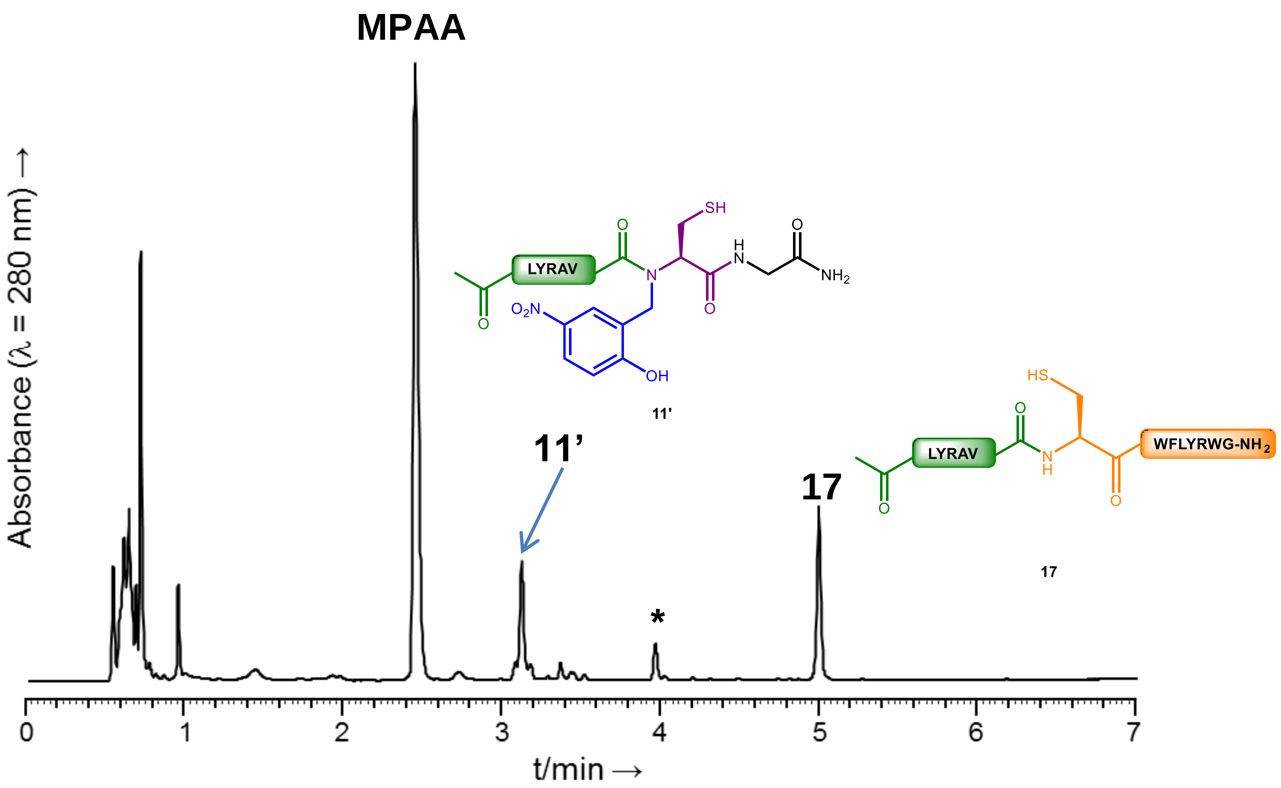

Supplementary figure S73: NCL after 24h using **11** under optimized conditions. *: non peptidic compound

10f- Determination of apparent second order kinetic constants

Kinetics of selected representative NCL reactions were modeled as an apparent second order reaction, considering the chemical equation:

$$\underset{A}{\text{H-CysPeptide1}} + \underset{B}{\text{Peptide2-(Hnb)Cys}} \xrightarrow{k_{app}} \underset{C}{\text{Peptide2CysPeptide1}}$$

following the general rate equation for $[A]_0 \neq [B]_0$:

$$kt = \frac{1}{[B]_0-[A]_0} \ln\left(\frac{[A]_0.([B]_0-[A])}{[B]_0.([A]_0-[A])}\right)$$

Values for t < 10min were not taken into account due to the early S*t*Bu deprotection step.

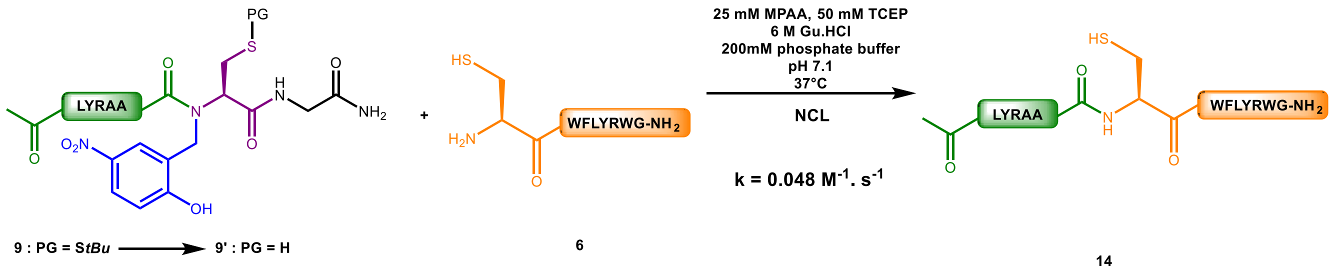

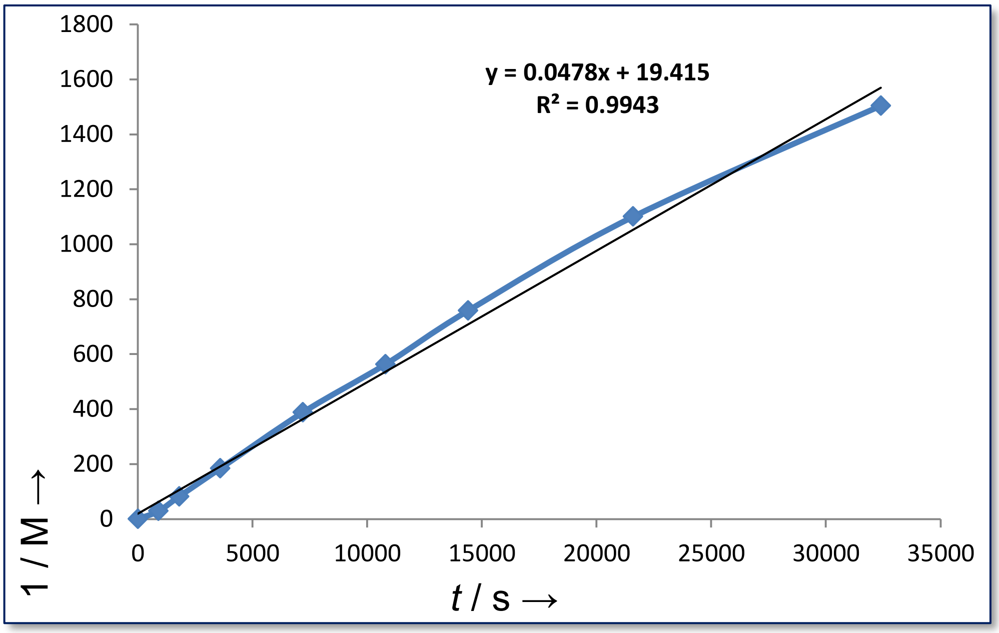

Supplementary figure S74: Apparent second order kinetic constant for NCL using **9**. (Conditions corresponding to Figure 3 of the article)

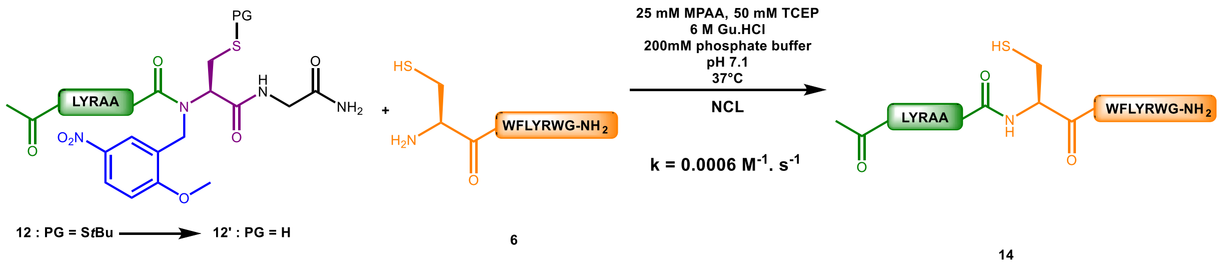

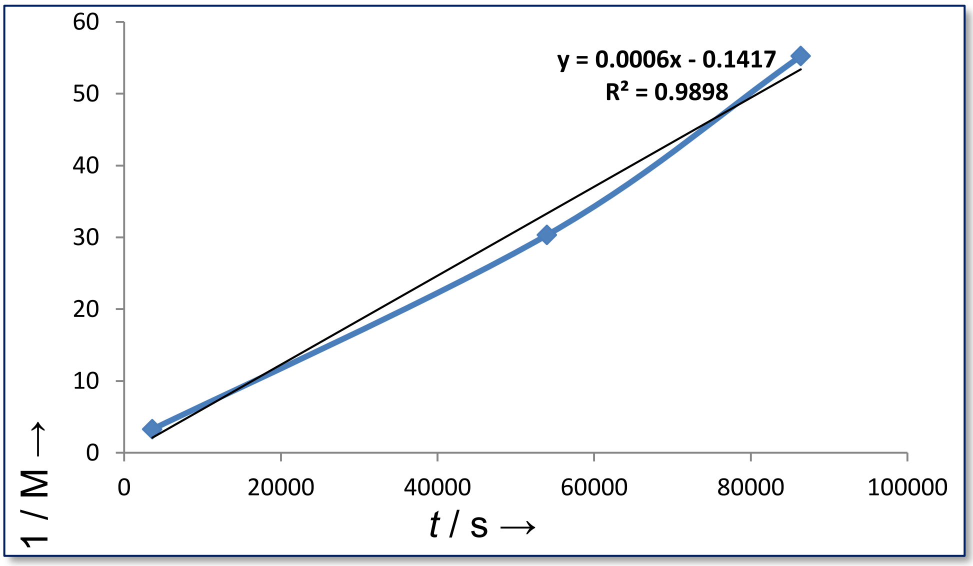

Supplementary figure S75: Apparent second order kinetic constant for NCL using **12**. (Conditions corresponding to Figure 3 of the article)

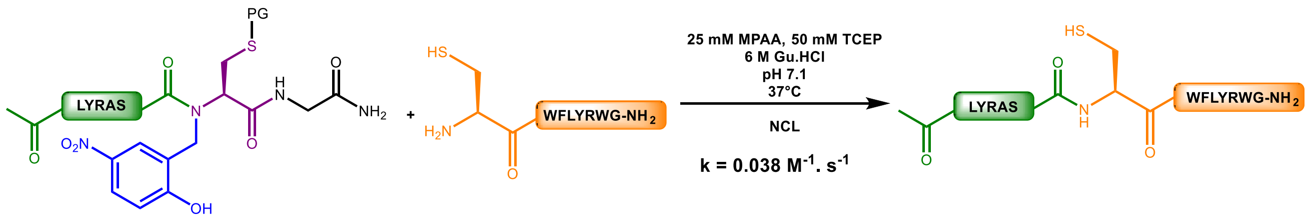

10 : PG = S*tBu* ⟶ 10' : PG = H 6 16

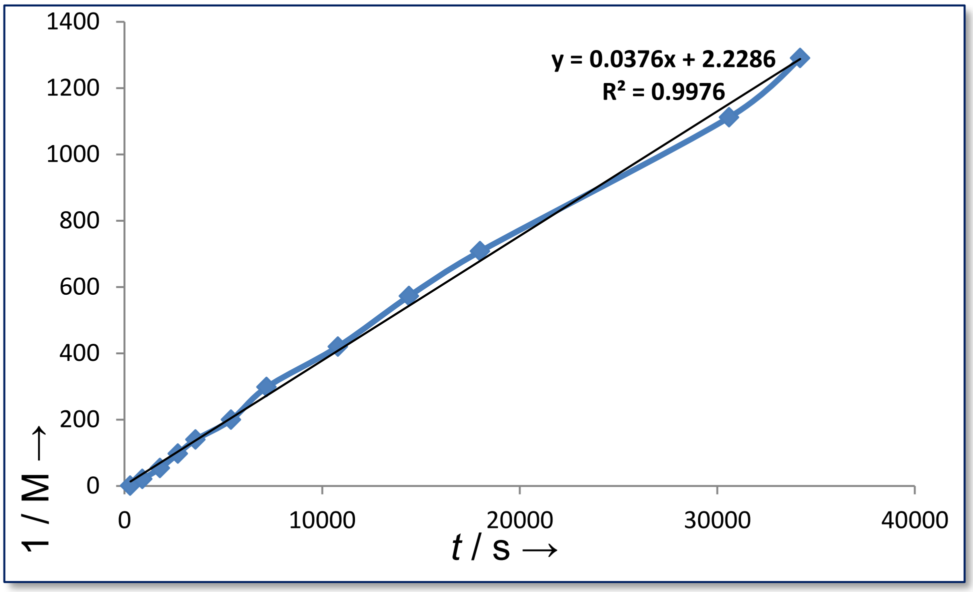

Supplementary figure S76: Apparent second order kinetic constant for NCL using **10**. (Conditions corresponding to Figure 3 of the article)

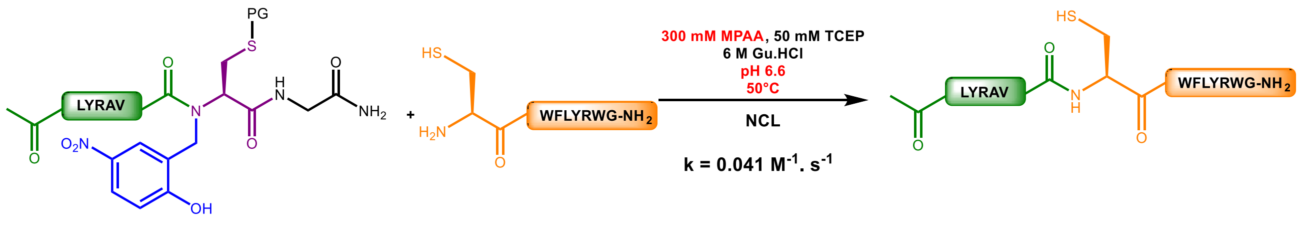

11 : PG = S*t*Bu ⟶ 11' : PG = H   6   17

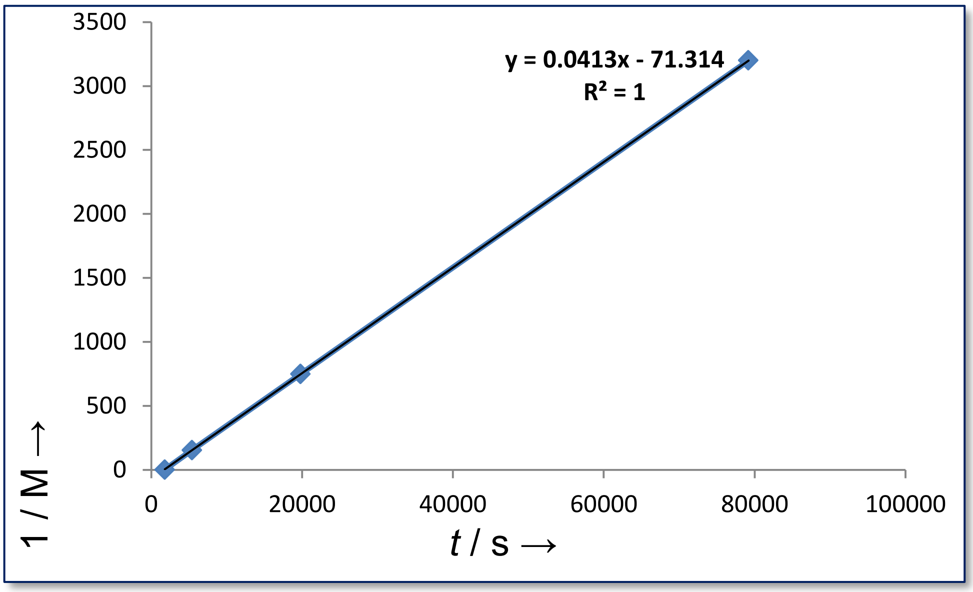

Supplementary figure S77: Apparent second order kinetic constant for NCL using **11** (Conditions corresponding to Figure 3 of the article)

11-Application to MT7

11a- Synthesis of cysteinyl peptide MT7 [42-65] (**19**)

Amino acid sequence of the MT7 [42-65] segment:

H-CAATCPKAEYRDVINCCGTDKCNK-OH

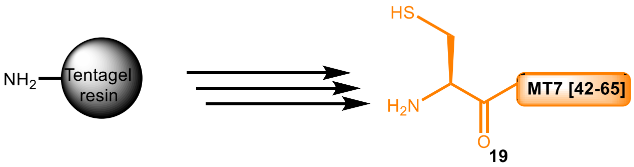

19

Supplementary scheme S31: Synthesis of cysteinyl peptide MT7 [42-65] **19**.

Peptide **19** was obtained through automated SPPS (protocol p S3) starting from Tentagel resin (476, 0.21 mmol/g, 0.1 mmol). Fmoc-K(Boc)-Mppa-OH (323.3 mg, 0.5 mmol, 5 equiv.) and HATU (190 mg, 0.5 mmol, 5 equiv.) were dissolved in NMP (2 mL) prior to addition of *i*Pr_2NEt (175 µL, 1 mmol, 10 equiv.). The resulting solution was immediately added to the resin. The syringe was left for 1.5 h under gentle stirring, then the syringe was drained and the resin was washed with NMP. Fmoc

group was removed by three successive treatments with 20% piperidine in NMP (10 mL) for 3 min and the complete sequence was installed trough standard Fmoc-SPPS (elongation yield = 71 %). After cleavage of a quarter of the resin (25 µmol, protocol p S3), product **19** was purified by semi-preparative RP-HPLC (Nucleosil, gradient: 15-23 % B/A over 16 min), 4.06 µmol were obtained (yield = 16.2 %, UV titration at 280 nm, molar extinction coefficient = 1290 $L.mol^{-1}.cm^{-1}$).

19:

ESI-HRMS (*m/z*): $[MH]^+$ calcd for $C_{104}H_{173}N_{32}O_{36}S_5$: 2606.1293, found: 2606.1277.
HPLC: retention time: 3.33 min (Chromolith, gradient: 10-40 % B/A over 5 min).

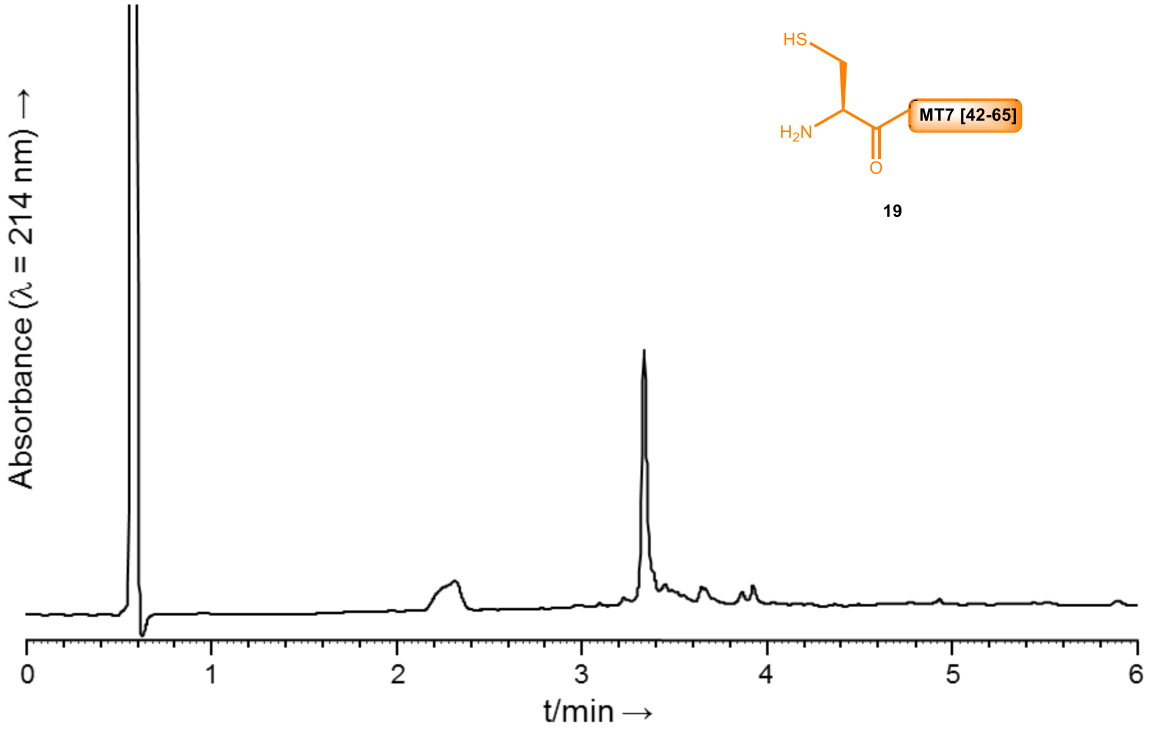

Supplementary figure S78: HPLC trace of crude **19**.

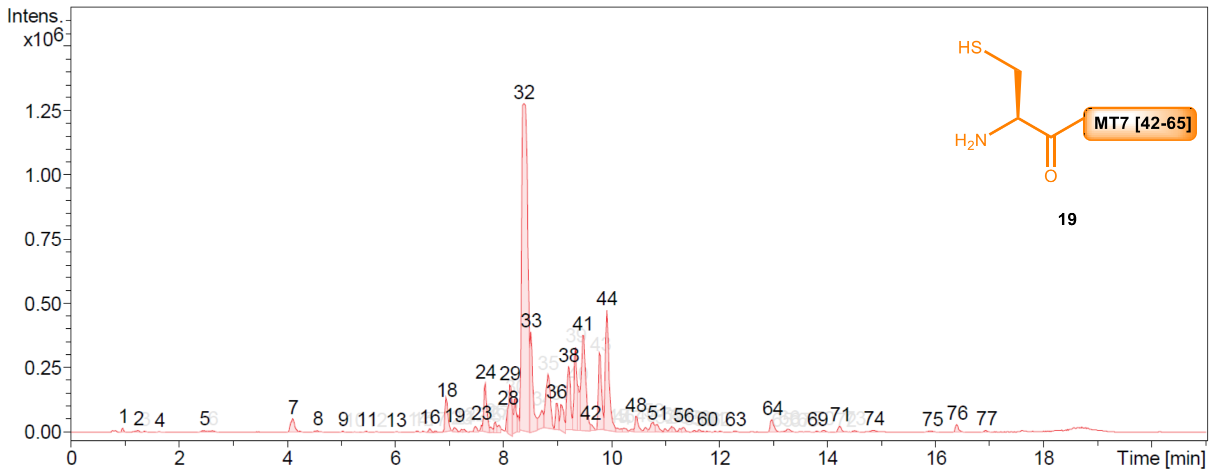

Supplementary figure S79: LC/MS analysis of crude **19**.

Peak (tr (min))	$[MH]^+$ (*m/z*) Calc.	$[MH]^+$ (*m/z*) found	Attributed to
7 (4.11)	1013.3855	1013.3823	Ac-[57-65]
18 (6.96)	1240.5125	1240.5095	Ac-[55-65]
24 (7.67)	1610.7089	1610.7057	Ac-[52-65]
28 (8.09)	1339.5809	1339.5755	Ac-[54-65]
32 (8.38)	2606.1293	2606.1277	**19**
33 (8.51	-	2588.1143	Not attributed
35 (8.83)	2198.9997	2198.9964	Ac-[47-65]
38 (9.20)	-	1886.7800	Not attributed
39 (9.34)	2662.1919	2662.1880	**19** + 56 Da (*t*Bu)
41 (9.41)	2662.1919	2662.1884	**19** + 56 Da (*t*Bu)
43 (9.79)	2662.1919	2662.1869	**19** + 56 Da (*t*Bu)
44 (9.91)	2662.1919	2662.1872	**19** + 56 Da (*t*Bu)

Supplementary table S5: Attribution of selected peaks observed in LC/MS analysis of crude **19**.

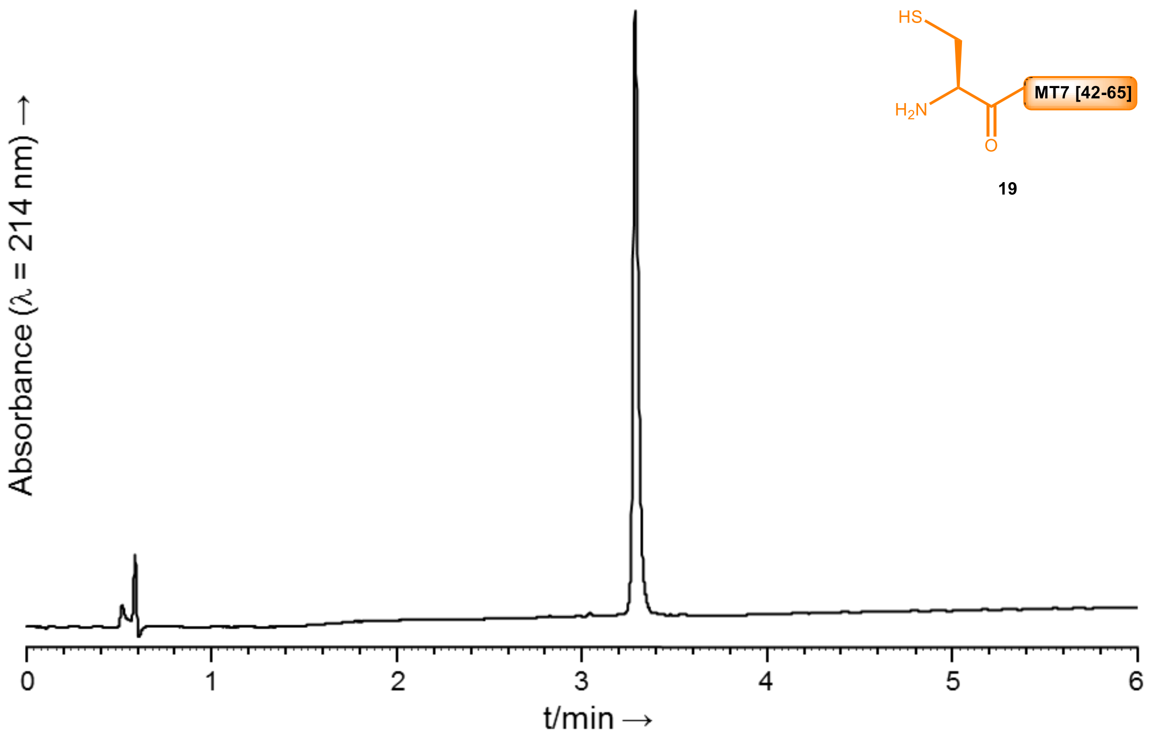

Supplementary figure S80: HPLC trace of pure **19**.

11b- Synthesis of crypto-thioester peptide MT7 [1-41]-(Hnb)C(S*t*Bu)G-NH$_2$ (**18**)

Amino acid sequence of the MT7 [1-41]-(Hnb)C(S*t*Bu)G-NH$_2$ segment:

H-LTCVKSNSIWFPTSEDCPDGQNLCFKRWQYISPRMYDFTRG(Hnb)C(S*t*Bu)G-NH$_2$

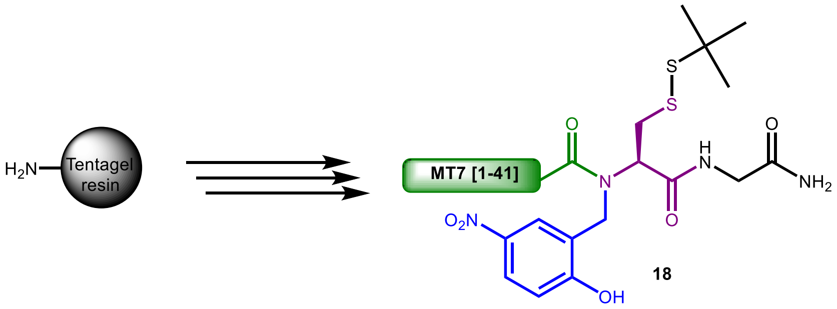

Supplementary scheme S32: Synthesis of crypto-thioester peptide MT7 [1-41]-(Hnb)C(S*t*Bu)G-NH$_2$ **18**.

Tentagel R resin (476 mg, 0.21 mmol/g, 0.1 mmol) was introduced into a polypropylene syringe fitted with a polypropylene frit and a PTFE stopcock and swollen with NMP. Fmoc group was removed by three successive treatments with 20% piperidine in NMP (10 mL) for 3 min. Then, Fmoc-Rink-OH (270 mg, 0.5 mmol, 5 equiv.) and HATU (190 mg, 0.5 mmol, 5 equiv.) were dissolved in NMP (2 mL) prior to addition of *i*Pr$_2$NEt (175 µL, 1 mmol, 10 equiv.). The resulting solution was immediately added to the resin. The syringe was left for 1.5 h under gentle stirring, then the syringe was drained and the resin was washed with NMP. Fmoc group was removed by three successive treatments with 20% piperidine in NMP (10 mL) for 3 min. Fmoc-Gly-OH (297 mg, 1 mmol, 10 equiv.) and HCTU (372 mg, 0.90 mmol, 9 equiv.) were dissolved in NMP (2 mL) prior to addition of *i*Pr$_2$NEt (348 µL, 2 mmol, 20 equiv.). The resulting solution was immediately added to the resin. The syringe was left for 1 h under gentle stirring, then the syringe was drained and the resin was washed with NMP. Then, Fmoc group was removed by three successive treatments with 20% piperidine in NMP (10 mL) for 3 min. Fmoc-Cys(S*t*Bu)-OH (432 mg, 1 mmol, 10 equiv.) and HCTU (372 mg, 0.90 mmol, 9 equiv.) were dissolved in NMP (10 mL) prior to addition of *i*Pr$_2$NEt (348 µL, 2 mmol, 20 equiv.). The resulting solution was immediately added to the resin. The syringe was left for 1.5 h under gentle stirring, then the syringe was drained and the resin was washed with NMP. Fmoc group was removed by three successive treatments with 20% piperidine in NMP (10 mL) for 3 min. The *N*-2-hydroxy-5-nitrobenzyl group was then introduced following the general procedure (p S5). Then, the complete MT7 [1-41] sequence was installed on a quarter of the resin trough standard Fmoc-SPPS (25 µmol, protocol S3). After cleavage of the resin (protocol p S3), the crude product **18** was pre-purified using a Sep-Pak cartridge (Waters Vac 12cc, C$_{18}$ - 2g, washing: 50 mL 0 % B/A then 50 mL 20 % B/A and elution: 50 mL 40 % B/A) and then purified by semi-preparative RP-HPLC (Nucleosil, gradient: 35-43 % B/A over 8 min) 1.62 µmol were

obtained (yield of 12.96 %, UV titration at 280 nm, molar extinction coefficient = 15940 L.mol^{-1}.cm^{-1}).

18:

ESI-HRMS (*m/z*): $[MH]^+$ calcd for $C_{234}H_{345}N_{62}O_{67}S_6$: 5287.3819, found: 5287.3818.
HPLC: retention time: 2.82min (Chromolith, gradient: 30-50 % B/A over 5 min).

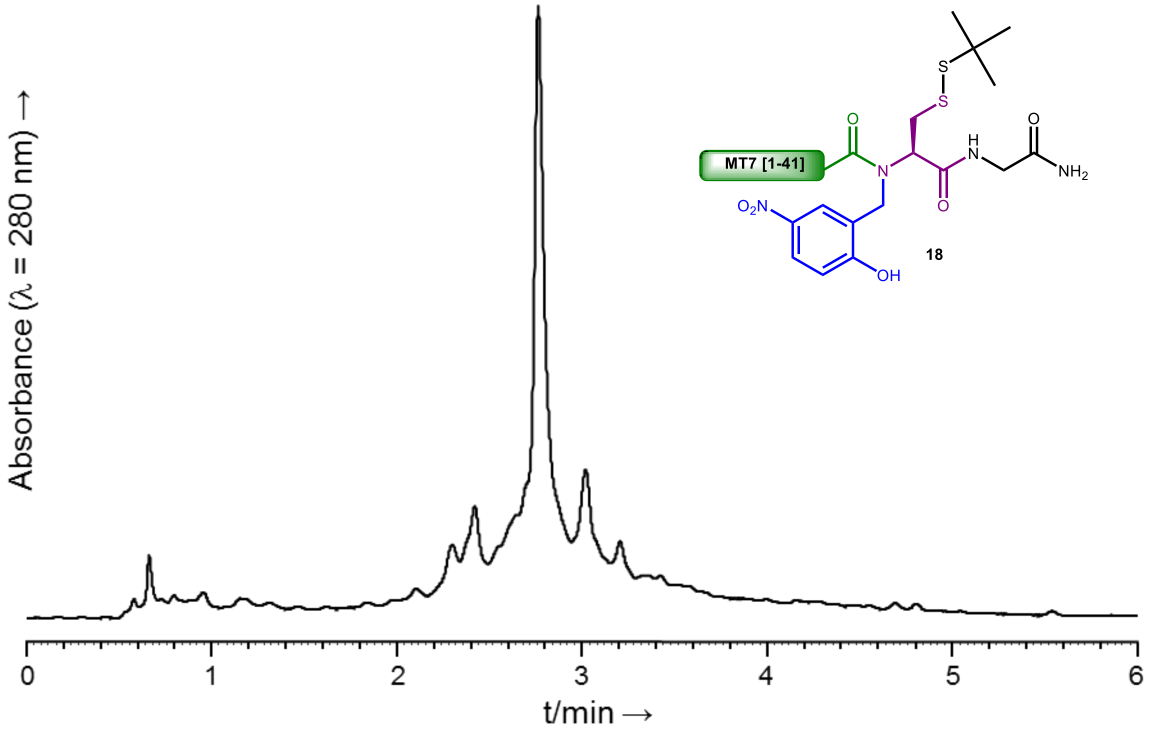

Supplementary figure S81: HPLC trace of crude **18**.

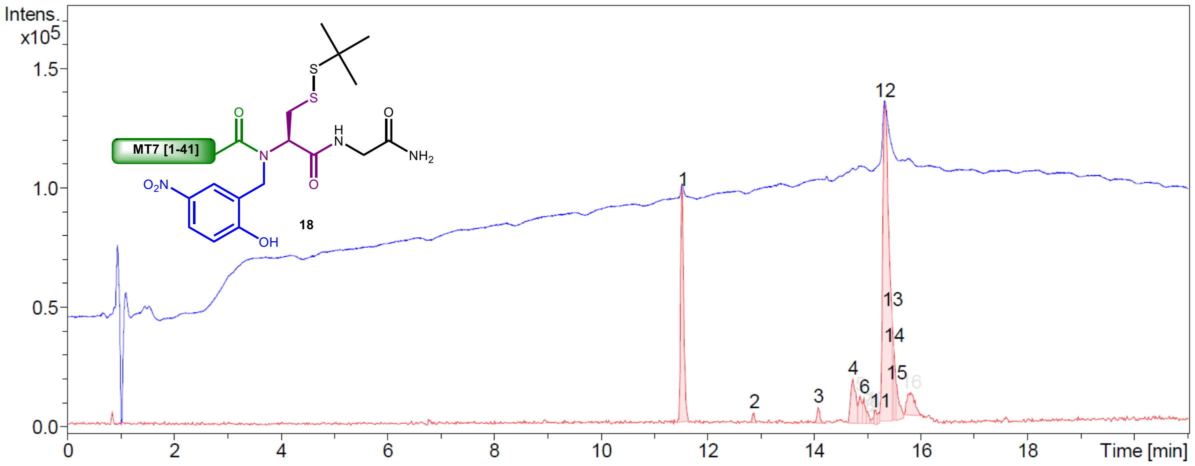

Supplementary figure S82: LC/MS analysis of crude **18**.

Peak (tr (min))	$[MH]^+$ (*m/z*) Calc.	$[MH]^+$ (*m/z*) found	Attributed to
1 (11.53)	-	1826.8617	Not attributed
2 (12.87)	-	1882.9243	Not attributed
3 (14.06)	-	614.2409	Not attributed
4 (14.72)	-	3479.5302	Not attributed
5 (14.86	5303,3768	5303.3519	**18** +16 Da (Oxidized Met)
10 (15.14)	2691,2423	2691.2184	Ac-[25-41]-(Hnb)C(S*t*Bu)-G-NH_2
12 (15.31)	5287.3819	5287.3818	**18**
13 (15.45)	-	5287.3423	Not attributed
14 (15.48)	-	5290.3463	Not attributed
16 (15.80)	5343.4444	5343.4466	**18** + 56 Da (*t*Bu)

Supplementary table S6: Attribution of selected peaks observed in LC/MS analysis of crude **18**.

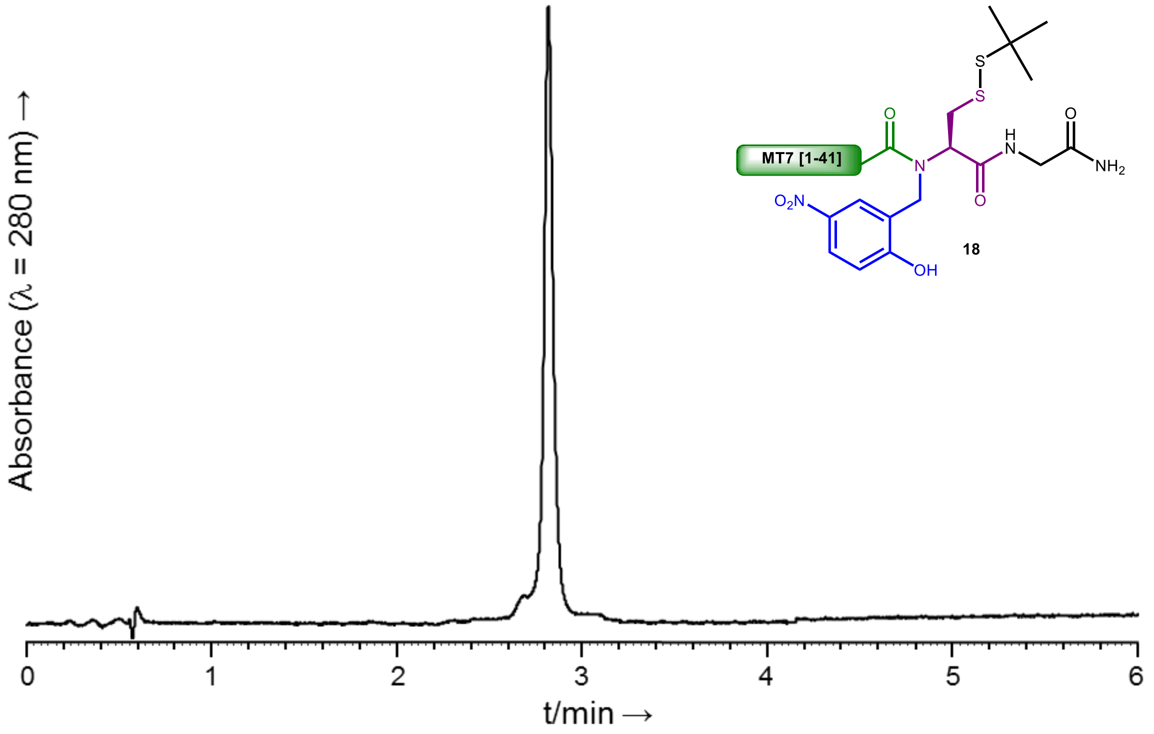

Supplementary figure S83: HPLC trace of pure **18**.

11c- NCL

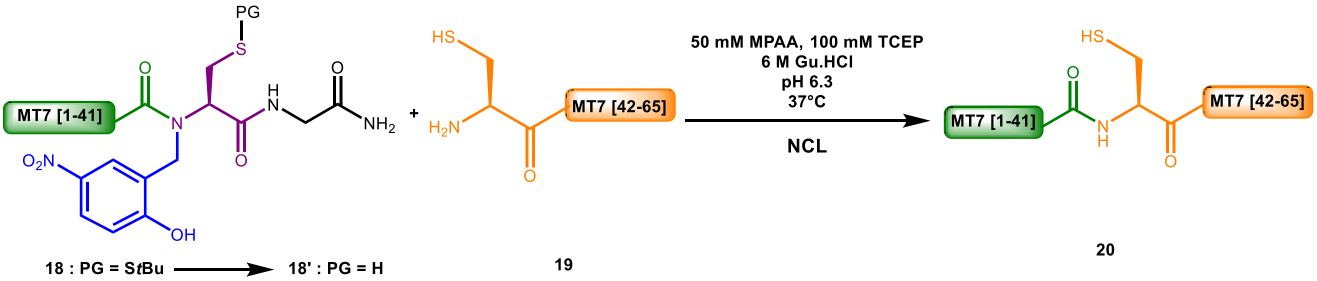

Supplementary scheme S33: NCL to obtain MT7 [1-65] **20**.

170 µL of a degassed 0.2 M pH 6.3 sodium phosphate buffer containing 25 mM MPAA, 50 mM TCEP and 6 M guanidine hydrochloride were added to 1.05 mg of the MT7 [1-41]-(Hnb)C(S*t*Bu)G-NH_2 peptide **18** (0.168 µmol, final concentration 1 mM) and 0.66 mg of the MT7 [42-65] peptide **19** (0.218 µmol, 1.3 equiv., final concentration 1.33 mM) under argon. The ligation was carried out at 37°C and monitored by RP-HPLC (Chromolith, gradient: 10-60% B/A over 6 min). For this, aliquots of 2 µL were diluted in 108 µL of 1.5% TFA in water and 100 µL were injected to the HPLC. After 24h, the reaction mixture was acidified with TFA (10 µL), 1 mL solvent A and 200 µL solvent B. this solution was extracted with diethylether (4 × 10 mL) to remove the MPAA. TCEP was added (final 100 mM) and the pH was adjusted to 5.0; after 20 min the pH was adjusted to 1 and the ligation product **20** was purified by semi-preparative RP-HPLC (Nucleosil, gradient: 25-43 % B/A over 10 min). 23.4 nmol were obtained (yield = 14 %, UV titration at 280 nm, molar extinction coefficient = 14870 $L.mol^{-1}.cm^{-1}$).

20:

ESI-HRMS (*m/z*): $[MH]^+$ calcd for $C_{322}H_{493}N_{90}O_{98}S_9$: 7476.3847, found: 7476.4022.
HPLC: retention time: 4.34min (Chromolith, gradient: 10-60% B/A over 6 min).

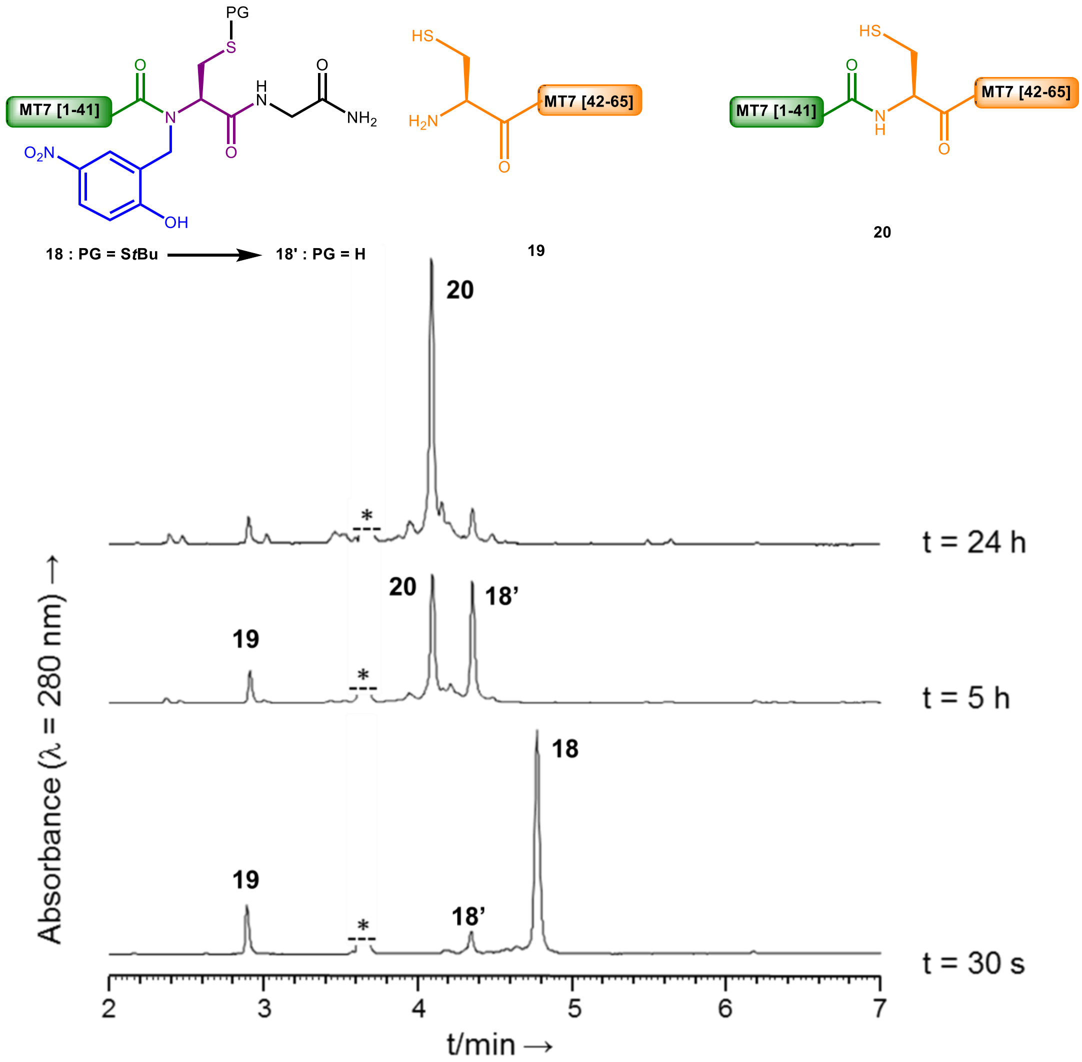

Supplementary figure S84: NCL of MT7 [1-65] **20**. *: MPAA

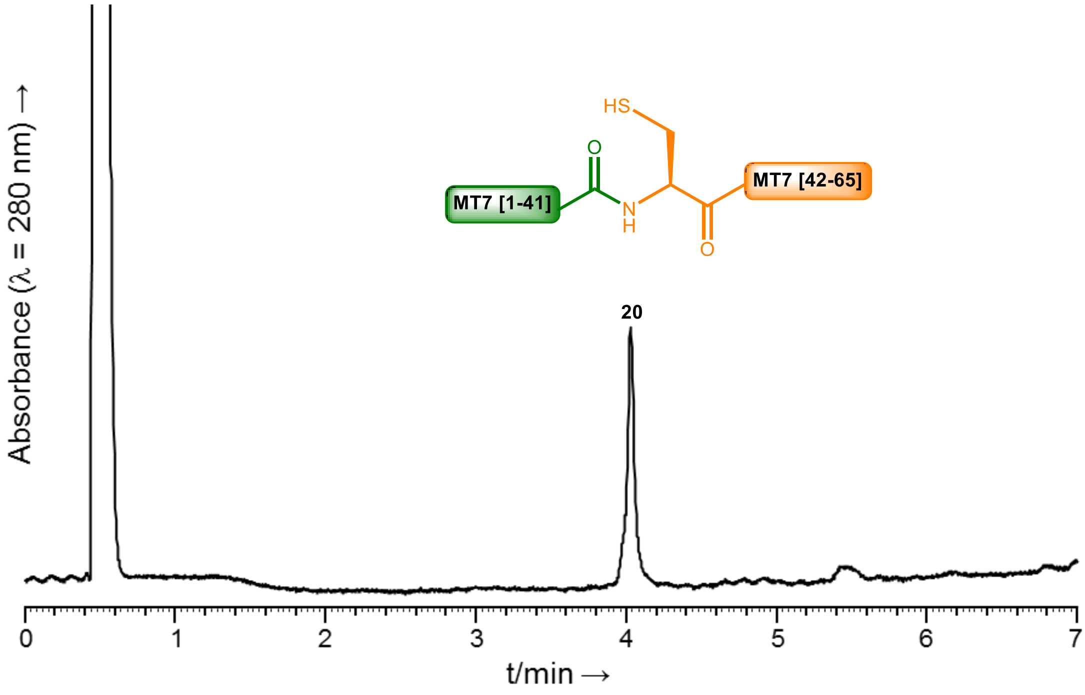

Supplementary figure S85: HPLC trace of pure MT7 [1-65] **20**.

12-Application to *Cg*-BigDef1

12a- Synthesis of cysteinyl peptide *Cg*-BigDef1 [57-93] (**22**)

Amino acid sequence of the *Cg*-BigDef1 [57-93] segment:

H-CANNRGWCRPTCFSHEYTDWFNNDVCGSYRCCRPGRR-NH_2

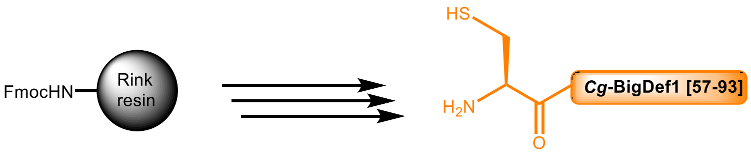

Supplementary scheme S34: Synthesis of cysteinyl peptide *Cg*-BigDef1 [57-93] peptide **22**.

Peptide **22** was obtained through automated SPPS (protocol p S3) starting from H-Rink amide Chemmatrix resin (200 mg, 0.52 mmol/g, 0.1 mmol); the elongation yield was 64% (determined by UV titration). After cleavage of a part of the resin (21 µmol) (protocol p S3), the product **22** was purified by semi-preparative RP-HPLC (Nucleosil, gradient: 25-38 % B/A over 13 min) 3.03 µmol were obtained (yield = 14.4%, UV titration at 280 nm, molar extinction coefficient = 13580 L.mol^{-1}.cm^{-1}).

22:

ESi-HRMS (*m/z*): $[MH]^+$ calcd for $C_{185}H_{271}N_{64}O_{53}S_6$: 4428.8802, found: 4428.8855.
HPLC: retention time: 3.57 min (Chromolith, gradient: 23-34 % B/A over 5 min).

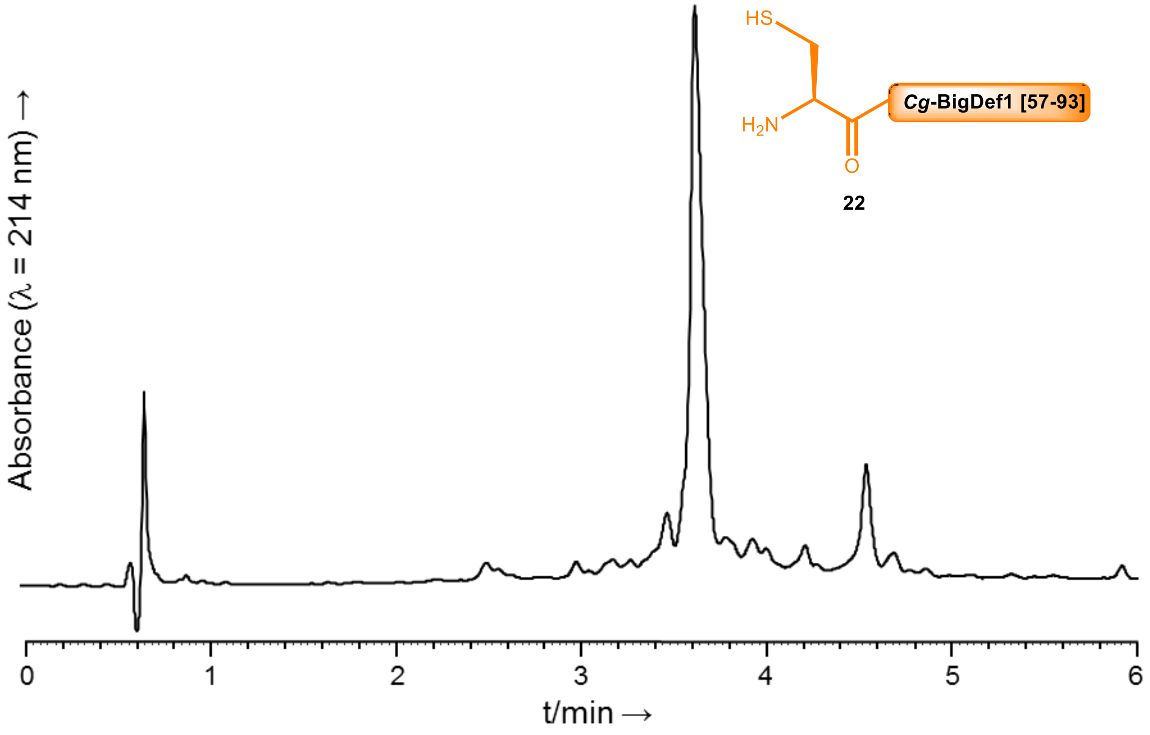

Supplementary figure S86: HPLC trace of crude **22**.

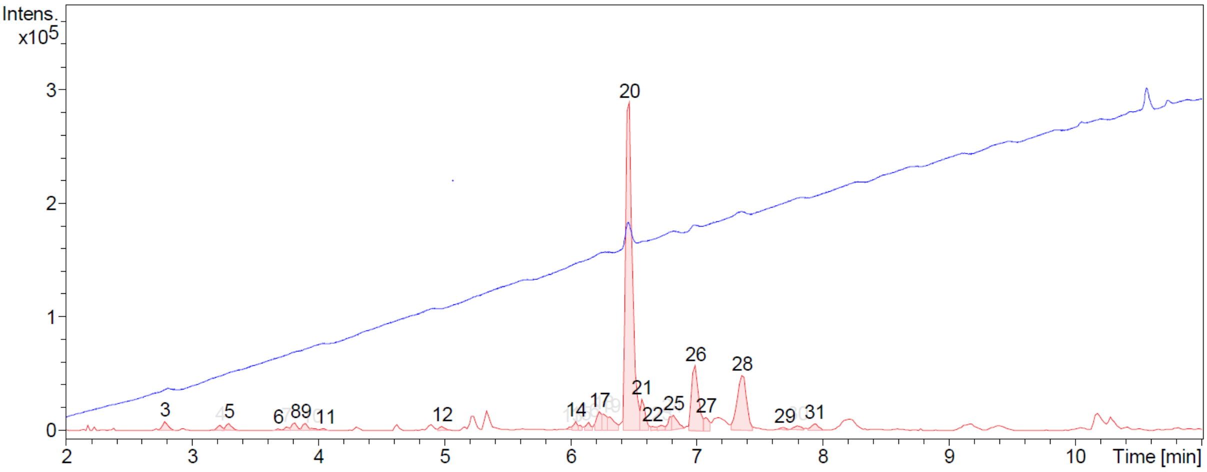

Supplementary figure S87: LC/MS analysis of crude **22**.

Peak (tr (min))	[MH]$^+$ (*m/z*) Calc.	[MH]$^+$ (*m/z*) found	Attributed to
1 (1.79)	888.4409	888.4405	Ac-[87-93]
2 (1.82)	888.4409	888.4401	Ac-[87-93]
3 (2.79)	1351.6588	1351.6580	Ac-[83-93]
4 (3.22)	1454.6680	1454.6670	Ac-[82-93]
7 (3.75)	1896.8492	1896.8480	Ac-[78-93]
8 (3.81)	1782.8063	1782.8043	Ac-[79-93]
9 (3.90)	1668.7634	1668.7612	Ac-[80-93]
16 (6.14)	2229.9969	2229.9911	Ac-[76-93]
20 (6.46)	4428.8802	4428.8855	**22**
23 (6.72)	2738.1775	2738.1721	Ac-[72-93]
26 (6.99)	4484.9481	4484.9377	**22** + 56 Da (*t*Bu)
28 (7.36)	3410.4465	3410.4426	Ac-[66-93]

Supplementary table S7: Attribution of selected peaks observed in LC/MS analysis of crude **22**.

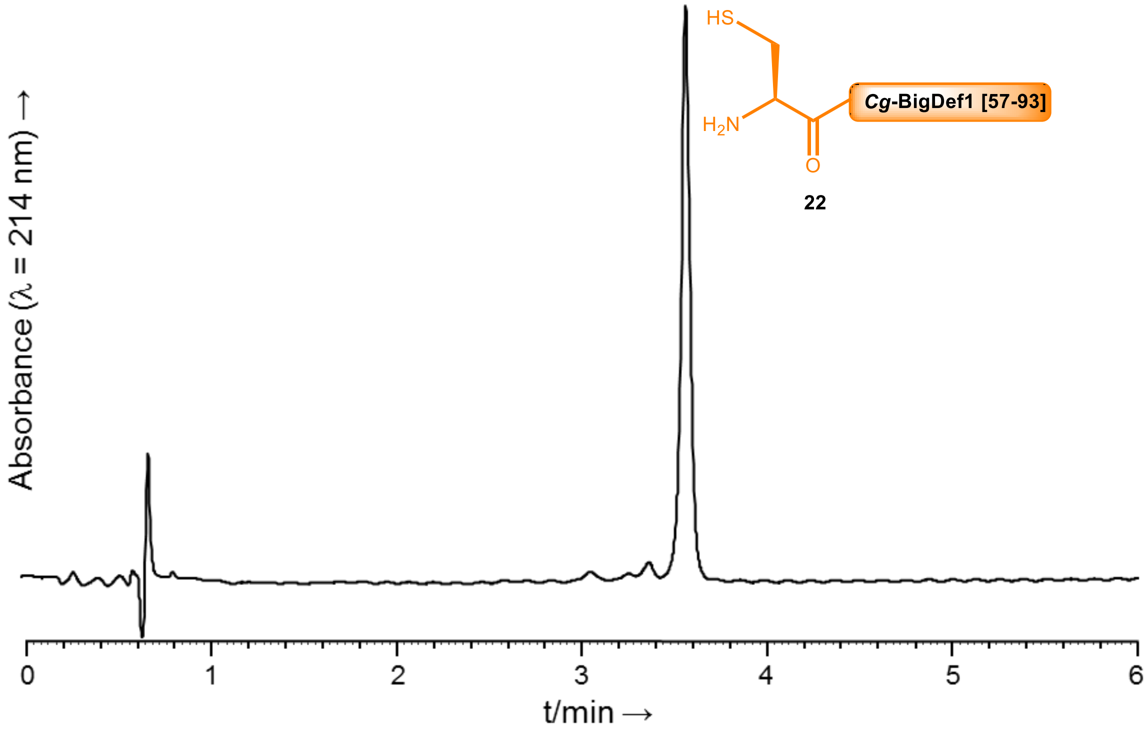

Supplementary figure S88: HPLC trace of pure **22**.

12b- Synthesis of crypto-thioester peptide*Cg*-BigDef1 [1-56]-(Hnb)C(S*t*Bu)G-NH_2 (**21**)

Amino acid sequence of the *Cg*-BigDef1 [1-56]-(Hnb)C(S*t*Bu)G-NH_2 segment:

XAQALLPIASYAGLTVSAPVFAALVTVYGAYALYRYNIRRRENSYQRIRSDHDSHS(Hnb)C(S*t*Bu)G-NH_2

X = pyroglutamic acid

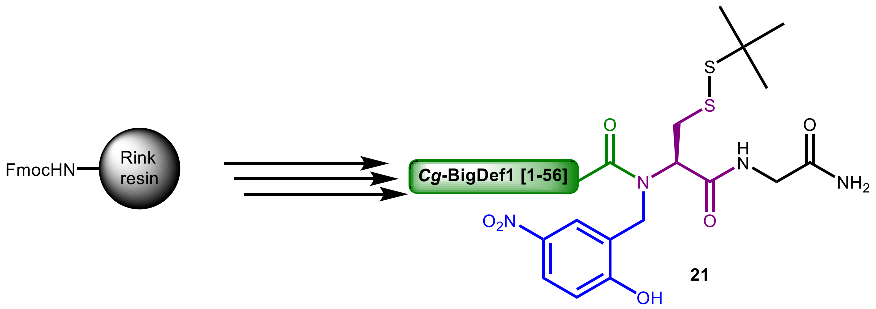

Supplementary scheme S35: Synthesis of crypto-thioester peptide *Cg*-BigDef1 [1-56]-(Hnb)C(S*t*Bu)G-NH_2 peptide **21**.

Peptide **21** was obtained through automated SPPS (protocol p S3) and using the automated reductive amination (protocol p S34) starting from Fmoc-Rink tentagel resin (120 mg, 0.21 mmol/g, 25 µmol) and for some amino acids double or triple coupling have been applied (see below) as determined as difficult couplings from a previous synthesis of the sequence not incorporating an (Hnb)Cys transthioesterification device. After cleavage of the resin (protocol p S3), the product **21** was purified by semi-preparative RP-HPLC (Nucleosil, gradient: 48-58 % B/A over

30 min) 1.69 µmol were obtained (yield = 6.8 %, UV titration at 280 nm, molar extinction coefficient = 10100 L.mol^{-1}.cm^{-1}).

XAQAL**L**P**I**A**SY**AGLTVS**A**PVFAALVTVYGAYALYRYNIRRRENSYQRIRSDHDSH**S**(Hnb)C(S*t*Bu)G-NH$_2$
X = pyroglutamic acid; green = double coupling, blue = triple coupling

21:
ESI-HRMS (*m/z*): [MH]$^+$ calcd for $C_{298}H_{457}N_{86}O_{86}S_2$: 6680.3472, found: 6680.3554
HPLC: retention time: 5.40 min (Chromolith, gradient: 20-70 % B/A over 6 min).

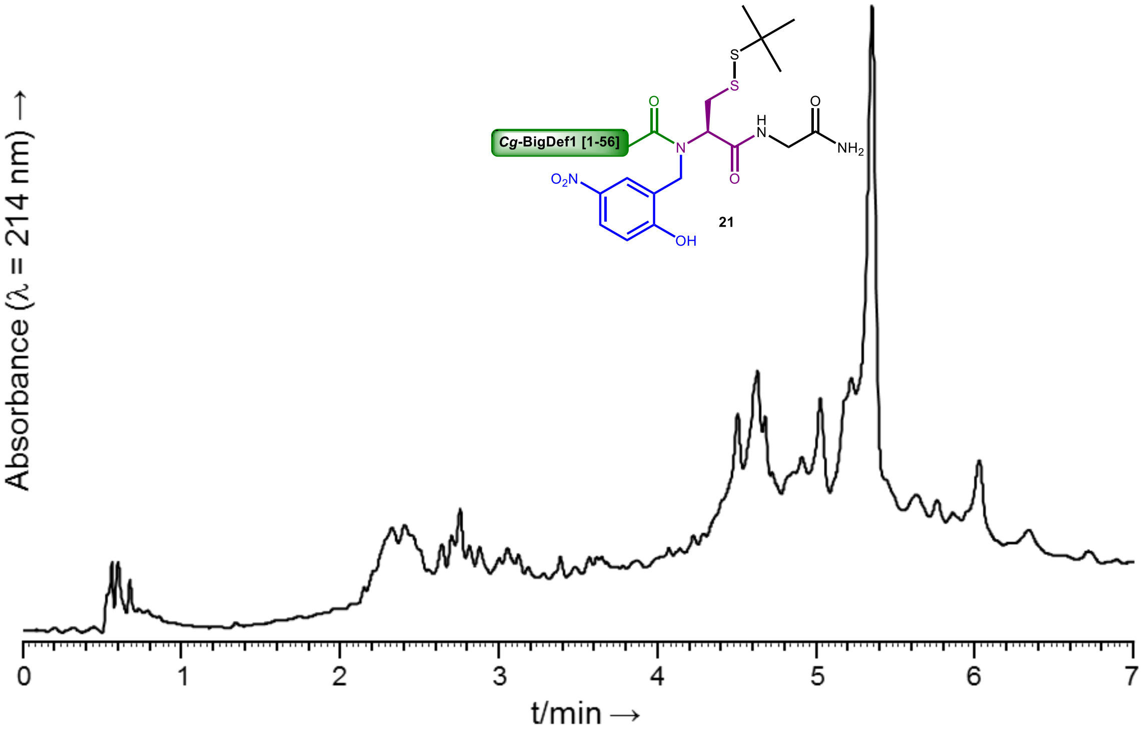

Supplementary figure S89: HPLC trace of crude **21**.

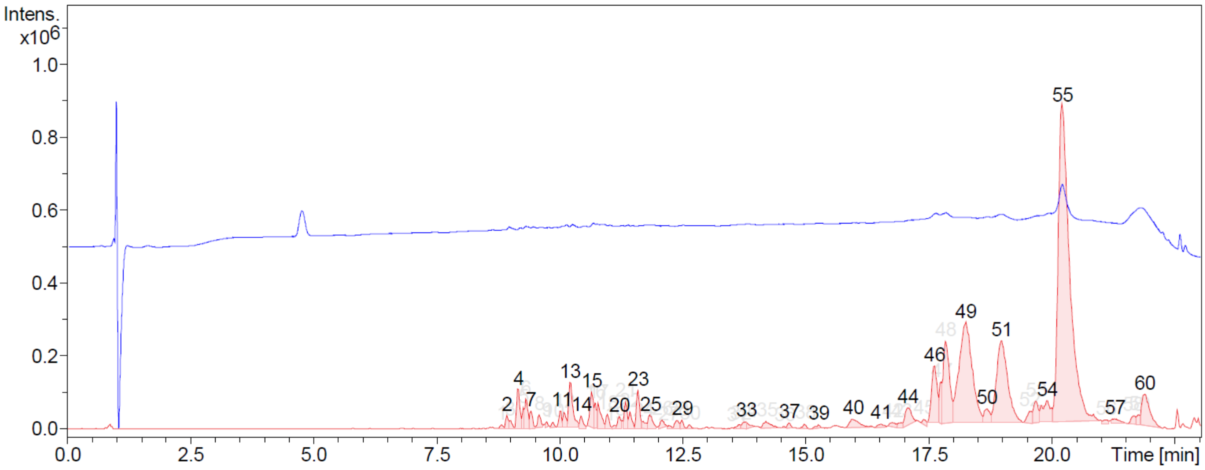

Supplementary figure S90: LC/MS analysis of crude **21**.

Peak (tr (min))	$[MH]^+$ (*m/z*) Calc.	$[MH]^+$ (*m/z*) found	Attributed to
4 (9.16)	2966.3611	2966.3649	Ac-[37-56]-(Hnb)C(S*t*Bu)-G-NH_2
13 (10.21)	-	3545.6465	Not attributed
15 (10.65)	3923.8319	3923.8386	Ac-[29-56]-(Hnb)C(S*t*Bu)-G-NH_2
23 (11.59)	1493,5902	1493.5893	Ac-[48-56]-(Hnb)C(S*t*Bu)-G-NH_2
46 (17.61)	5583.7555	5583.7657	Ac-[12-56]-(Hnb)C(S*t*Bu)-G-NH_2
47 (17.75)	5904.8880	5904.8903	Ac-[9-56]-(Hnb)C(S*t*Bu)-G-NH_2
48 (17.83)	5746.8189	5746.8267	Ac-[11-56]-(Hnb)C(S*t*Bu)-G-NH_2
49 (18.24)	4984.4277	4984.4364	Ac-[19-56]-(Hnb)C(S*t*Bu)-G-NH_2
51 (18.97)	6115.0248	6115.0354	Ac-[7-56]-(Hnb)C(S*t*Bu)-G-NH_2
55 (20.20)	6680.3472	6680.3554	**21**
60 (21.88)	-	6776.3467	Not attributed

Supplementary table S8: Attribution of selected peaks observed in LC/MS analysis of crude **21**.

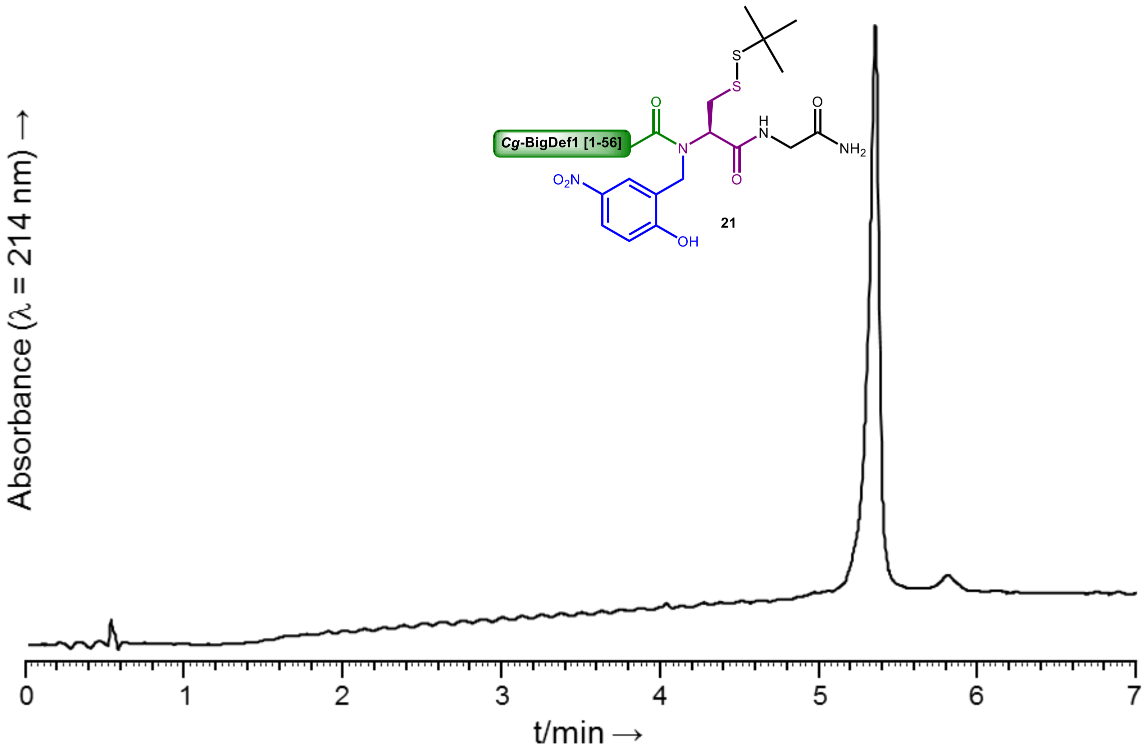

Supplementary figure S91: HPLC trace of pure **21**.

12c- NCL

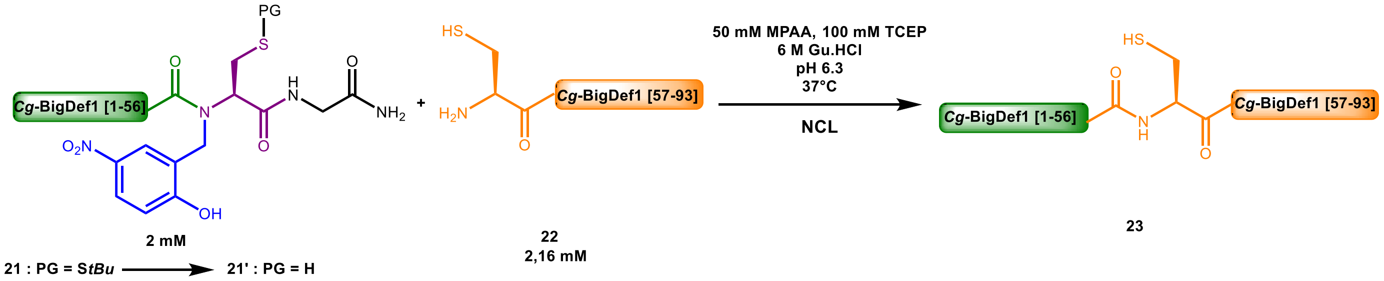

Supplementary scheme S36: NCL to obtain *Cg*-BigDef1 [1-93] **23**.

352.5 µL of a degassed 0.2 M pH 6.3 sodium phosphate buffer containing 50 mM MPAA, 100 mM TCEP and 6 M guanidine hydrochloride were added to 5.38 mg of the *Cg*-BigDef1 [1-56]-(Hnb)C(S*t*Bu)G-NH_2 peptide **21** (0.705 µmol, final concentration 2 mM) and 4 mg of the *Cg*-BigDef1 [57-93] peptide **22** (0.763 µmol, 1.08 equiv., final concentration 2.16 mM) under argon. The ligation was carried out at 37°C and monitored by RP-HPLC (Chromolith, gradient: 20-70 % B/A over 6 min). For this, aliquots of 2 µL were diluted in 108 µL of 1.5% TFA in water and 100 µL were injected to the HPLC.After 24h, the reaction mixture was acidified with TFA (30 µL) and 2 mL of 1.5% TFA in water; this solution was extracted with diethylether (4 × 10 mL) to remove the MPAA. The ligation product precipitated; after centrifugation and removal of the supernatant, the solid was diluted in 400 µL of solvent B and 1.2 mL of solvent A (V total = 1.6 mL); 45 mg of TCEP were added (final 100 mM) and the pH was adjusted to 4.5, after 20 min the pH was adjusted to 1 and the ligation product **SXX** was purified by semi-preparative RP-HPLC (Nucleosil, gradient: 50-60 % B/A over 10 min). 130 nmol were obtained (yield = 18.4 %, UV titration at 280 nm, molar extinction coefficient = 21320 $L.mol^{-1}.cm^{-1}$).

23:

ESI-HRMS (*m/z*): $[MH]^+$ average mass calcd for $C_{467}H_{703}N_{146}O_{134}S_6$: 10699.0090, found: 10699.1541

HPLC: retention time: 4.96 min (Chromolith, gradient: 20-70 % B/A over 6 min).

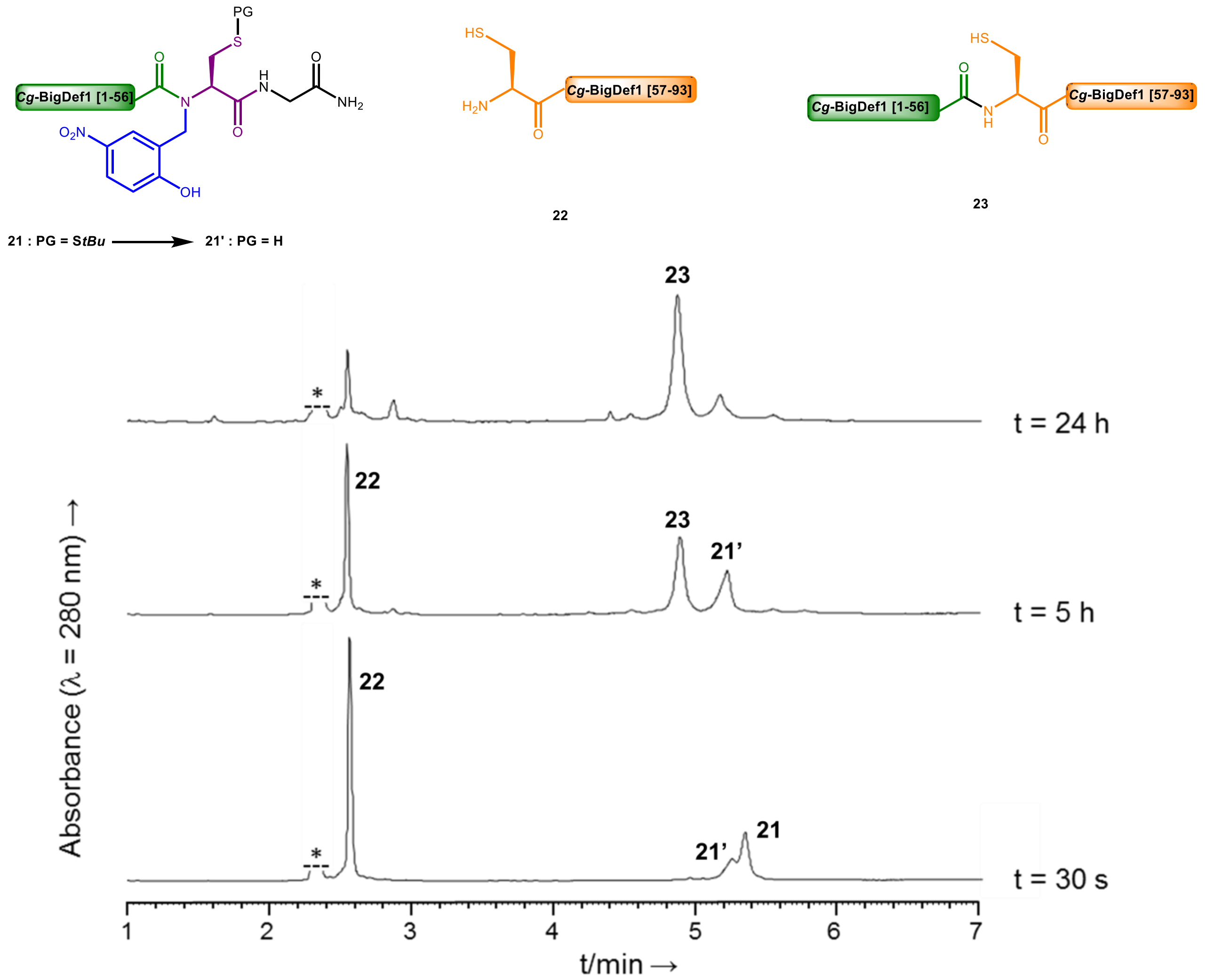

Supplementary figure S92: NCL reaction to obtain *Cg*-BigDef1 [1-93] **23**. *: MPAA

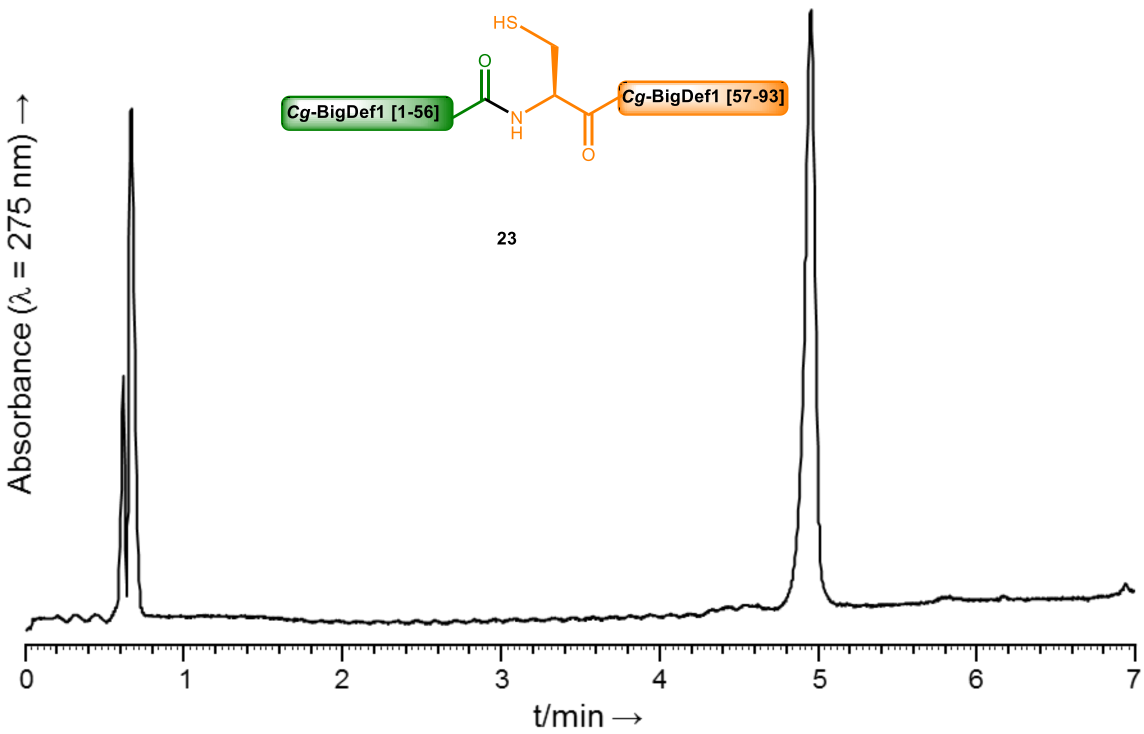

Supplementary figure S93: HPLC trace of pure *Cg*-BigDef1 [1-93] **23**.
